# Dopaminergic neurons linking threat processing to cardiac modulation and locomotor responses

**DOI:** 10.7554/eLife.111256

**Published:** 2026-11-05

**Authors:** Masato Tsuji, Daisuke Jinkoma, Yuki Uemura, Ayako Ogasawara, Kazuo Emoto

**Affiliations:** 1 Department of Biological Sciences, Graduate School of Science, The University of Tokyo Tokyo Japan; 2 International Research Center for Neurointelligence (WPI-IRCN), The University of Tokyo Tokyo Japan; Tohoku University Japan; University of California, Los Angeles United States

**Keywords:** cardiac modulation, dopaminergic neurons, defensive behavior, heart-brain interactions, *D. melanogaster*

## Abstract

Heartbeat and behavior are tightly coordinated during defensive states, yet the neuronal mechanisms linking threat processing to cardiac modulation—and the potential contribution of cardiac dynamics to behavioral output—remain poorly understood. Here, using simultaneous recordings of heartbeat and behavior in tethered *Drosophila*, we show that mechanical threat triggers locomotion together with rapid cardiac deceleration. We identify two pairs of dopaminergic neurons, termed DA-WED neurons, that mediate this cardiac response. Silencing these neurons markedly attenuates threat-induced cardiac deceleration, whereas optogenetic activation induces cardiac deceleration in the absence of threat. Two-photon calcium imaging further shows that DA-WED neurons are activated by mechanical threat, and manipulating their activity bidirectionally modulates threat-induced locomotion. Linking cardiac dynamics to behavior, direct optogenetic manipulation of cardiomyocytes that quantitatively reproduces threat-evoked cardiac deceleration is accompanied by increased locomotion. Together, these results identify a dopaminergic descending pathway that links threat processing to cardiac modulation and suggest that cardiac dynamics may contribute to shaping defensive behavior.

## Introduction

Heartbeat dynamics are tightly coordinated with behavioral state. Defensive states, for example, are accompanied by coordinated modulation of cardiac activity, including transient cardiac deceleration during threat detection and cardiac acceleration during active coping in mammals (Hagenaars et al., 2014). Such tight coordination between behavioral state and cardiac dynamics is likely advantageous for survival: cardiac deceleration may minimize the brain-wide representations of the heartbeat (Babo-Rebelo et al., 2016; Jammal Salameh et al., 2024; Azzalini et al., 2021; Ran et al., 2022) to sharpen sensory processing (Hagenaars et al., 2014; Lang et al., 2000), whereas cardiac acceleration boosts the delivery of metabolic resources to muscles (Gordan et al., 2015). Although much of the existing work on heart–behavior coupling has focused on mammals, comparable threat-associated cardiac modulation has also been reported in invertebrates. In *Drosophila* and decapod crustaceans, for instance, sensory stimuli elicit time-locked heart rate changes that parallel defensive behaviors, suggesting that coordinated heart–behavior responses are evolutionarily widespread (Pérez-Schuster et al., 2024; Burnovicz et al., 2009; Barrios et al., 2021). Despite this well-documented coupling, the circuit mechanisms linking threat processing in the brain to cardiac modulation—and whether cardiac dynamics contribute to behavioral output—remain incompletely understood.

In mammals, heartbeat is governed by sympathetic and parasympathetic branches of the autonomic nervous system (ANS) that innervate the heart (Gordan et al., 2015; Koba et al., 2022; Green et al., 2007; Matsukawa, 2012; Yoshimoto et al., 2024; Wink et al., 2020; White and Raven, 2014; Rajendran et al., 2019; Silvani et al., 2016). Sympathetic output that accelerates the heartbeat arises from brainstem and hypothalamic circuits and projects through spinal intermediates to the heart (Gordan et al., 2015). On the other hand, the parasympathetic nervous system, which decelerates the heartbeat (Gordan et al., 2015; Prescott and Liberles, 2022), originates from the dorsal motor nucleus of the vagus nerve and nucleus ambiguus, and projects directly to the heart (Prescott and Liberles, 2022; Veerakumar et al., 2022). These autonomic pathways are engaged in threatened animals by the so-called fear circuitry in the brain (Silva et al., 2016; Headley et al., 2019; Gross and Canteras, 2012) to shape cardiac responses, typically producing cardiac deceleration followed by acceleration (Hagenaars et al., 2014; Kreibig, 2010; Battaglia and Thayer, 2022; Casto et al., 1989; Dielenberg et al., 2001; Signoret-Genest et al., 2023; Hashemi et al., 2019). Circuit-wise, the periaqueductal gray (PAG) is considered the output hub of the fear circuitry and modulates the heartbeat through the hypothalamus, midbrain, pons, and medulla oblongata (Farkas et al., 1998). In addition, hypothalamic nuclei such as the dorsomedial hypothalamus (DMH; Horiuchi et al., 2006) and the arcuate nucleus (ARC), as well as the anterior cingulate cortex (ACC; Alexander et al., 2020), also modulate the heartbeat upon threats (Koba et al., 2016; Nakamura et al., 2022; Hermans et al., 2013; Samuels et al., 2002; Rahmouni, 2016; Li et al., 1996; Mastrianni et al., 1989).

In parallel, ascending feedback from the heart to the brain has attracted increasing attention. Such interoceptive signaling (Lovelace et al., 2024) is conveyed by sensory neurons innervating the heart and aortic arch (Lovelace et al., 2024), which project to NTS and onward to hypothalamic and cortical regions including the paraventricular nucleus (PVN) of the hypothalamus Shivkumar et al., 2016, ACC, insular, and prefrontal cortex (Shivkumar et al., 2016). These pathways have been implicated in learned fear (Klein et al., 2021), anxiety-like behavior (Hsueh et al., 2023), or sleep (Yao et al., 2022). Together, these findings suggest that bidirectional communication between the brain and heart may shape physiological and behavioral responses during defensive states. However, how central circuits orchestrate threat-associated cardiac modulation, and whether cardiac dynamics influence defensive behavior, remain unresolved.

The fruit fly *Drosophila melanogaster* provides a genetically tractable system to dissect brain–body interactions. The *Drosophila* heart, an evolutionarily conserved organ (Han et al., 2006; Han and Olson, 2005; Sellin et al., 2006; Wessells and Bodmer, 2004), consists of two rows of cardiomyocytes forming a tubular structure that runs through the midline of the abdomen (Hillyer and Pass, 2020; Rotstein and Paululat, 2016). Like the mammalian heart, the *Drosophila* heart receives rich neuronal projections throughout (Dulcis and Levine, 2005; Dulcis and Levine, 2003). Recent work has shown that visual threat induces cardiac deceleration in *Drosophila* (Barrios et al., 2021), suggesting the existence of a neuronal mechanism in the brain that modulates the heart. However, the neuronal substrate of such a mechanism, and the behavioral consequences of cardiac modulation during threat, remain largely unknown.

We have recently reported that a train of air puffs elicits hyperactivity, avoidance of puffs, and aversion from an otherwise neutral visual object, suggesting that air puffs serve as mechanical threats (Tsuji et al., 2023). Building on this work, we established an experimental paradigm to assess how mechanical threat modulates heartbeat and behavior. Using this system, we identify two pairs of dopaminergic neurons in the brain, termed DA-WED neurons, that are responsible for threat-induced cardiac deceleration. We further examine how cardiac deceleration relates to threat-induced locomotion. Together, our findings identify two pairs of dopaminergic neurons that link threat processing in the brain to cardiac modulation and suggest that cardiac dynamics may contribute to shaping defensive behavior.

## Results

### Air puffs induce cardiac deceleration and locomotion

**Figure 1.**
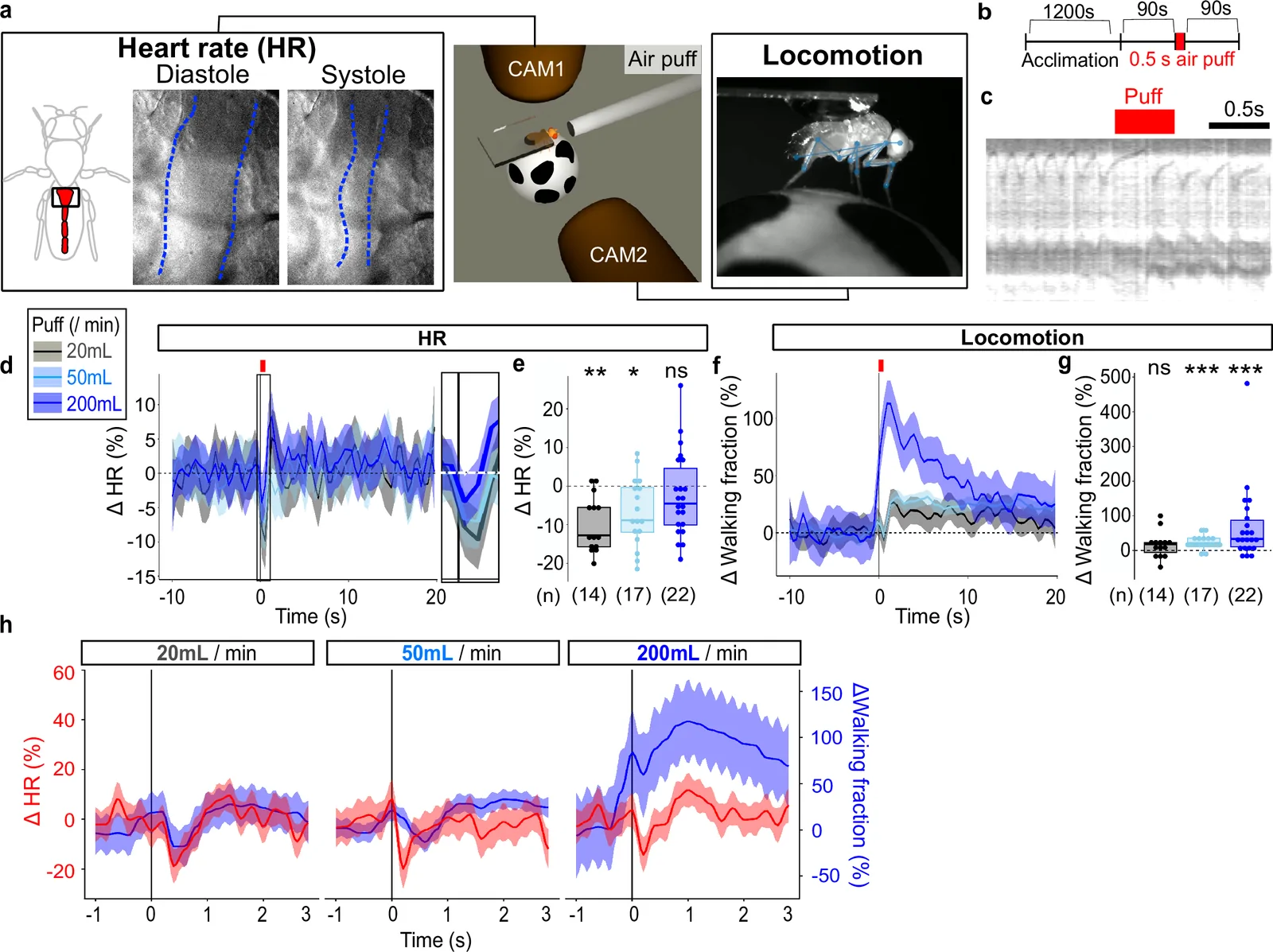
Mechanical threat induces cardiac deceleration and locomotion. (**a**) Schematics of the paradigm. The fly is fixed on an air-supported ball. Left: the heart rate (HR) was videotaped by an IR-sensitive camera from the dorsal side through the cuticle (‘CAM1’). Of the four cardiac chambers, our system videotaped the chamber closest to the thorax called the conical chamber. We could easily distinguish diastole and systole in the captured video (blue dashed lines indicate the heart wall). Right: simultaneously, locomotion was videotaped from the right side of the fly (‘CAM2’). Blue dots and lines indicate automatically tracked body parts. (**b**) The time course of the experiment. An air puff of 0.5 s was applied once. The trial was repeated 10 times for each fly, the results of which were averaged. (**c**) An example M-mode of the heartbeat. An air puff was applied during the red-shaded time window. (**d**) Time course of the HR (% change from baseline defined as the average during the 10 s preceding puff onset). N=14, 17, 22 for 20 mL/min (gray), 50 mL/min (sky blue), and 200 mL/min (dark blue), respectively. The inlet shows the magnified view of the cardiac deceleration indicated by the box. (**e**) Change in HR, calculated for each fly as the % difference between the mean fraction during the 1 s following stimulation onset and the mean fraction during the 10 s baseline period preceding stimulation. N=14, 17, 22 for 20 mL/min (gray), 50 mL/min (sky blue), and 200 mL/min (dark blue), respectively. **p<0.01, *p<0.05, n.s.: p>0.05, Wilcoxon’s signed rank test followed by Bonferroni correction. (**f**) Time course of the walking fraction (% change from baseline defined as the mean fraction during the 10 s preceding puff onset). N=14, 17, 22 for 20 mL/min (gray), 50 mL/min (sky blue), and 200 mL/min (dark blue), respectively. ***p<0.001, n.s.: p>0.05, Wilcoxon’s signed rank test followed by Bonferroni correction. (**g**) Change in walking fraction, calculated for each fly as the % difference between the mean fraction during the 10 s following stimulation onset and the mean fraction during the 10 s baseline period preceding stimulation. N=14, 17, 22 for 20 mL/min (gray), 50 mL/min (sky blue), and 200 mL/min (dark blue), respectively. ***p<0.001, n.s.: p>0.05, Wilcoxon’s signed rank test followed by Bonferroni correction. (**h**) Changes in HR and walking fraction jointly plotted for –1–3 s around the stimulus onset, for each puff strength. N=14, 17, 22 for 20, 50, and 200 mL/min, respectively.

We have previously reported that a train of air puffs serves as mechanical threats, eliciting a host of fear-like behaviors such as hyperactivity, avoidance of puffs, and aversion from an otherwise neutral visual object (Tsuji et al., 2023). This motivated us to test how the air puff might influence heartbeat and its relation to behavior. For this purpose, we developed a paradigm to monitor heartbeat and behavior simultaneously (Figure 1a). Our paradigm images the heart chamber closest to the thorax, called the conical chamber, which is visualized through the dorsal cuticle by cuticle-penetrating infrared light (Figure 1a, ‘CAM1’). Simultaneously, the fly’s behavior is recorded from the side of the fly (Figure 1a, ‘CAM2’). We further developed a set of software to automatically detect heartbeat and behavior (Video 1). Our heartbeat detection software takes two consecutive frames and calculates for each pixel the optical flow, which is used to quantify the overall relaxation (diastole) or shrinkage (systole) of the heart (Figure 1—figure supplement 1a–c). Based on such quantification, our software detects the timings at which the heart was shrunk most (systole peak), which were counted to obtain the heart rate (HR). Our analysis pipeline for behavior detection first tracks the coordinates of body parts (joints of each leg, eye, neck, haltere, rostral tip of the abdomen, all on the right side of the fly), utilizing SLEAP software (Pereira et al., 2022). The tracked coordinates are then fed into our custom support vector machine-based program to detect locomotion. Our analysis pipeline allowed us to visualize the HR and locomotion over time in a synchronized fashion.

**Figure 1—figure supplement 1.**
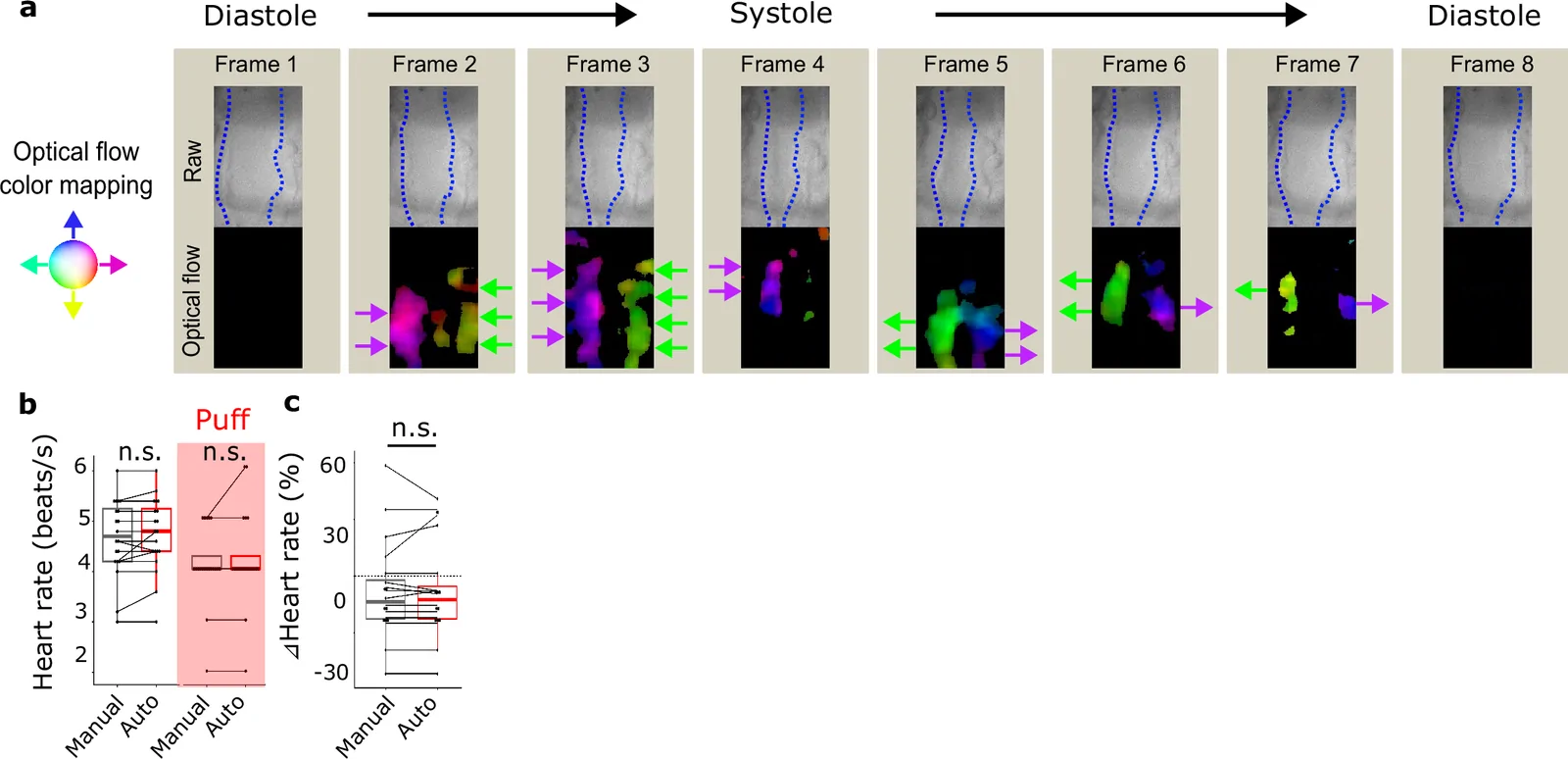
heartbeat detection. (**a**) The shown EIGHT consecutive frames capture an episode of ‘diastole ->systole ->diastole’ (‘Raw’, in which blue dotted lines indicate the outline of the conical chamber). Our software estimates the optical flow in these frames (‘Optical flow’, in which estimated directions are color-coded). Our software reveals a bout of contraction (frames 2–4), which is followed by a bout of relaxation (frames 5–8). Arrows indicate the optical flow direction for illustration purposes. (**b**) Plot comparing the HR calculated from manual count vs auto-detection, before and during puff application. Each line indicates identical flies. N=20. n.s.: p>0.05, two-tailed, paired t-test followed by Bonferroni correction. (**c**) Plot comparing the % change in HR (during vs pre-puff, 10 s each) calculated from manual count vs auto-detection. N=20. n.s.: p>0.05, two-tailed, paired t-test.

**Figure 1—figure supplement 2.**
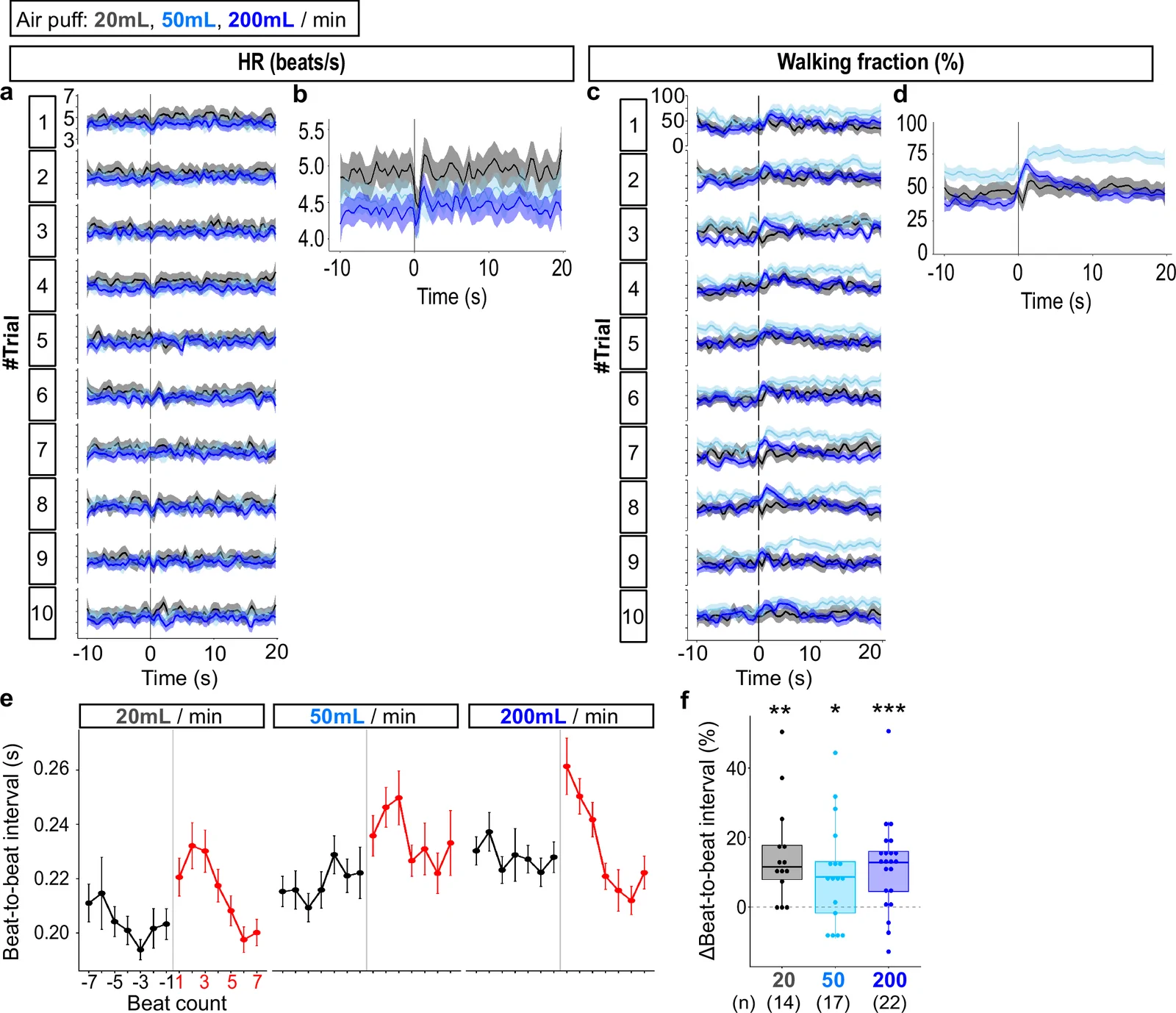
The effect of air puff on behavior and heartbeat. (**a**) Time course of the HR (beats/s) without averaging over 10 trials. Results of different puff strengths are superimposed (gray: 20 mL/min, sky blue: 50 mL/min, dark blue: 200 mL/min; different fly groups were used for each puff strength). N=14, 17, 22 for 20 mL/min (gray), 50 mL/min (sky blue), and 200 mL/min (dark blue), respectively. (**b**) Time course of the HR averaged over 10 trials for each fly. Results of different puff strengths are superimposed (gray: 20 mL/min, sky blue: 50 mL/min, dark blue: 200 mL/min; different fly groups were used for each puff strength). N=14, 17, 22 for 20 mL/min (gray), 50 mL/min (sky blue), and 200 mL/min (dark blue), respectively. (**c**) Time course of the walking fraction (%) without averaging over 10 trials. Results of different puff strengths are superimposed (gray: 20 mL/min, sky blue: 50 mL/min, dark blue: 200 mL/min; different fly groups were used for each puff strength). N=14, 17, 22 for 20 mL/min (gray), 50 mL/min (sky blue), and 200 mL/min (dark blue), respectively. (**d**) Time course of walking fraction (%) averaged over 10 trials for each fly. Results of different puff strengths are superimposed (gray: 20 mL/min, sky blue: 50 mL/min, dark blue: 200 mL/min; different fly groups were used for each puff strength). N=14, 17, 22 for 20 mL/min (gray), 50 mL/min (sky blue), and 200 mL/min (dark blue), respectively. (**e**) Beat-to-beat interval (in seconds) plotted against the beat count around puff onset (gray vertical line). Dots and bars indicate mean and SEM, respectively. N=14, 17, 22 for 20, 50, and 200 mL/min, respectively. (**f**) Change in beat-to-beat interval, calculated for each fly as the % difference between the mean interval during three beats following stimulation onset and the mean interval during three beats preceding stimulation. N=14, 17, 22 for 20, 50, and 200 mL/min, respectively. ***p<0.001, **p<0.01, *p<0.05, Wilcoxon’s signed rank test followed by Bonferroni correction.

**Figure 1—figure supplement 3.**
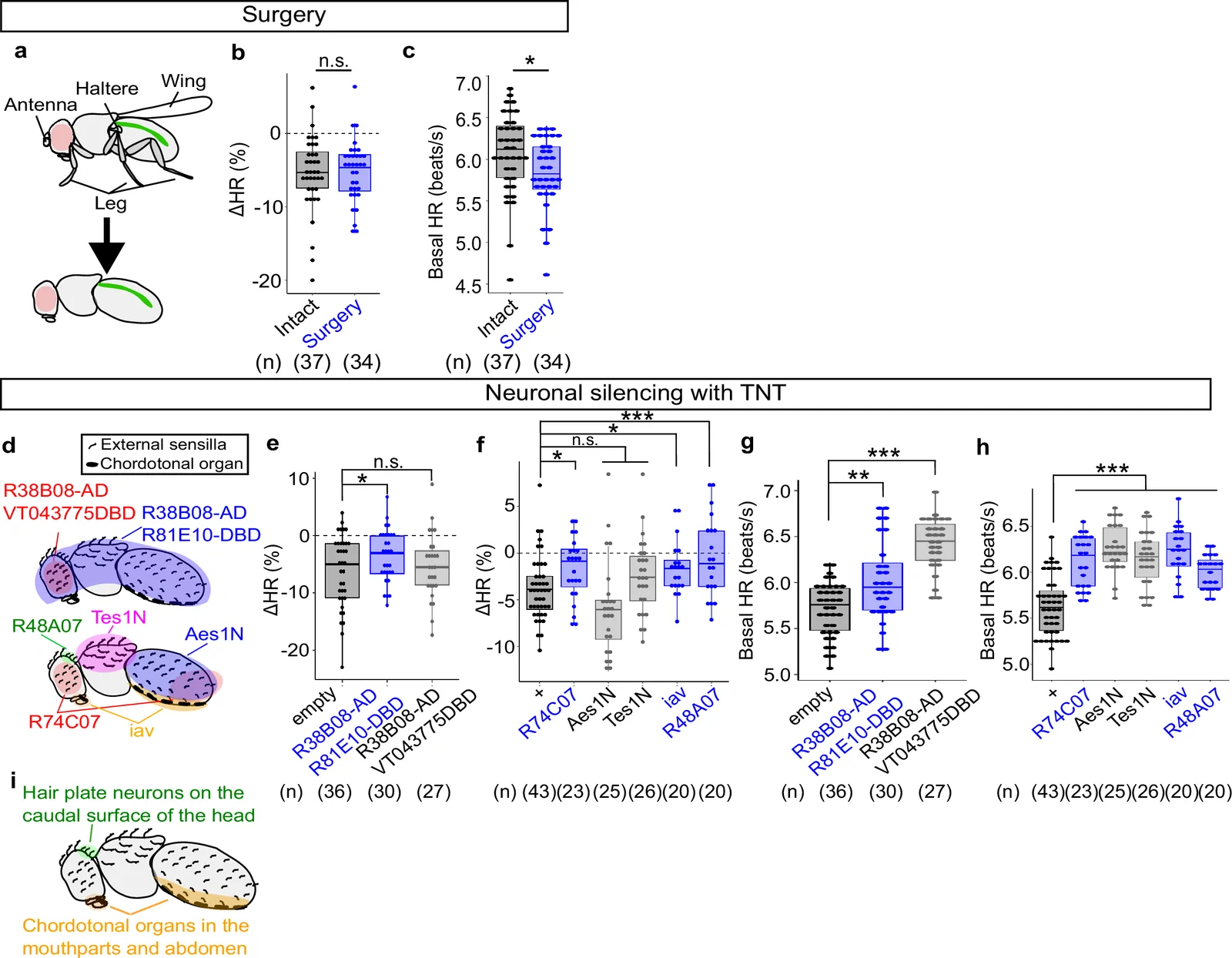
Somatosensory systems required for puff-induced cardiac deceleration. (**a**) *Drosophila* possesses multiple mechanosensory organs throughout the body, including legs, halteres, wings, and the body trunk. To first distinguish which of these body parts may be indispensable for puff-induced cardiac deceleration, we surgically removed legs, halteres, wings, and tested puff-induced cardiac deceleration. Green indicates the heart. (**b**) Change in HR, calculated for each fly as the % difference between the mean HR during the 10 s stimulation period and the HR during the 10 s baseline period preceding stimulation. N=37, 34, for intact flies, surgery flies, respectively. n.s.: p>0.05, Wilcoxon’s rank sum test. (**c**) Baseline HR. N=37, 34, for intact flies, surgery flies, respectively. *p<0.05, two-tailed t-test. (**d**) A schematic of the experiment. Different subsets of body hair somatosensors are silenced by expressing TNT using indicated driver lines. (Upper) split GAL4-based experiments. (Lower) GAL-based experiments. (**e**) Change in HR, calculated for each fly as the % difference between the mean HR during the 10 s stimulation period and the HR during the 10 s baseline period preceding stimulation. N=36, 30, 27 for ‘empty’, ‘R38B08-AD R81E10-DBD’, ‘R38B08-AD VT043775-DBD’, respectively. *p<0.05, n.s.: p>0.05, Dunnett’s test. (**f**) Change in HR, calculated for each fly as the % difference between the mean HR during the 10 s stimulation period and the HR during the 10 s baseline period preceding stimulation. N=43, 23, 25, 26, 20, 20 for ‘+’, ‘R74C07’, ‘Aes1N’, ‘Tes1N’, ‘iav’, ‘R48A07’, respectively. ***p<0.001, *p<0.05, n.s.: p>0.05, Dunnett’s test. (**g**) Baseline HR. N=36, 30, 27 for ‘empty’, ‘R38B08AD R81E10-DBD’, ‘R38B08-AD VT043775-DBD’, respectively. ***p<0.001, **p<0.01, Dunnett’s test. (**h**) Baseline HR. N=43, 23, 25, 26, 20, 20 for ‘+’, ‘R74C07’, ‘Aes1N’, ‘Tes1N’, ‘iav’, ‘R48A07’, respectively. ***p<0.001, Dunnett’s test. (**i**) Somatosensory neurons suggested to be partially indispensable for puff-induced cardiac deceleration. When multiple drivers labeled the given neurons, we considered such neurons as indispensable for cardiac deceleration only when those drivers produced consistent results (hence, e.g. external sensilla on the posterior abdomen were excluded based on Aes1N driver’s result, despite R74C07 driver’s result suggesting otherwise; similarly, sensilla on the eye compound labeled by R74C07 driver were excluded by ‘R38B08-AD VT043775-DBD’ split combination’s result.).

**Video 1.** An air puff induces cardiac deceleration and promotes locomotion. A 50 mL/min puff is applied in the middle of the movie, as indicated by the red text that appears on the top left panel of the movie. The bottom left panel shows a behavioral recording, with automated body parts tracking (blue dots and lines) superimposed on the raw data. The top right panel shows the raw heartbeat recording, and the bottom right panel shows the pixel-wise, color-coded optical flow (color-to-direction mapping is shown on the top right of the panel). The playback speed is reduced to 0.25 of the original file.

Using our paradigm, we tested how the air puff might influence the HR and locomotion. Specifically, we applied for each fly a 0.5 s air puff (either 20 mL/min, 50 mL/min, 200 mL/min). We repeated 10 trials for each fly with an inter-puff interval of 90 s (Figure 1b). The results of 10 trials were averaged, as we did not observe noticeable trial effects (Figure 1—figure supplement 2a, c). We found that the air puff transiently decreased the HR (Figure 1c–e; Figure 1—figure supplement 2a, b; Video 1). A similar tendency was observed but did not reach statistical significance for the strongest puff (200 mL/min), which is likely due to the following cardiac acceleration (Figure 1d and e). Indeed, the beat-to-beat interval increased over the three beats following puff onset, and this increase was comparable across puff strengths (Figure 1—figure supplement 2e, f). Surgical removal of body parts and silencing experiments of external sensilla or chordotonal organs (Tsubouchi et al., 2017; Zhang et al., 2020) suggest that hair plate neurons and chordotonal organs are at least partially required for puff-induced cardiac deceleration (Figure 1—figure supplement 3). We also found that the air puff persistently increased the fraction of time walking, in line with previous reports (Tsuji et al., 2023; Lebestky et al., 2009; Figure 1f and g; Figure 1—figure supplement 2c, d). A close inspection of the peri-stimulus dynamics revealed that the onsets of the cardiac and locomotor changes were approximately concurrent at puff onset across strengths (Figure 1h). Furthermore, the peak of cardiac deceleration (roughly 0.2–0.5 s post puff onset) preceded that of locomotor increase (roughly 1–1.5 s post puff onset). Especially at lower puff strengths, locomotor response showed an additional transient dip, embedded in dominant and persistent locomotion, almost concurrently with (though, in the case of 50 mL/min, following) cardiac deceleration (Figure 1h). Overall, cardiac and locomotor responses begin together, and the cardiac response reaches its peak before the locomotor response does. These data are consistent with the idea that cardiac and locomotor responses are coordinated, and that cardiac deceleration may causally contribute to the locomotor response. We therefore set out to identify the neurons responsible for cardiac deceleration and to test their causal role directly.

### Dopaminergic system is responsible for puff-induced cardiac deceleration

**Figure 2.**
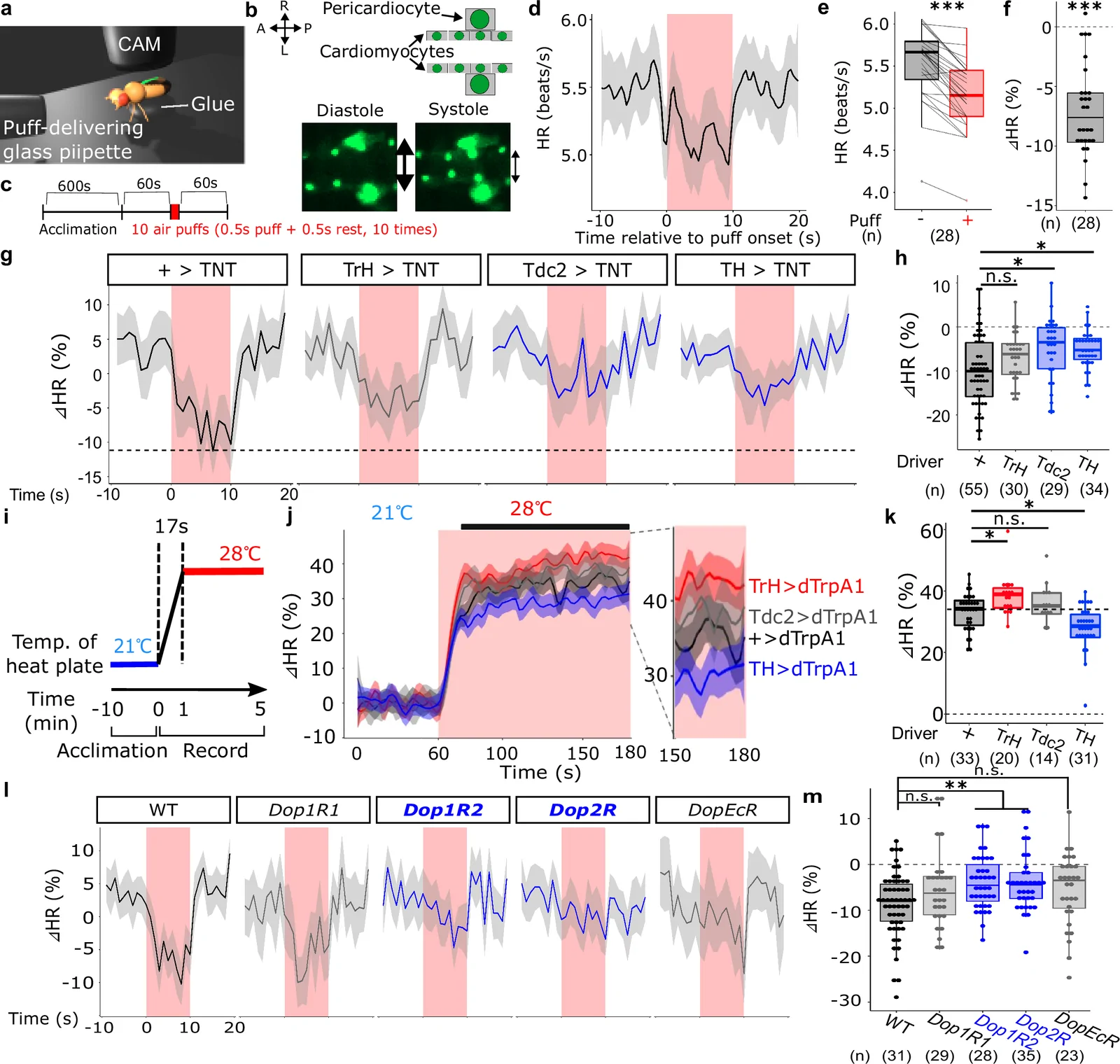
Dopamine system is responsible for puff-induced cardiac deceleration. (**a**) Experimental setup. Hand enhancer drives the GFP expression in the cardiomyocytes (and in pericardiocytes). The fly was glued onto a slide glass, and the GFP signals were acquired through the intact cuticle. The air puffs were applied frontally to the fly’s head. (**b**) Example heartbeat. (Upper) Cartoon of the acquired image. Two rows of small cardiomyocytes and one pair of large pericardiocytes are visible. (Lower) Diastole and systole can be distinguished by the changing distance between two rows of cardiomyocytes (arrows). (**c**) The time course of the experiment. Air puffs of 0.5 s were applied at 1 Hz for 10 times (totalling 10 s). The trial was repeated twice for each fly, the results of which were averaged. (**d**) Time course of the HR. Air puffs are applied during the red-shaded window. Lines and shaded areas represent means and SEM, respectively. N=28. (**e**) The HR averaged during the puff application window (‘puff +’) vs 10 s before puff application (‘puff -’). Each line corresponds to each fly. N=28, ***p<0.001, two-tailed t-test. (**f**) Difference between puff + HR and puff- HR, calculated for each fly. N=28, ***p<0.001, two-tailed t-test. (**g**) Time course of the HR (% change from baseline defined as the average during the 10 s preceding puff onset). Air puffs were applied during the red-shaded time window. Lines and shaded areas represent means and SEM, respectively. TNT expression is driven by indicated drivers. ‘+’ indicates driver-less control. Dashed line shows the lowest mean of ‘+’ control during puffs. N=55, 30, 29, 34, for +, TrH, Tdc2, TH, respectively. (**h**) Change in HR, calculated for each fly as the % difference between the mean HR during the 10 s stimulation period and the mean HR during the 10 s baseline period preceding stimulation. N=55, 30, 29, 34, for +, TrH, Tdc2, TH, respectively. *p<0.05, n.s.: p>0.05, Dunn’s test. (**i**) Time course of the experiment. The heat plate beneath the slide glass warmed up the fly. The temperature of the slide glass is held at 21°C for 10 min and ramped up to 28°C for 5 min. Note it took approximately 17 s for the temperature to ramp from 21 to 28°C. (**j**) Time course of the HR, shown as % change from average HR at 21°C. Temperature was ramped from 21 to 28°C during the red-shaded window. (**k**) % Changes in the HR (28 vs 21°C) from the baseline (average over initial 60 s) averaged over 75–180 s window (shown as black bar in j.). N=33, 20, 14, 31, for +, TrH, Tdc2, TH, respectively. *p<0.05, n.s.: p>0.05, Dunn’s test. The upper dashed line shows the median of ‘+’ control. (**l**) Time course of the HR (% change from baseline defined as the average during the 10 s preceding puff onset). Air puffs were applied during the red-shaded time window. Lines and shaded areas represent means and SEM, respectively. N=31, 29, 28, 35, 23, for +, WT, Dop1R1, Dop1R2, Dop2R, DopEcR, respectively. (**m**) Change in HR, calculated for each fly as the % difference between the mean HR during the 10 s stimulation period and the HR during the 10 s baseline period preceding stimulation. N=31, 29, 28, 35, 23, for +, WT, Dop1R1, Dop1R2, Dop2R, DopEcR, respectively. **p<0.01, n.s.: p>0.05, Dunn’s test.

To aid in identifying neurons responsible for cardiac deceleration, we developed yet another paradigm to quantify the HR with higher throughput. In this paradigm, we drove the expression of GFP in the cardiomyocytes using the enhancer of the Hand gene Han and Olson, 2005, to aid in heartbeat imaging (Figure 2a and b). GFP signals in pericardiocytes (also known as nephrocytes) are likely due to their function to incorporate any molecules in the hemolymph (Zhang et al., 2013). The fly’s ventral side of the thorax and abdomen were glued to a slide glass, and the GFP signals in the conical chamber were acquired through the intact dorsal cuticle by confocal microscopy (Figure 2a and b). The air puffs of 20 mL/min were applied frontally to the fly’s head through a glass pipette (Figure 2a). Heartbeat was detected using our custom software (Figure 2—figure supplement 1a–c). A single trial consisted of 60 s of interval, 10 s of air puffs, and 60 s of recording, which was repeated twice for each fly with a 90 s interval (Figure 2c). This experiment revealed that air puffs induce cardiac deceleration by roughly 10% (Figure 2d–f; Video 2), consistent with our earlier finding (Figure 1). Similar results were obtained when the air puff was applied for 1 s (Figure 2—figure supplement 1d, e), 20 s (Figure 2—figure supplement 1f, g), and in both sexes (Figure 2—figure supplement 1h, i).

**Figure 2—figure supplement 1.**
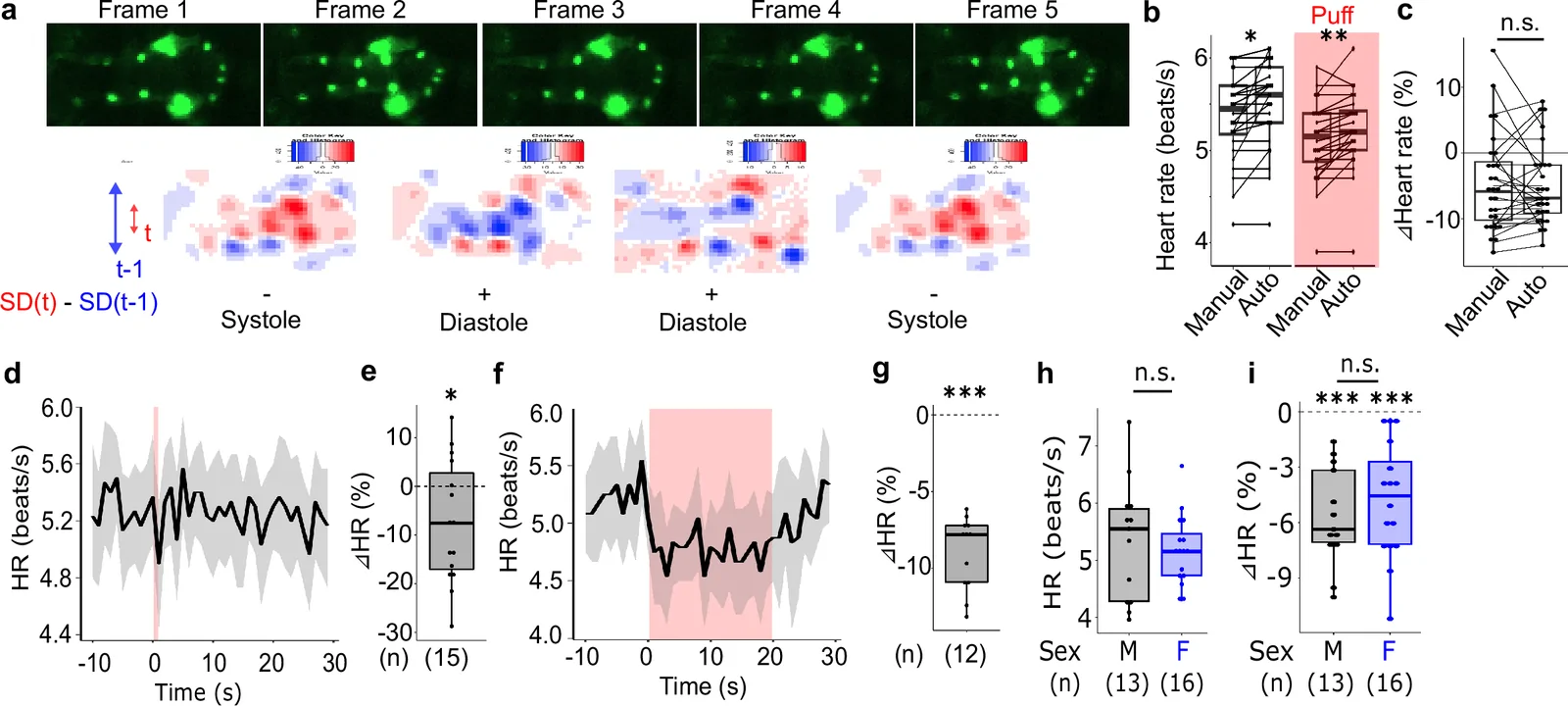
Heartbeat detection analysis. (**a**) (Upper) example frames capturing the heart tube of a Hand-GFP fly. (Lower) Heat plot showing for each pixel the difference between consecutive frames. Pixels whose value at time t is larger than that at time (t-1) are shown with red; blue for the opposite. Double-headed arrows show the width of the heart for time t and (t-1) for illustrative purposes, and are not meant to be quantitatively precise. ‘SD(t)’ indicates the standard deviation of the y coordinates of red pixels, and ‘SD(t-1)’ of blue pixels. SD(t) is larger when the width of the heart is larger. Thus, ‘SD(t) - SD(t-1)’ indicates systole when <0, and diastole when >0. (**b**) Plot comparing the HR calculated from manual count vs auto-detection, before and during puff application. Each line indicates identical flies. N=27. **p<0.01, *p<0.05, two-tailed, paired t-test followed by Bonferroni correction. (**c**) Plot comparing the % change in HR (during vs pre-puff, 10 s each) calculated from manual count vs auto-detection. n.s.: p>0.05, two-tailed t-test. (**d**) Time course of the HR. A single puff was applied during red-shaded time window. Lines and shaded areas represent means and SEM, respectively. N=15. (**e**) Change in HR, calculated for each fly as the % difference between the mean HR during the 1 s stimulation period and the HR during the 10 s baseline period preceding stimulation. N=15, *p<0.05, two-tailed t-test. (**f**) Time course of the HR. A train of 20 puffs was applied during red-shaded time window. Lines and shaded areas represent means and SEM, respectively. N=12. (**g**) Change in HR, calculated for each fly as the % difference between the mean HR during the 20 s stimulation period and the HR during the 10 s baseline period preceding stimulation. N=12, ***p<0.001, two-tailed t-test. (**h**) The baseline HR. N=13, 15 for male, female, respectively. n.s.: p>0.05, two-tailed t-test. (**i**) Change in HR, calculated for each fly as the % difference between the mean HR during the 10 s stimulation period and the HR during the 10 s baseline period preceding stimulation. N=13, 15 for male, female, respectively. ***p<0.001, n.s.: p>0.05, two-tailed t-test. Sex-wise statistics were Bonferroni-corrected.

**Figure 2—figure supplement 2.**
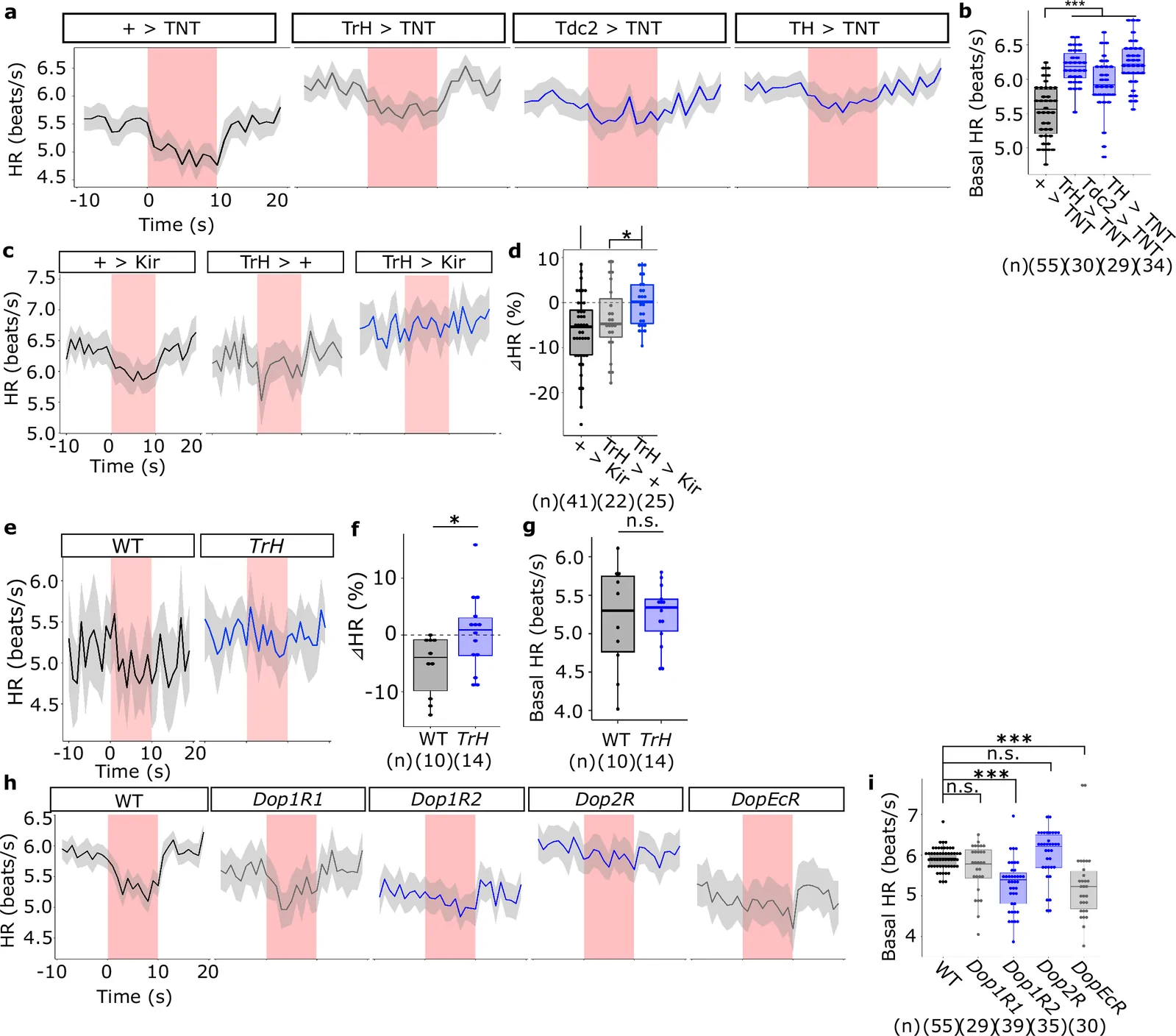
Roles of monoaminergic systems in puff-induced cardiac deceleration. (**a**) Time courses of the HR in beats per s. A train of 10 puffs was applied during the red-shaded time window. Lines and shaded areas represent means and SEM, respectively. N=55, 30, 29, 34, for ‘+’, TrH, Tdc2, TH, respectively. (**b**) The baseline HR in beats per s. N=55, 30, 29, 34, for ‘+’, TrH, Tdc2, TH, respectively. ***p<0.001, Dunnett’s test. (**c**) Time courses of the HR in beats per s. A train of 10 puffs was applied during the red-shaded time window. Lines and shaded areas represent means and SEM, respectively. N=41, 22, 25, for ‘+>Kir’, ‘TrH>+’, ‘TdH >Kir’, respectively. (**d**) Change in HR, calculated for each fly as the % difference between the mean HR during the 10 s stimulation period and the HR during the 10 s baseline period preceding stimulation. N=41, 22, 25, for ‘+>Kir’, ‘TrH>+’, ‘TdH >Kir’, respectively. ***p<0.001, *p<0.05, Dunnett’s test. (**e**) Time courses of the HR in beats per s. A train of 10 puffs was applied during the red-shaded time window. Lines and shaded areas represent means and SEM, respectively. N=10, 14 for WT, TrH, respectively. (**f**) Change in HR, calculated for each fly as the % difference between the mean HR during the 10 s stimulation period and the HR during the 10 s baseline period preceding stimulation. N=10, 14 for WT, TrH, respectively. *p<0.05, two-tailed t-test. (**g**) The baseline HR in beats per s. N=10, 14 for WT, TrH, respectively. n.s.: p>0.05, two-tailed t-test. (**h**) Time courses of the HR in beats per s. A train of 10 puffs was applied during the red-shaded time window. Lines and shaded areas represent means and SEM, respectively. N=55, 29, 39, 35, 30, for WT, Dop1R1, Dop1R2, Dop2R, DopEcR, respectively. (**i**) The baseline HR in beats per s. N=55, 29, 39, 35, 30, for WT, Dop1R1, Dop1R2, Dop2R, DopEcR, respectively. ***p<0.001, n.s.: p>0.05, Dunn’s test.

**Video 2.** A train of 10 air puffs induces cardiac deceleration in a Hand >GFP fly. The playback speed is reduced to 0.25 of the original file.

Monoaminergic systems have repeatedly been implicated in HR regulation across species, including *Drosophila* (Keller et al., 2004; Zornik et al., 1999; Zhu et al., 2016; Johnson et al., 1997; Hillyer, 2018; Majeed et al., 2014; Johnson et al., 2002; Schroeder and Jordan, 2012; Sujkowski et al., 2020; Schiemann et al., 2018). We thus wondered whether monoaminergic systems might be involved in puff-induced cardiac deceleration. When we silenced with TNT the neurons expressing either serotonin, octopamine (insect equivalent of norepinephrine), and dopamine, we found that silencing of octopaminergic or dopaminergic neurons significantly suppressed puff-induced cardiac deceleration (Figure 2g and h; Figure 2—figure supplement 2a, b). Note, however, that when we expressed in serotonergic neurons the inward-rectifying potassium channel Kir2.1, it did decrease the puff-induced cardiac deceleration (Figure 2—figure supplement 2c, d), suggesting that serotonergic neurons may also be indispensable for puff-induced cardiac deceleration. In line with this, a *loss-of-function* mutation of a serotonin synthetic enzyme *TrH* also showed a decrease in the puff-induced cardiac deceleration (Figure 2—figure supplement 2e–g).

To further probe the roles of monoaminergic neurons, we next tested whether thermogenetic activation of these neurons can induce cardiac deceleration without air puffs. To this end, we expressed a heat-gated cation channel dTrpA1 in monoaminergic neurons, and shifted the temperature from 21 to 28°C while monitoring the HR (Figure 2i). This revealed that, while the HR of cold-blooded *Drosophila* increased as a function of the temperature even in the driver-less control (Figure 2j, black), such cardiac acceleration was suppressed by activation of dopaminergic neurons (Figure 2j, blue) but not by octopaminergic neurons (Figure 2j, gray). On the other hand, activation of serotonergic neurons exaggerated the heat-induced cardiac acceleration (Figure 2j, red). The average HR during the 28°C period indeed revealed that the activation of dopaminergic neurons significantly decreased the HR compared to the driver-less control, while the activation of serotonergic neurons significantly increased the HR (Figure 2k). Dopaminergic neurons were the only monoaminergic population whose silencing and activation consistently implicated them in driving cardiac deceleration (Figure 2g–k). The serotonergic system, on the other hand, provided paradoxical results (Figure 2g–k, Figure 2—figure supplement 2c-g): this could be because the serotonergic system is required for threat-induced cardiac deceleration, while broad serotonergic activation outside this context possibly engages downstream circuits that promote cardiac acceleration. The role of dopaminergic neurons is further reinforced by *loss-of-function* experiments of dopamine receptors, which revealed that mutations of *Dop1R2* or *Dop2R* significantly decrease the puff-induced cardiac deceleration (Figure 2l and m; Figure 2—figure supplement 2h, i). A mutation of *DopEcR* also showed a tendency to decrease the puff-induced cardiac deceleration albeit not quite reaching statistical significance (Figure 2l and m; Figure 2—figure supplement 2h, i).

Together, our data demonstrate that, while not excluding the contributions of serotonergic or octopaminergic systems, the dopamine system is responsible for puff-induced cardiac deceleration.

### Two pairs of dopaminergic neurons in the brain, DA-WED neurons, are indispensable for cardiac deceleration

**Figure 3.**
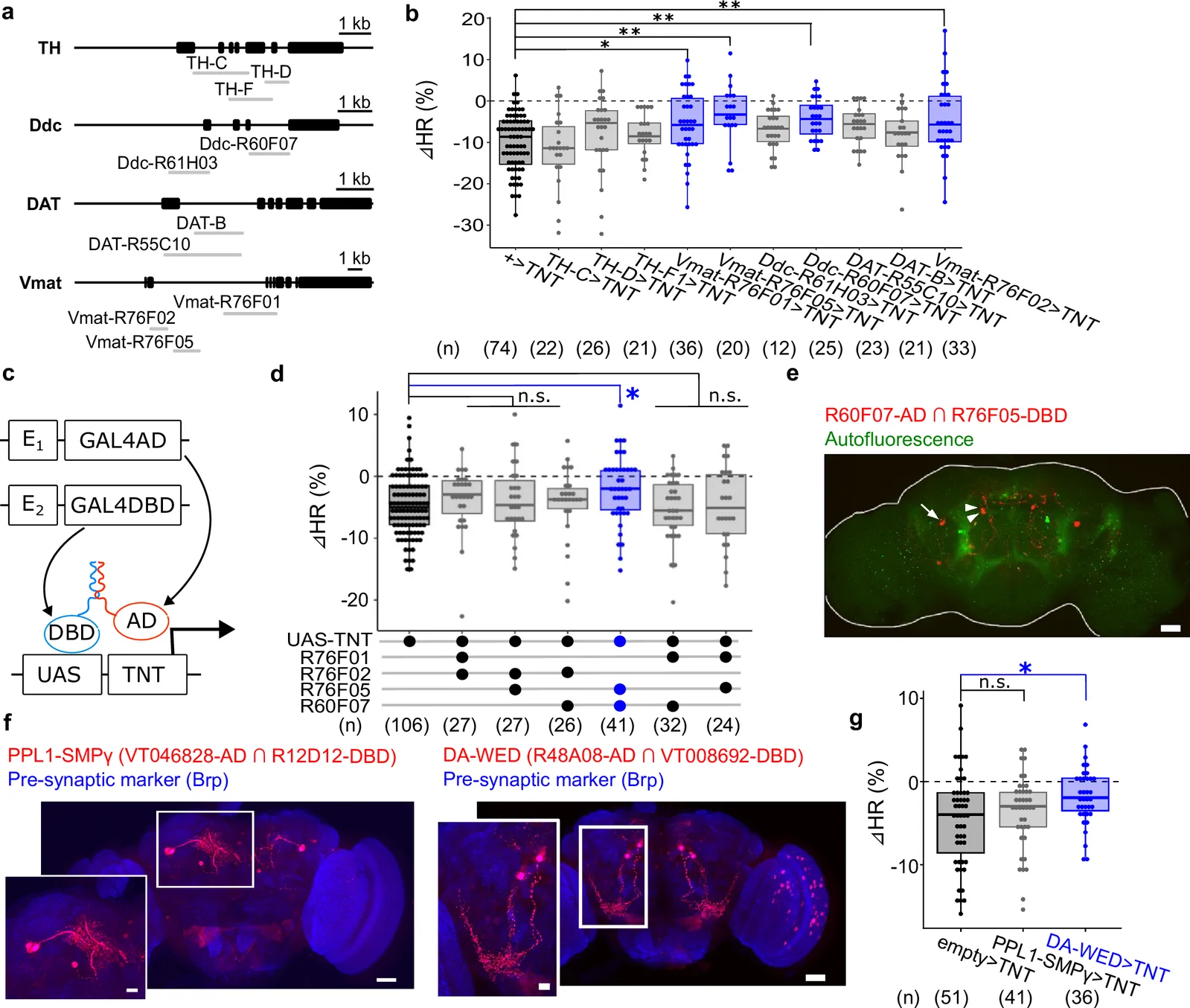
Two pairs of dopaminergic neurons in the brain are partially indispensable for cardiac deceleration. (**a**) Schematic of different DA-related GAL4 drivers. (**b**) Change in HR, calculated for each fly as the % difference between the mean HR during the 10 s stimulation period and the HR during the 10 s baseline period preceding stimulation. N=74, 22, 26, 21, 36, 20, 12, 25, 23, for +, THC, TH-D, TH-F1, Vmat-R76F01, Vmat-R76F05, Ddc-R61H03, Ddc-R60F07, DAT-R55C10, respectively. **p<0.01, *p<0.05, n.s. (data shown in gray): p>0.05, Dunnett’s test. (**c**) A schematic of split-GAL4 strategy. (**d**) Change in HR, calculated for each fly as the % difference between the mean HR during the 10 s stimulation period and the HR during the 10 s baseline period preceding stimulation. N=106, 27, 27, 26, 41, 32, 24, for driver-less control, R76F01 ∩ R76F02, R76F02 ∩ R76F05, R76F01 ∩ R76F02, R76F02 ∩ R76F07, R76F05 ∩ R60F07, R76F01 ∩ R60F07, R76F01 ∩ R76F05, respectively. *p<0.05, n.s.: p>0.05, Dunn’s test. (**e**) Brain of R60F07-AD ∩ R76F01-DBD>mCherry (red). An arrow indicates the soma of PPL1-SMPγ, while arrowheads indicate the soma of DA-WED. Scale bar: 25 µm. Similar results were obtained across three independent samples. (**f**) Brains of VT046828-AD ∩ R12D12-DBD>mCherry and R48A08-AD ∩ VT008692-DBD>mCherry (red) immunostained with antineuropil marker nc82 (blue). Scale bars: 25 µm (inlet: 5 µm). Similar results were obtained across five independent samples for each genotype. (**g**) Change in HR, calculated for each fly as the % difference between the mean HR during the 10 s stimulation period and the HR during the 10 s baseline period preceding stimulation. N=51, 41, 36 for empty >TNT, PPL1-SMPγ>TNT, DA-WED>TNT, respectively. *p<0.05, n.s.: p>0.05, Dunnett’s test.

There are approximately 130 dopaminergic neurons scattered throughout the *Drosophila* brain (Xie et al., 2018; Mao and Davis, 2009). To identify the subpopulation of dopaminergic neurons responsible for puff-induced cardiac deceleration, we turned to a host of dopamine-related GAL4 driver lines (Xie et al., 2018; Figure 3a). Each of these drivers carries different bits of enhancers of dopamine-related genes, and presumably for this reason these drivers are known to label partially overlapping but distinct subpopulations of dopaminergic neurons (Xie et al., 2018). When we drove the expression of TNT by these drivers, we found that neuronal silencing by 4 out of 10 drivers decreased puff-induced cardiac deceleration (R76F01-, R76F02-, R76F05-, R60F07-GAL4; Figure 3b, blue plots) without significantly influencing the baseline HR (Figure 3—figure supplement 1a), suggesting that these drivers label the subset of dopaminergic neurons indispensable for puff-induced cardiac deceleration. Identification of these ‘hit’ drivers allowed us to next employ a split-GAL4 approach to further narrow down the indispensable neurons (Figure 3c). In this approach, GAL4 is divided into DBD and AD hemi-drivers, and functional GAL4 is reconstituted only in cells expressing both components (Luan et al., 2006; Figure 3c). When we tested all split combinations of the four ‘hit’ enhancers, we found that only one combination, namely R76F05-DBD and R60F07-AD, significantly decreased the puff-induced cardiac deceleration (Figure 3d), without significantly influencing the baseline HR (Figure 3—figure supplement 1b). Our immunohistochemical analysis revealed that, as reported by a previous report (Xie et al., 2018), the split combination of R76F05-DBD and R60F07-AD labeled two different subpopulations of dopaminergic neurons: one subpopulation from the PPL1 cluster named PPL1-SMPγ, and another subpopulation from the PPM2 cluster named DA-WED (Figure 3e), with additional one or two neurons occasionally labeled in some brains. We further sought to identify which of these two subpopulations is indispensable for puff-induced cardiac deceleration. Taking advantage of the recently developed analysis pipeline that identifies a list of candidate GAL4 drivers that may label any neuron of interest (Clements et al., 2024; Mais et al., 2021), we obtained a list of candidate PPL1-SMPγ and DA-WED drivers. By testing several split combinations of enhancers used in these drivers, we could identify the split combinations that label one but not the other (Figure 3f). We note that each of these combinations labels additional neurons, in either subesophageal zone (Figure 3f, left) or optic lobes (Figure 3f, right). Using these split drivers, we silenced either PPL1-SMPγ or DA-WED with TNT, and found that silencing of DA-WED significantly decreased puff-induced cardiac deceleration (Figure 3g; Figure 3—figure supplement 1c). Together, our data suggests that DA-WED neurons are indispensable for puff-induced cardiac deceleration.

**Figure 3—figure supplement 1.**
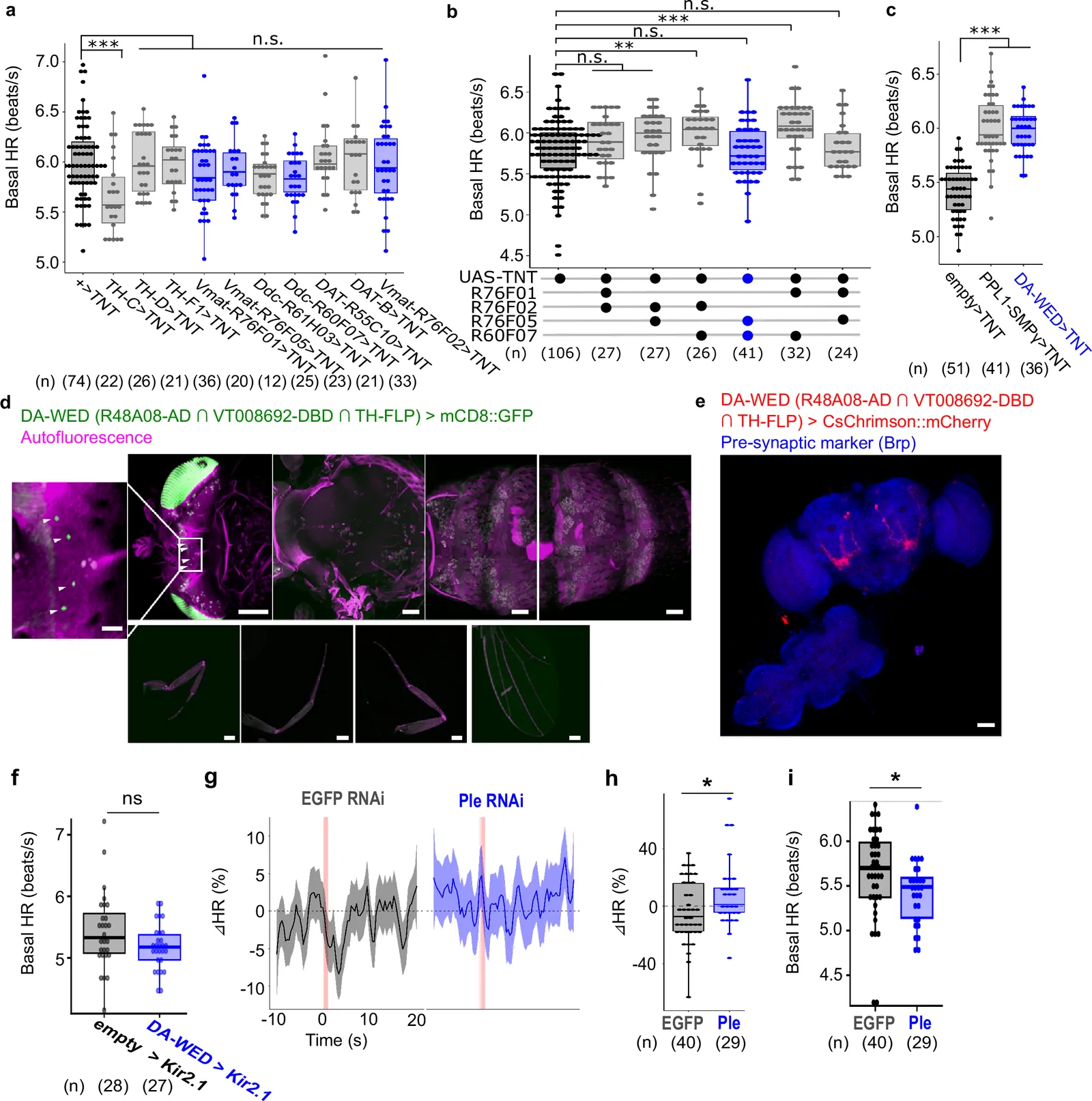
Silencing different subsets of dopaminergic neurons, DA-WED driver’s expression pattern, DA-WED silencing, and ple RNAi. (**a**) The baseline HR in beats per s. N=74, 22, 26, 21, 36, 20, 12, 25, 23, 21, 33, for ‘+’, TH-C, TH-D, TH-F1, Vmat-R76F01, Vmat-R76F05, Ddc-R61H03, Ddc-R60F07, DAT-R55C10, DAT-B, Vmat-R76F02, respectively. ***p<0.05, n.s.: p>0.05, Dunn’s test. (**b**) The baseline HR in beats per s. N=106, 27, 27, 26, 41, 32, 24, for driver-less control, R76F01 ∩ R76F02, R76F02 ∩ R76F05, R76F01 ∩ R76F02, R76F02 ∩ R76F07, R76F05 ∩ R60F07, R76F01 ∩ R60F07, R76F01 ∩ R76F05, respectively. ***p<0.001, **p<0.01, n.s.: p>0.05, Dunn’s test. (**c**) The baseline HR in beats per s. N=51, 41, 36, for empty, PPL1-SMPγ, DA-WED, respectively. ***p<0.001, Dunn’s test. (**d**) Cleared tissue of a fly expressing mCD8GFP in DA-WED neurons R48A08-AD ∩ VT008692-DBD ∩ TH-FLP>mCD8::GFP (green). Autofluorescence of the tissue is visible as magenta. Arrowheads indicate DA-WED neuron’s soma. The bottom panels each show, from left to right, a foreleg, midleg, hindleg, and wing. Scale bar: 100 µm (inlet: 25 µm). (**e**) A brain and VNC of R48A08-AD ∩ VT008692-DBD ∩ TH-FLP>CsChrimson::mCherry (red) immunostained with anti-neuropil marker nc82 (blue). Scale bars: 25 µm (inlet: 5 um). Similar results were obtained across seven independent samples. Scale bar: 50 µm. (**f**) The baseline HR in beats per s. N=28, 27, for empty >Kir2.1, DA-WED>Kir2.1, respectively. n.s.: p>0.05, two-tailed t-test. (**g**) Time course of the HR (% change from baseline defined as the average during the 10 s preceding puff onset). An air puff was applied during the red-shaded time window. Lines and shaded areas represent means and SEM, respectively. N=40, 29, for EGFP-RNAi, ple-RNAi, respectively. (**h**) Change in HR, calculated for each fly as the % difference between the mean HR during the 1 s following stimulation onset and the mean HR during the 10 s baseline period preceding stimulation. N=40, 29, for EGFP-RNAi, ple-RNAi, respectively. *p<0.05, two-tailed t-test. (**i**) The baseline HR. N=40, 29, for EGFP-RNAi, Ple-RNAi, respectively. *p<0.05, Wilcoxon’s signed rank test.

### DA-WED neurons are responsible for puff-induced cardiac deceleration

**Figure 4.**
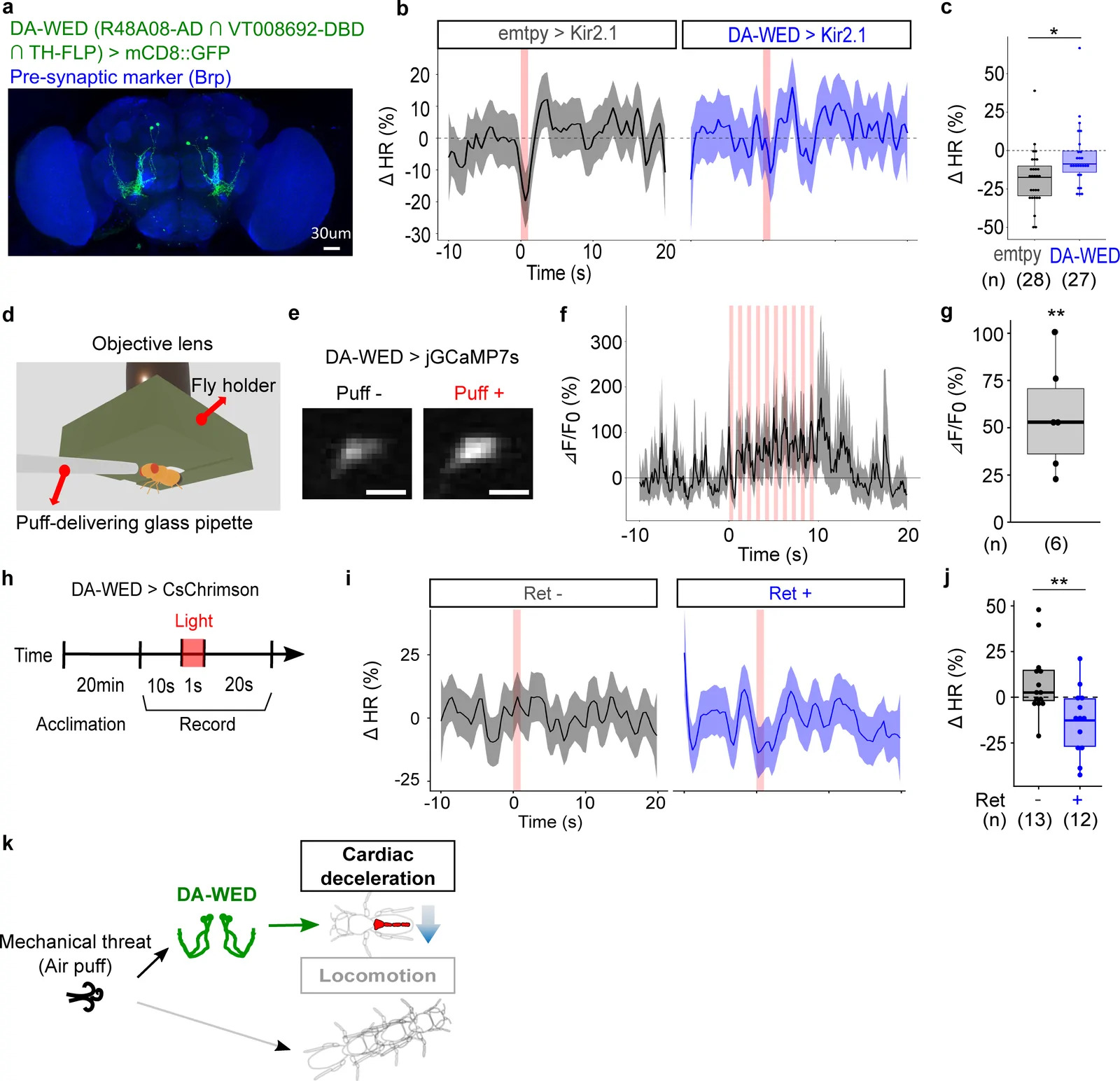
DA-WED neurons are responsible for puff-induced cardiac deceleration. (**a**) The brain of R48A08-AD ∩ VT008692-DBD ∩ TH-FLP>mCD8::GFP (green) immunostained with anti-neuropil marker nc82 (blue). Scale bar: 30 um. Similar results were obtained across five independent samples. (**b**) Time course of the HR (% change from baseline defined as the average during the 10 s preceding puff onset). Air puff was applied during the red-shaded time window. Lines and shaded areas represent means and SEM, respectively. N=28, 27, for empty >Kir2.1, DA-WED>Kir2.1, respectively. (**c**) Change in HR, calculated for each fly as the % difference between the mean HR during the 1 s following stimulation onset and the mean HR during the 10 s baseline period preceding stimulation. N=28, 27, for empty >Kir2.1, DA-WED>Kir2.1, respectively. *p<0.05, Two-tailed t-test. (**d**) Experimental setup. (**e**) Representative images of DA-WED neurons expressing GCaMP7s before and during puff application. Scale bar: 5 μm. (**f**) Time course of ΔF/F0. Puffs were applied at 1 Hz for 10 times (over 10 s), as indicated by red-shaded boxes. N=6 flies. (**g**) ΔF/F0 averaged over 10 s of puff application. When multiple neurons were recorded from the same fly (max 4 neurons per fly), results were averaged so that each dot represents one fly. N=6 flies. two-tailed t-test. **p<0.01. (**h**) Time course of the experiment. After 20 min of acclimation, the heartbeat of the fly expressing light-gated cation channel CsChrimson in DA-WED neurons, with (‘Ret +’) or without (‘Ret -’) being fed with the co-factor all-trans retinal (ATR) for 2 days prior to experiment, was recorded for 10 s, followed by 1 s of light application, and 20 s of recording. (**i**) Time course of the HR (% change from baseline defined as the average during the 10 s preceding puff onset). Red light was shined during the red-shaded time window. Lines and shaded areas represent means and SEM, respectively. N=19, 22, for Ret-, Ret+, respectively. (**j**) Change in HR, calculated for each fly as the % difference between the mean HR during the 1 s following stimulation onset and the mean HR during the 10 s baseline period preceding stimulation. N=13, 12, for Ret-, Ret+, respectively. *p<0.05, two-tailed t-test. (**k**) Model.

While our data so far suggests the role of DA-WED neurons in cardiac deceleration, the split driver we used to label DA-WED also labeled neurons in the optic lobes (Figure 3f; right). We thus further improved the labeling specificity through intersection strategy. In this strategy, the effector gene is expressed under the control of UAS sequence, but a ‘stop’ sequence is inserted in between UAS and GFP, blocking the function of GAL4. Now, the expression of ‘molecular scissors’ called flippase (FLP) is driven by a Tyrosine hydroxylase (TH) promoter (‘TH-FLP’), which excises out the stop sequence. Hence, GAL4 can function only in cells in which TH promoter is active. This strategy, combined with our split GAL4 combination, indeed achieved near-perfect specificity for DA-WED neurons, apparently labeling no other cells throughout the body (Figure 4a; Figure 3—figure supplement 1d, e). When we silenced DA-WED neurons with Kir2.1, it decreased puff-induced cardiac deceleration (Figure 4b and c), without noticeably influencing the baseline HR (Figure 3—figure supplement 1f).

**Figure 4—figure supplement 1.**
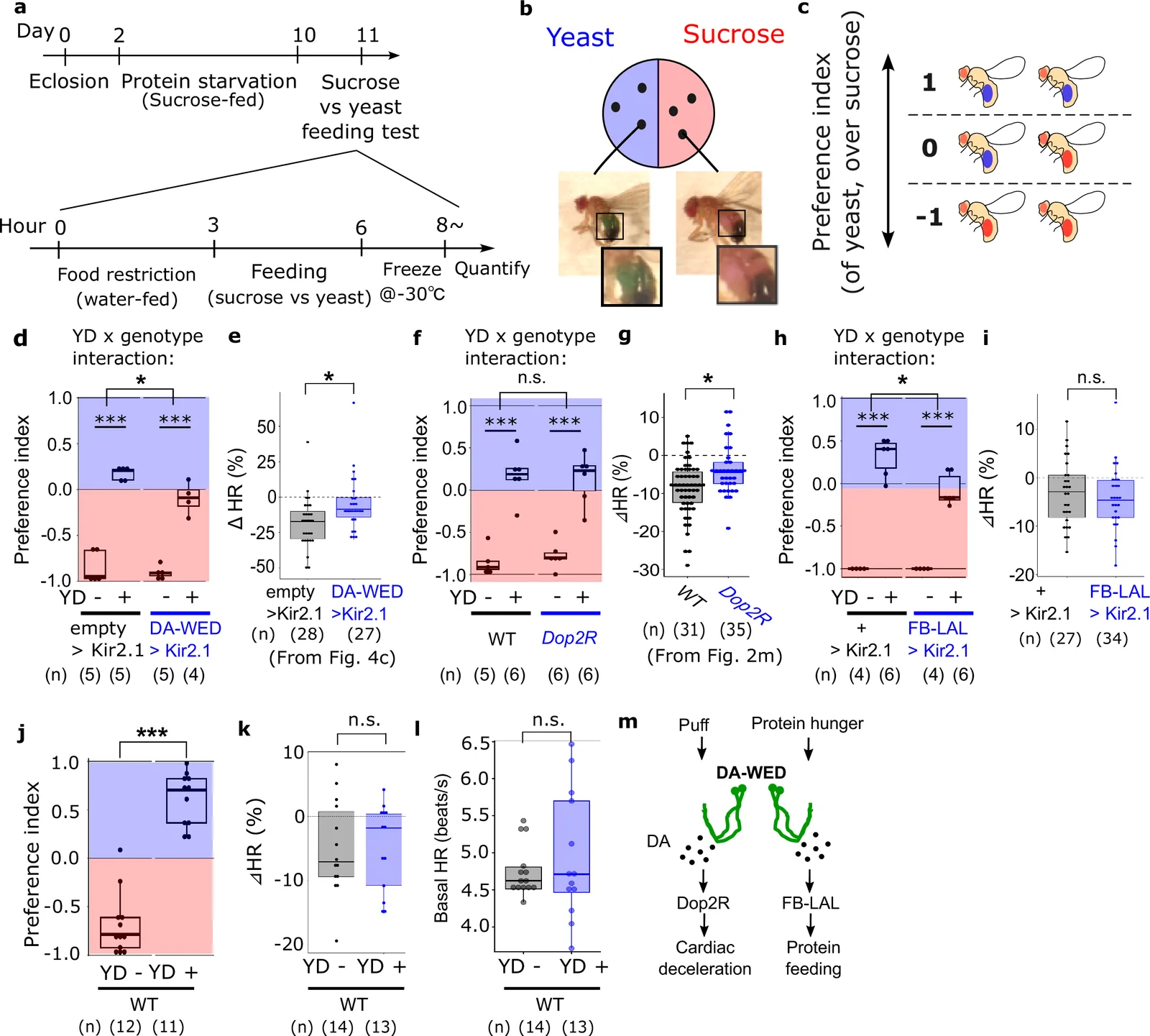
DA-WED neurons induce cardiac deceleration and protein feeding through distinct mechanisms. (**a**) Time course of the experiment. Flies 2 days post eclosion were subjected to 8-day protein starvation protocol. Protein preference of the starved flies was then tested the next day. The preference test consisted of 3 hr food restriction, then 3 hr feeding on sucrose (supplemented with red dye) or yeast (supplemented with blue dye). The flies were then frozen and their abdominal color was inspected. (**b**) Protein preference test. Flies were transferred into a 35 mm petri dish, one half of which contained agarose gel with yeast + blue dye, while the other half contained sucrose + red dye. (**c**) Preference index was calculated as (# flies consuming yeast - # flies consuming sucrose) / (total # flies that fed). (**d**) Preference index. N=5, 5, 5, 4 batches (each with ~60 flies) for empty YD-, empty YD+, DA-WED YD-, DA-WED YD+, respectively. ***p<0.001, *p<0.05, two-tailed t-test. YD- vs YD +statistics were Bonferroni-corrected. (**e**) Change in HR, calculated for each fly as the % difference between the mean HR during the 1 s following stimulation onset and the mean HR during the 10 s baseline period preceding stimulation. N=28, 27, for empty >Kir2.1, DA-WED>Kir2.1, respectively. *p<0.05, Two-tailed t-test. Figure 4c is shown here again for comparison with (**d**) (**f**) Preference index. N=5, 6, 6, 6 batches for WT YD-, WT YD+, Dop2R YD-, Dop2R YD+, respectively. *p<0.05, two-tailed t-test. YD- vs YD + statistics were Bonferroni-corrected. (**g**) Change in HR, calculated for each fly as the % difference between the mean HR during the 10 s stimulation period and the HR during the 10 s baseline period preceding stimulation. N=31, 35, for WT, Dop2R, respectively. Figure 2m is shown here again for comparison with (**f**) *p<0.05, Dunn’s test. (**h**) Preference index. N=4, 6, 4, 6 batches for empty YD-, empty YD+, FB-LAL YD-, FB-LAL YD+, respectively. ***p<0.001, *p<0.05, two-tailed t-test. YD- vs YD + statistics were Bonferroni-corrected. (**i**) Change in HR, calculated for each fly as the % difference between the mean HR during the 1 s following stimulation onset and the mean HR during the 10 s baseline period preceding stimulation. N=27, 34, for empty >Kir2.1, FB-LAL>Kir2.1, respectively. n.s.: p>0.05, two-tailed t-test. (**j**) Preference index. N=12, 11 batches for YD-, YD+, respectively. ***p<0.001, two-tailed t-test. (**k**) Change in HR, calculated for each fly as the % difference between the mean HR during the 1 s following stimulation onset and the mean HR during the 10 s baseline period preceding stimulation. N=14, 13, for YD-, YD+, respectively. n.s.: p>0.05, two-tailed t-test. (**l**) Baseline HR in beats per s. N=14, 13, for YD-, YD+, respectively. n.s.: p>0.05, Wilcoxon’s rank sum test. (**m**) Model.

**Figure 4—figure supplement 2.**
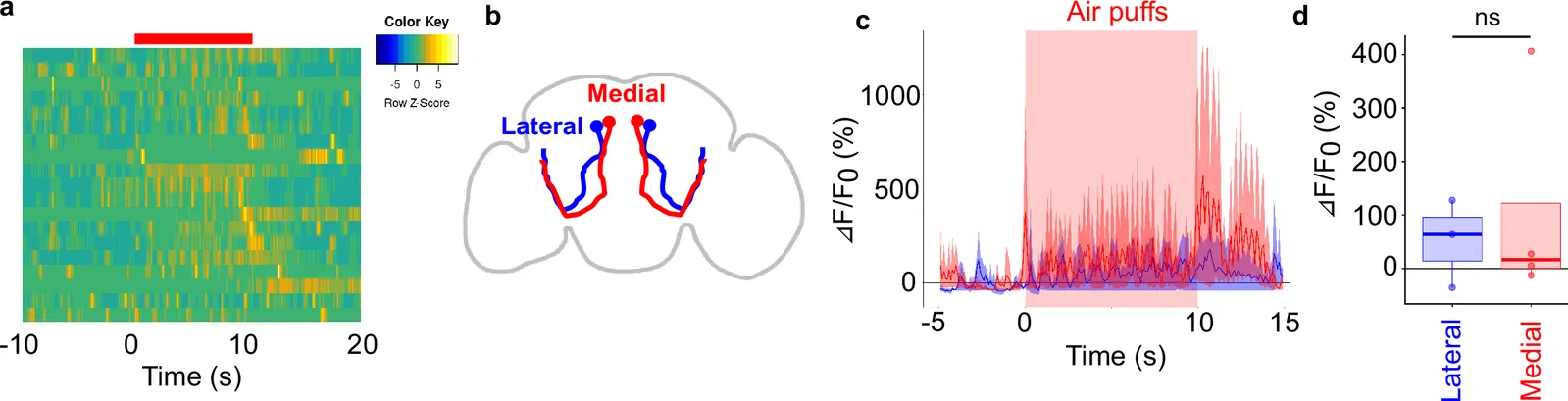
Response of DA-WED neurons to air puffs. (**a**) Rows depict the color-coded activity of a cell (z score). Red bar indicates the time window during which air puffs were applied. N=19 cells from 6 flies. (**b**) A cartoon showing medial and lateral DA-WED neurons. (**c**) Time course of ΔF/F0. Puffs were applied at 1 Hz for 10 times (over 10 s). (**d**) ΔF/F0 averaged over 10 s of puff application. When multiple neurons were recorded from the same fly (max 2 neurons per fly), results were averaged so that each dot represents one fly. Neurons for which medial/lateral location could not be distinguished are excluded from this analysis. N=3, 4 neurons for lateral and medial, respectively. Two-tailed t-test. n.s.: p>0.05.

We next tested if DA-WED neurons function through dopamine. Our dopamine receptor mutant analysis (Figure 2l and m) suggests that dopamine signaling through Dop1R2 and Dop2R is indispensable for cardiac deceleration. Consistent with this, we found that RNAi of a dopamine synthesis enzyme ple in DA-WED neurons reduced puff-induced cardiac deceleration (Figure 3—figure supplement 1g–i). It is thus plausible that DA released from DA-WED neurons binds to Dop1R2 and/or Dop2R to drive cardiac deceleration.

DA-WED neurons have previously been reported to drive protein preference behavior in protein-deprived flies (Wu et al., 2024; Liu et al., 2017). Indeed, suppression of DA-WED neurons decreased protein preference (Figure 4—figure supplement 1a–d; Wu et al., 2024; Liu et al., 2017) in addition to cardiac deceleration (Figure 4—figure supplement 1e, Figure 4b and c). This prompted us to ask whether these neurons drive cardiac deceleration and protein preference through shared, or instead distinct, mechanisms. Interestingly, *Dop2R* mutant flies failed to show cardiac deceleration (Figure 2l and m), but showed normal preference compared to the wildtype (Figure 4—figure supplement 1f, g). Similarly, suppression of FB-LAL neurons, through which DA-WED neurons drive protein preference (Wu et al., 2024; Liu et al., 2017), failed to influence cardiac deceleration (Figure 4—figure supplement 1h, i). These results suggest that DA-WED neurons drive protein preference through FB-LAL neurons, while driving cardiac deceleration through Dop2R independent of FB-LAL. This hypothesis is further reinforced by our observations that protein deprivation, which drives protein preference (Figure 4—figure supplement 1j), fails to influence puff-induced cardiac deceleration (Figure 4—figure supplement 1k) nor the baseline HR (Figure 4—figure supplement 1l). Together, our data suggests that DA-WED neurons drive cardiac deceleration and protein preference through distinct mechanisms (Figure 4—figure supplement 1m).

We next wondered how DA-WED neurons might respond to air puffs. To this end, we expressed jGCaMP7s in DA-WED neurons, and imaged their calcium activities while applying a train of air puffs (Figure 4d). We found that the calcium level of DA-WED neurons steadily increased as air puffs are applied (Figure 4e and f; Figure 4—figure supplement 2a; Video 3). The increased jGCaMP7s signals returned to the baseline in less than 5 s when puffs ceased (Figure 4f). Given the slow kinetics of jGCaMP7s (Dana et al., 2019), the increased activity of DA-WED neurons likely returns to the baseline immediately when the puffs cease. While there are two pairs of DA-WED neurons in the brain, one pair being more medial than the other, we found no noticeable difference in medial vs lateral DA-WED neurons’ activities in response to puffs (Figure 4—figure supplement 2b–d). We note, though, that the medial neurons tended to show offset response-like activities more pronounced than their lateral counterparts (Figure 4—figure supplement 2c), potentially hinting at functional heterogeneity within DA-WED neurons. Overall, our data suggests that all four DA-WED neurons transiently get activated by air puffs.

**Video 3.** Air puffs activate DA-WED neurons. The left panel shows a schematic of the DA-WED neurons and the actual calcium imaging data (averaged over the total recording), capturing both DA-WED neurons in the right hemisphere. An arrow indicates the neuron shown on the right panel. The right panel shows GCaMP signals at time 0 on the top, and the time-lapse imaging from there during which air puffs were applied.

To test if increased activities of DA-WED neurons can replace the air puff to induce cardiac deceleration, we photoactivated DA-WED neurons without puff application (Figure 4h). For this, we expressed a light-gated cation channel CsChrimson in DA-WED neurons, and shined light on the fly’s head to activate these neurons. We found that the photoactivation of DA-WED neurons could induce cardiac deceleration by ~10% in the retinal-fed test flies, but not in the retinal-unfed control flies (Figure 4i and j; Video 3).

Together, our data establishes that DA-WED neurons are responsible for puff-induced cardiac deceleration (Figure 4k).

### Cardiac interoception possibly contributes to threat-induced locomotion

**Figure 5.**
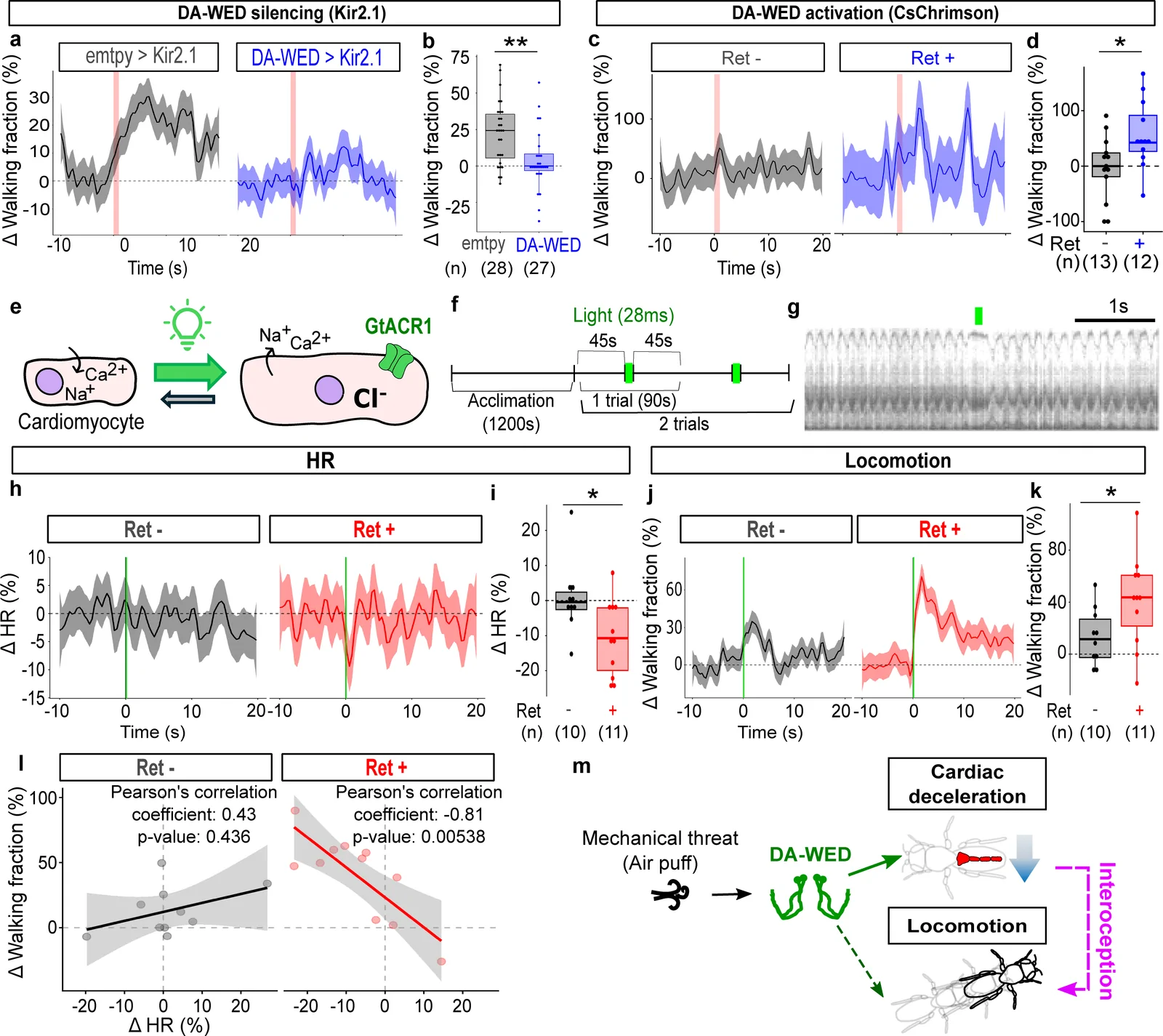
Cardiac interoception possibly contributes to threat-induced locomotion. (**a**) Time course of the walking fraction (% change from baseline defined as the mean fraction during the 10 s preceding puff onset). An air puff was applied during the red-shaded time window. Lines and shaded areas represent means and SEM, respectively. N=28, 27, for empty >Kir2.1, DAWED >Kir2.1, respectively. (**b**) Change in walking fraction, calculated for each fly as the % difference between the mean fraction during the 10 s following stimulation onset and the mean fraction during the 10 s baseline period preceding stimulation. N=28, 27, for empty >Kir2.1, DAWED >Kir2.1, respectively. **p<0.01, Two-tailed t-test. (**c**) Time course of the walking fraction (% change from baseline defined as the mean fraction during the 10 s preceding puff onset). Red light was applied during the red-shaded time window. Lines and shaded areas represent means and SEM, respectively. N=13, 12, for ret -, +, respectively. (**d**) Change in walking fraction, calculated for each fly as the % difference between the mean fraction during the 10 s following stimulation onset and the mean fraction during the 10 s baseline period preceding stimulation. N=13, 12, for ret -, +, respectively. **p<0.01, Two-tailed t-test. (**e**) A schematic of the strategy. Cardiomyocytes beat by oscillating between shrunk (systole) and relaxed (diastole) states. Diastole is driven by efflux of cation and resultant hyperpolarization. We expressed a light-gated anion channel GtACR1 in cardiomyocytes using Hand-GAL4, thus artificially driving hyperpolarization by shining light. (**f**) Time course of the experiment. After 20 min of acclimation, the heartbeat of the fly expressing GtACR1 in cardiomyocytes, with or without being fed with the co-factor all-trans retinal (ATR) for 2 days prior to experiment, was recorded for 90 s, followed by a 28ms light pulse, and 45 s of recording. The experiment was repeated twice for each fly. (**g**) An example M-mode during optogenetic cardiac deceleration. Green bar indicates when the light was shined. (**h**) Time course of the HR (% change from baseline defined as the average during the 10 s preceding puff onset). Green light was shined during the green-shaded time window. Lines and shaded areas represent means and SEM, respectively. N=10, 11, for ret -, ret +, respectively. (**i**) Change in HR, calculated for each fly as the % difference between the mean HR during the 1 s following stimulation onset and the mean HR during the 10 s baseline period preceding stimulation. N=10, 11, for ret -, ret +, respectively. *p<0.05, two-tailed t-test. (**j**) Time course of the walking fraction (% change from baseline defined as the mean fraction during the 10 s preceding puff onset). Light was shined during the green-shaded time window. Lines and shaded areas represent means and SEM, respectively. N=10, 11, for ret -, ret +, respectively. (**k**) Change in walking fraction, calculated for each fly as the % difference between the mean fraction during the 10 s following stimulation onset and the mean fraction during the 10 s baseline period preceding stimulation. N=10, 11, for ret -, ret +, respectively. *p<0.05, two-tailed t-test. (**l**) Correlations between changes in walking fraction and HR. N=10, 11, for ret -, ret +, respectively. (**m**) Model. DA-WED neurons promote locomotion, through and/or bypassing interoception of the heartbeat (dashed arrows).

Cardiac interoception has recently been implicated in modulating behavioral responses (Klein et al., 2021; Hsueh et al., 2023; Yao et al., 2022). We therefore asked whether cardiac deceleration is associated with changes in locomotion in *Drosophila*. To test this, we silenced DA-WED neurons using Kir2.1 to block puff-induced cardiac deceleration, and assessed locomotor output. We found that silencing DA-WED neurons markedly suppressed puff-induced locomotion compared to controls (Figure 5a and b). Conversely, photoactivation of DA-WED neurons using CsChrimson promoted locomotion in the absence of puff (Figure 5c and d, Video 4). These findings suggest that DA-WED neurons coordinate cardiac deceleration with locomotor responses upon mechanical threat.

**Figure 5—figure supplement 1.**
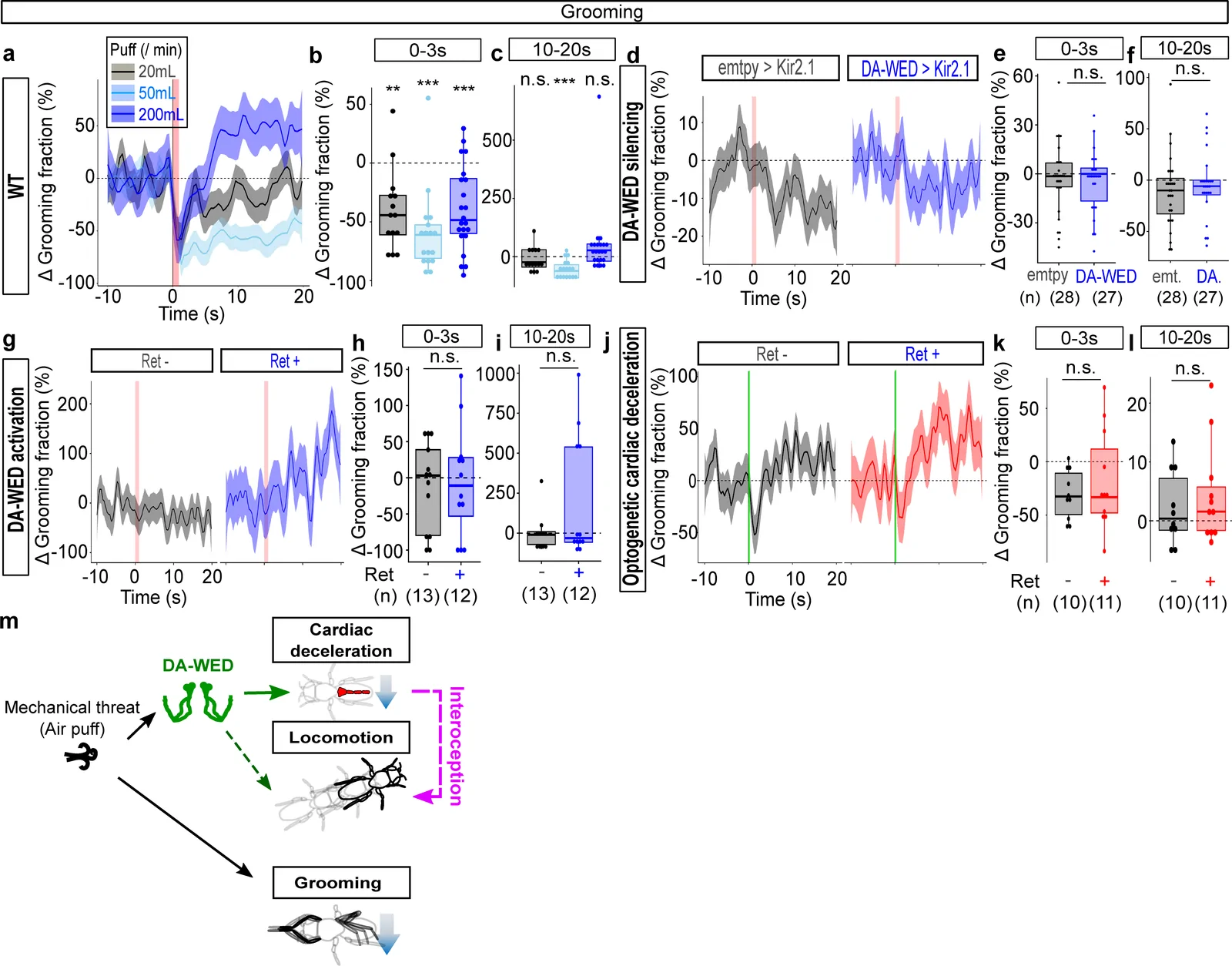
The effects of air puff, DA-WED manipulation, and optogenetic cardiac deceleration on grooming behavior. (**a**) Time course of the grooming fraction (% change from baseline defined as the mean fraction during the 10 s preceding puff onset). An air puff was applied during the red-shaded time window. Lines and shaded areas represent means and SEM, respectively. N=14, 17, 22 for 20 mL/min (gray), 50 mL/min (sky blue), and 200 mL/min (dark blue), respectively. (**b, c**) Change in grooming fraction, calculated for each fly as the % difference between the mean fraction during the 3 s (**b**) or 10–20 s (**c**) following stimulation onset and the mean fraction during the 10 s baseline period preceding stimulation. N=14, 17, 22 for 20 mL/min (gray), 50 mL/min (sky blue), and 200 mL/min (dark blue), respectively. ***p<0.001, **p<0.01, Wilcoxon’s signed rank test followed by Bonferroni correction. (**d**) Time course of the grooming fraction (% change from baseline defined as the mean fraction during the 10 s preceding puff onset). An air puff was applied during the red-shaded time window. Lines and shaded areas represent means and SEM, respectively. N=28, 27, for empty >Kir2.1, DA-WED>Kir2.1, respectively. (**e, f**) Change in grooming fraction, calculated for each fly as the % difference between the mean fraction during the 3 s (**e**) or 10–20 s (**f**) following stimulation onset and the mean fraction during the 10 s baseline period preceding stimulation. N=28, 27, for empty >Kir2.1, DA-WED>Kir2.1, respectively. n.s.: p>0.05, Two-tailed t-test. (**g**) Time course of the grooming fraction (% change from baseline defined as the mean fraction during the 10 s preceding puff onset). Light was shined during the red-shaded time window. Lines and shaded areas represent means and SEM, respectively. N=13, 12, for ret -, ret +, respectively. (**h, i**) Change in grooming fraction, calculated for each fly as the % difference between the mean fraction during the 3 s (**h**) or 10–20 s (**i**) following stimulation onset and the mean fraction during the 10 s baseline period preceding stimulation. N=13, 12, for ret -, ret +, respectively. n.s.: p>0.05, Two-tailed t-test. (**j**) Time course of the grooming fraction (% change from baseline defined as the mean fraction during the 10 s preceding puff onset). Light was shined during the green-shaded time window. Lines and shaded areas represent means and SEM, respectively. N=10, 11, for ret -, ret +, respectively. (**k, l**) Change in grooming fraction, calculated for each fly as the % difference between the mean fraction during the 3 s (**k**) or 10–20 s (**l**) following stimulation onset and the mean fraction during the 10 s baseline period preceding stimulation. N=10, 11, for ret -, ret +, respectively. n.s.: p>0.05, two-tailed t-test. (**m**) Model. Air puff promotes locomotion while suppressing grooming. Mechanistically, air puff activates DA-WED neurons. DA-WED neurons drive not only cardiac deceleration but locomotion, potentially through a feedback from cardiac deceleration (dashed arrows). Such a DA-WED-based mechanism fails to influence grooming.

**Figure 5—figure supplement 2.**
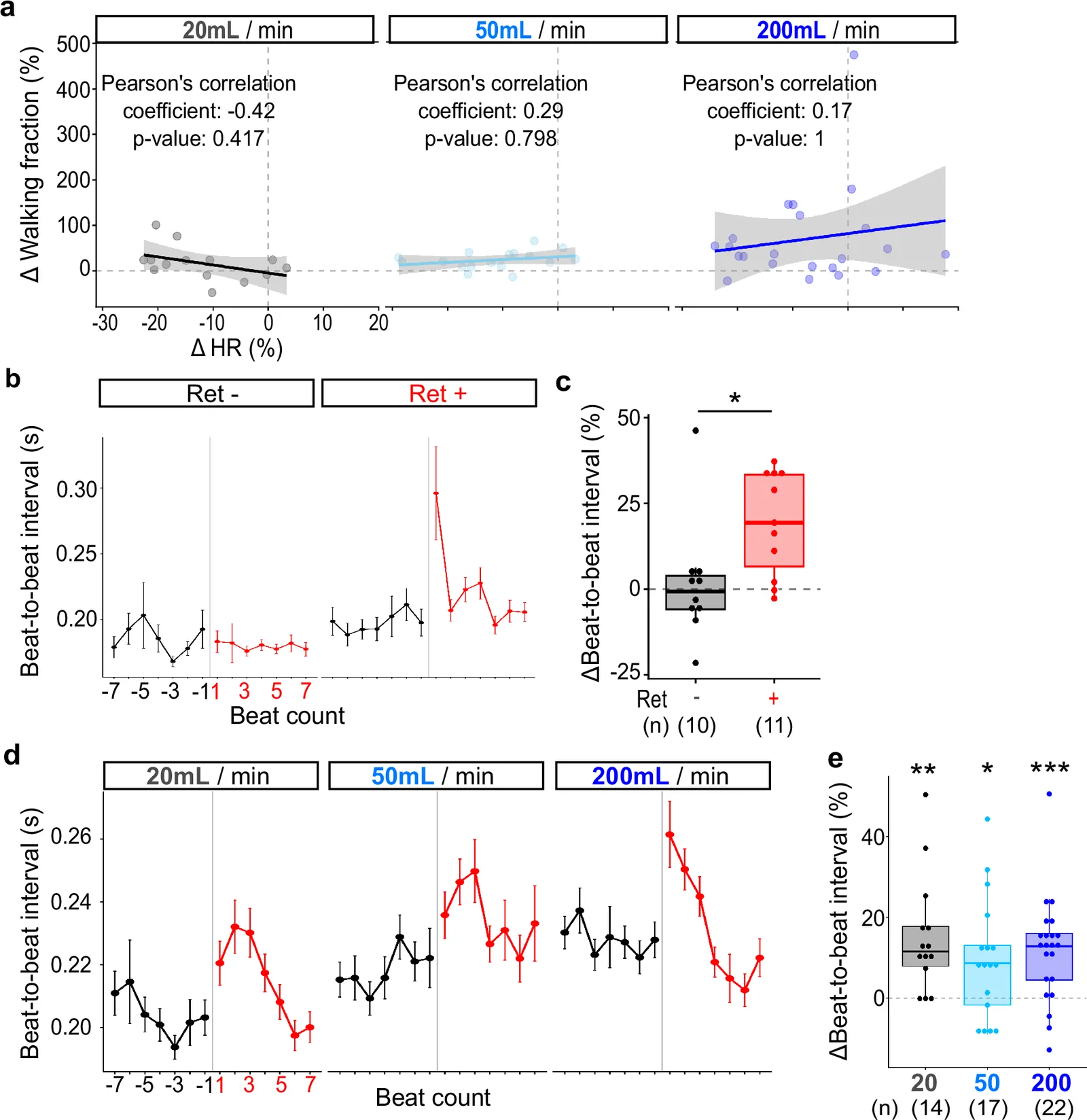
The effects of optogenetic cardiac deceleration on beat-to-beat interval. (**a**) Correlations between changes in walking fraction and HR. N=14, 17, 22 for 20, 50, and 200 mL/min, respectively. Pearson’s correlation coefficient, followed by Bonferroni correction. (**b**) Beat-to-beat interval (in seconds) plotted against the beat count around puff onset (gray vertical line). Dots and bars indicate mean and SEM, respectively. N=11, 10 for ret -, +, respectively. (**c**) Change in beat-to-beat interval, calculated for each fly as the % difference between the mean interval during three beats following stimulation onset and the mean interval during three beats preceding stimulation. N=11, 10 for ret -, +, respectively. *p<0.05, Wilcoxon’s signed rank test. (**d**) Beat-to-beat interval (in seconds) plotted against the beat count around puff onset (gray vertical line). Dots and bars indicate mean and SEM, respectively. N=14, 17, 22 for 20, 50, and 200 mL/min, respectively. Figure 1—figure supplement 2e is shown here again for comparison. (**e**) Change in beat-to-beat interval, calculated for each fly as the % difference between the mean interval during three beats following stimulation onset and the mean interval during three beats preceding stimulation. N=14, 17, 22 for 20, 50, and 200 mL/min, respectively. ***p<0.001, **p<0.01, *p<0.05, Wilcoxon’s signed rank test followed by Bonferroni correction. Figure 1—figure supplement 2f is shown here again for comparison.

**Video 4.** Optogenetic activation of DA-WED neurons induces cardiac deceleration and promotes locomotion. The playback speed is reduced to 0.25 of the original file.

An alternative interpretation is that DA-WED neurons function as mere puff detectors that broadly regulate puff-evoked behaviors. If this were the case, manipulating these neurons should affect all behavioral outputs induced by puff. In addition to promoting locomotion, air puff suppressed grooming predominantly within 3 s of puff onset, consistently across puff strengths; grooming during the later 10–20 s window was variable, either remaining suppressed, returning to baseline, or reversing to an increase depending on puff strength (Figure 5—figure supplement 1a–c). Silencing DA-WED neurons failed to disrupt puff-induced grooming suppression (Figure 5—figure supplement 1d–f), despite markedly reducing cardiac deceleration (Figure 4b and c) and locomotion (Figure 5a and b). Likewise, photoactivation of DA-WED neurons failed to suppress grooming (Figure 5—figure supplement 1g–i), while robustly inducing cardiac deceleration (Figure 4i and j) and locomotion (Figure 5c and d). Thus, manipulation of DA-WED neurons does not reproduce the full behavioral repertoire triggered by puff. These findings argue against the idea that DA-WED neurons function as mere puff detectors and instead support a more specific role in coordinating cardiac and locomotor responses.

To directly test whether cardiac deceleration can influence locomotion, we optogenetically induced cardiac deceleration of a magnitude comparable to the puff-induced response by expressing the light-gated anion channel GtACR1 in cardiomyocytes using Hand-GAL4 (Figure 5e). We selected an anion channel to prolong diastole, unlike a previous report using a cation channel (Men et al., 2020), as puff-induced cardiac deceleration is characterized by elongated diastolic intervals (Figure 1c). To approximate the 20–23 ms beat-to-beat prolongation evoked by mechanical threat (Figure 1; Figure 1—figure supplement 2e, f), we used 28 ms (2-frame) green light pulses. The light pulses transiently arrested the heart in diastole (Figure 5f and g; Video 5), reducing HR by ~10% (Figure 5h and i), comparable to puff- and DA-WED-induced deceleration (Figure 1d and e; Figure 4i and j). Notably, optogenetic induction of cardiac deceleration in cardiomyocytes—independent of central dopaminergic activation—was accompanied by increased locomotion (Figure 5j and k). In contrast, this manipulation failed to alter grooming behavior (Figure 5—figure supplement 1j–l), suggesting a selective cardiac influence on locomotion. Furthermore, optogenetically induced changes in HR and locomotion were strongly anti-correlated fly-wise in the retinal-fed test group (Pearson’s correlation coefficient: –0.81, p-value: 0.00538) but not in the control group without retinal (Pearson’s correlation coefficient: 0.43, p-value: 0.436; Figure 5l). This contrasts with puff-induced responses, in which changes in HR and locomotion were not significantly correlated (Figure 5—figure supplement 2a). This lack of correlation may reflect contributions from cardiac-state-independent locomotor pathways. These observations are consistent with the possibility that cardiac deceleration may influence locomotor responses upon mechanical threat (Figure 5m; Figure 5—figure supplement 1m). One limitation is that our optogenetically induced cardiac pattern (Figure 5g; Figure 5—figure supplement 2b, c) is not necessarily identical to its mechanically induced counterpart (Figure 1c; Figure 5—figure supplement 2d, e), which could have influenced locomotion. We also note that retinal-unfed controls showed a modest locomotor increase following green-light illumination (Figure 5j and k), leaving open whether cardiac deceleration directly promotes locomotion or potentiates a light-evoked locomotion. In either case, our data support a modulatory influence of cardiac state on locomotor output.

**Video 5.** Optogenetic cardiac deceleration promotes locomotion. The playback speed is reduced to 0.25 of the original file.

Altogether, these results identify a group of dopaminergic neurons that mediate threat-induced cardiac deceleration and point to a potential role of cardiac dynamics in shaping locomotor responses during threat.

## Discussion

Here, we demonstrate that mechanical threat engages dopaminergic neurons that induce rapid cardiac deceleration and is associated with increased locomotion. Air puff stimulation elicited both cardiac deceleration and locomotor activation (Figure 1), and we identified two pairs of dopaminergic neurons, termed DA-WED neurons, as key mediators of the cardiac response (Figure 4). Silencing these neurons attenuated threat-induced cardiac deceleration and locomotion, whereas optogenetic activation of DA-WED neurons elicited both responses in the absence of threat (Figures 4 and 5a–d). Moreover, direct optogenetic cardiac deceleration was accompanied by increased locomotion (Figure 5e–l), suggesting that cardiac deceleration may influence behavioral responses. Together, these findings support a model in which descending dopaminergic control of cardiac activity contributes to the coordination of physiological and behavioral responses to threat, potentially via interoceptive feedback from the heart.

### Cardiac deceleration is coordinated with defensive locomotion

Our data show that air puff stimulation induces cardiac deceleration together with increased locomotion (Figure 1). Across puff strengths, locomotion scaled with stimulus strength, whereas the cardiac deceleration did not: measured as time-binned HR, it was less pronounced at stronger puffs, while measured as the beat-to-beat interval it was comparable across strengths (Figure 1d and e; Figure 1—figure supplement 2e, f). This suggests that cardiac deceleration is likely not simply a byproduct of locomotion.

Although cardiac deceleration is often associated with freezing in mammals (Hagenaars et al., 2014), cardiac deceleration has also been observed during early threat detection and orienting responses (Skora et al., 2022), suggesting that it reflects engagement of defensive processing rather than motor inhibition per se. Consistent with this view, dissociation between threat-evoked cardiac modulation and freezing has been reported across taxa. In mammals, for example, disruption of autonomic output can abolish cardiac deceleration without eliminating freezing (Koba et al., 2016). Similarly, in decapod crustaceans and *Drosophila*, visual stimuli elicit reproducible and time-locked changes in HR that accompany both freezing and fleeing (Pérez-Schuster et al., 2024; Burnovicz et al., 2009; Barrios et al., 2021). While these studies establish that cardiac modulation can be dissociated from freezing, our findings additionally demonstrate that cardiac deceleration can co-occur with a predominantly locomotor response to mechanical threat, and can itself causally contribute to locomotion (Figure 5e–m). Cardiac deceleration is therefore not restricted to passive defensive states, but can act upstream of active locomotor responses.

### Dopaminergic neurons in the brain are responsible for puff-induced cardiac deceleration

The present study identifies two pairs of dopaminergic neurons in the brain, termed DA-WED neurons, as key mediators of puff-induced cardiac deceleration. Silencing/ photoactivating DA-WED neurons disrupts/ mimics puff-induced cardiac deceleration, respectively (Figure 4b, c and h–j). Furthermore, DA-WED neurons exhibit calcium elevation upon air puffs (Figure 4d–g). Moreover, knockdown of the dopamine synthetic enzyme *ple* in DA-WED neurons suppresses puff-induced cardiac deceleration (Figure 3—figure supplement 1g–i), suggesting that dopamine release from these neurons is required for cardiac deceleration.

Circulatory dopamine has long been associated with cardiac acceleration across species (Neumann et al., 2023). In *Drosophila*, pharmacological application of DA to the exposed heart accelerates the heartbeat (Zornik et al., 1999; Zhu et al., 2016; Johnson et al., 1997; Hillyer, 2018), seemingly at odds with our findings. However, the responsible source of circulatory DA or DA receptor, as well as whether DA directly acts on cardiomyocytes or instead through some yet unidentified cells, remain elusive. In mammals, cardiomyocytes per se synthesize DA and express DA receptors, hinting at an autocrine mechanism (Neumann et al., 2023). Circulatory DA also increases the levels of epinephrine and norepinephrine, complicating the interpretation of ‘DA-induced’ cardiac acceleration (Ziegler et al., 1985). From our ple RNAi experiment (Figure 3—figure supplement 1g–i), DA released from DA-WED neurons appears indispensable for puff-induced cardiac deceleration. Notably, neurites of DA-WED neurons remain confined to the brain, suggesting that DA-WED neurons engage circulation or yet unidentified neuronal circuitry to indirectly modulate the heartbeat. Circulation is unlikely to mediate DA-WED neurons’ function, however, given that the air puff and DA-WED activation both induce cardiac deceleration within a second (Figures 1 and 4). Interestingly, DA acting as a local neuromodulator to drive cardiac deceleration has been documented in mammals by a few literatures, either in the ventral tegmental area (VTA) or lateral habenula (LHb) (Kirouac and Pittman, 2000; Sato et al., 2024). Together, our findings and previous literature suggest that dopamine may act not only as a circulating hormone that accelerates HR, but as a centrally deployed neuromodulator capable of driving cardiac deceleration during defensive states.

### DA-WED neurons coordinate cardiac and behavioral threat responses

Across species, threatening stimulation elicits cardiac and behavioral responses in an orchestrated fashion. Cardiac interoception is postulated to serve as key feedback to link cardiac and behavioral responses (Lovelace et al., 2024). Cardiac interoception is mediated by sensory neurons innervating the heart and aortic arch (Lovelace et al., 2024), which convey information to ACC, insular, and prefrontal cortex (Shivkumar et al., 2016) through brain stem NTS and hypothalamic PVN (Shivkumar et al., 2016). These ascending pathways have been proposed to modulate learned fear (Klein et al., 2021), anxiety-like behavior (Hsueh et al., 2023), or sleep (Yao et al., 2022). Although models propose that descending control of the heart and ascending interoceptive feedback jointly shape defensive behavior, experimental validation of this bidirectional framework within a single system has been limited.

Our results provide partial support for such a model. We show that (1) mechanical threat induces cardiac deceleration and locomotion (Figure 1), (2) DA-WED neurons in the brain are responsible for threat-induced cardiac deceleration and locomotion (Figure 4; Figure 5a–d), and (3) optogenetic cardiac deceleration is accompanied by increased locomotion (Figure 5e–l). Our data points to a scenario in which DA-WED neurons induce cardiac deceleration upon mechanical threat, which may in turn influence threat-induced locomotion (Figure 5m). We note, however, that our data does not exclude the possibility that DA-WED neurons might influence locomotion at least partially independent of its role in cardiac deceleration.

Notably, the behavioral consequences of cardiac deceleration appear to be selective rather than global. Although air puff stimulation suppressed grooming, neither silencing nor activation of DA-WED neurons significantly affected grooming responses (Figure 5—figure supplement 1a–i), despite robust effects on cardiac deceleration and locomotion (Figures 4 and 5). Similarly, direct optogenetic induction of cardiac deceleration failed to influence grooming behavior (Figure 5—figure supplement 1j–l). These dissociations indicate that cardiac feedback does not uniformly modulate defensive behaviors but preferentially facilitates locomotor output. Such selectivity is consistent with the possibility that interoceptive signals from the heart may bias specific behavioral strategies rather than simply increasing overall arousal.

### Cardiac dynamics can modulate behavior

Our findings echo a growing body of work linking cardiac dynamics to behavior across distant taxa. In larval zebrafish, cardiac dynamics and behavior are coupled during threat (Herrera et al., 2026), and dedicated vagal sensory neurons encode HR, including its decreases, through a stimulus-invariant population code (Hernandez-Nunez et al., 2025). In mice, optogenetically induced tachycardia can drive anxiety-like behavior (Hsueh et al., 2023). And here we find that cardiac deceleration accompanies, and can promote, locomotion in *Drosophila*. Cardiac influence on behavior thus spans insects, fish, and mammals.

How cardiac state might be sensed in *Drosophila* is unknown, and several routes are conceivable. *Drosophila* heart receives rich neuronal innervation (Dulcis and Levine, 2005; Dulcis and Levine, 2003), although whether these neurons relay sensory information from the heart is unknown. If so, neurons could directly detect the heartbeat, analogous to the cardiac afferents that mediate cardiac interoception in vertebrates (Hernandez-Nunez et al., 2025). Alternatively, the heartbeat could be sensed mechanically or through gas-exchange-related cues. In mammals, the cardiac pressure pulse can be detected directly within the brain by mechanosensitive central neurons (Jammal Salameh et al., 2024). In zebrafish, cardiac activity can causally influence behavior through its oxygen supply (Herrera et al., 2026). In insects, although the circulatory architecture differs, the heartbeat contributes to pressure changes in the hemocoel and tracheal system and resultant gas exchange (Wasserthal, 2012; Wasserthal, 2014), which could in principle be detected. While the present study does not distinguish among these possibilities, establishing how cardiac state is sensed would provide a foundation for dissecting how central commands and peripheral physiological states interact to coordinate survival behaviors.

### Limitations of this study

While our data are consistent with a model in which DA-WED neurons couple cardiac deceleration to locomotor responses upon mechanical threat, several questions remain. Although cardiac deceleration can promote locomotion, we have not directly tested whether it is required for DA-WED–evoked locomotion, leaving open the possibility of parallel downstream pathways. In addition, the sensory mechanisms that relay cardiac state to locomotor circuits in *Drosophila* remain unidentified. Finally, despite the rapid timescale and behavioral specificity of our manipulations, we cannot fully exclude indirect physiological contributions secondary to altered cardiac dynamics. Future dissection of descending and ascending circuits will clarify these mechanisms.

Overall, we identify a discrete population of dopaminergic neurons that drive rapid cardiac deceleration in response to mechanical threat. We further show that these neurons coordinate cardiac responses and are associated with behavioral components of the defensive response, potentially by engaging interoceptive feedback from the heart. Our findings establish *Drosophila* as a tractable model for dissecting how exteroceptive and interoceptive signals are integrated to couple organ physiology with adaptive behavior, opening new avenues for understanding the neural logic by which internal and external states are coordinated to promote survival.

## Materials and methods

**Key resources table:** 

Reagent type (species) or resource	Designation	Source or reference	Identifiers	Additional information
Strain, strain background (*Drosophila melanogaster*, male)	Canton-S (CS)	Other		Gift from Dr. David Anderson (California Institute of Technology)
Genetic reagent (*D. melanogaster*)	Hand-GFP	Other		Gift from Dr. Zhe Han (University of Maryland)
Genetic reagent (*D. melanogaster*)	Hand-GAL4; R88D05-GAL4	Bloomington Drosophila Stock Center	BDSC:48396; RRID:BDSC_48396	
Genetic reagent (*D. melanogaster*)	TrH-GAL4	Bloomington Drosophila Stock Center	BDSC:38388; RRID:BDSC_38388	
Genetic reagent (*D. melanogaster*)	Tdc2-GAL4	Bloomington Drosophila Stock Center	BDSC:9313; RRID:BDSC_9313	
Genetic reagent (*D. melanogaster*)	TH-GAL4; ple-GAL4	Bloomington Drosophila Stock Center	BDSC:8848; RRID:BDSC_8848	
Genetic reagent (*D. melanogaster*)	TH-C-GAL4; TH-D-GAL4; TH-F1-GAL4	Other		Gift from Dr. Mark N. Wu (Johns Hopkins University)
Genetic reagent (*D. melanogaster*)	Vmat-R76F01-GAL4; Vmat-R76F05-GAL4; Ddc-R61H03-GAL4; Ddc-R60F07-GAL4; DAT-R55C10-GAL4	Other		Gift from Dr. Mark N. Wu (Johns Hopkins University)
Genetic reagent (*D. melanogaster*)	Vmat-R76F01-AD; Vmat-R76F05-AD; Ddc-R60F07-AD; Vmat-R76F01-DBD; Vmat-R76F05-DBD; Ddc-R60F07-DBD	Other		Gift from Dr. Mark N. Wu (Johns Hopkins University). Split-GAL4 hemidrivers.
Genetic reagent (*D. melanogaster*)	TH-FLP	Other		Gift from Dr. Mark N. Wu (Johns Hopkins University)
Genetic reagent (*D. melanogaster*)	UAS-FsF-mCD8GFP (UAS-FRT-stop-FRT-mCD8::GFP)	Other		Gift from Dr. Mark N. Wu (Johns Hopkins University)
Genetic reagent (*D. melanogaster*)	Vmat-R76F02-GAL4; R76F02-AD; R76F02-DBD; Dat-B	Other		Gift from Dr. Mark N. Wu (Johns Hopkins University). Used in Figure 3b and d.
Genetic reagent (*D. melanogaster*)	VT046828-AD	Bloomington Drosophila Stock Center	BDSC:73115; RRID:BDSC_73115	
Genetic reagent (*D. melanogaster*)	R12D12-DBD	Bloomington Drosophila Stock Center	BDSC:69213; RRID:BDSC_69213	
Genetic reagent (*D. melanogaster*)	R48A08-AD	Bloomington Drosophila Stock Center	BDSC:70707; RRID:BDSC_70707	
Genetic reagent (*D. melanogaster*)	VT008692-DBD	Bloomington Drosophila Stock Center	BDSC:86909; RRID:BDSC_86909	
Genetic reagent (*D. melanogaster*)	empty-AD; empty-DBD	Bloomington Drosophila Stock Center	BDSC:79603; RRID:BDSC_79603	
Genetic reagent (*D. melanogaster*)	R38B08-AD	Bloomington Drosophila Stock Center	BDSC:71032; RRID:BDSC_71032	
Genetic reagent (*D. melanogaster*)	R81E10-DBD	Bloomington Drosophila Stock Center	BDSC:68529; RRID:BDSC_68529	
Genetic reagent (*D. melanogaster*)	VT043775-DBD	Bloomington Drosophila Stock Center	BDSC:73728; RRID:BDSC_73728	
Genetic reagent (*D. melanogaster*)	R70G12-GAL4	Bloomington Drosophila Stock Center	BDSC:39552; RRID:BDSC_39552	
Genetic reagent (*D. melanogaster*)	R74C07-GAL4; Aes1N-GAL4	Bloomington Drosophila Stock Center	BDSC:39847; RRID:BDSC_39847	
Genetic reagent (*D. melanogaster*)	Tes1N-GAL4	Kyoto Drosophila Stock Center	DGGR:103660; RRID:DGGR_103660	
Genetic reagent (*D. melanogaster*)	iav-GAL4	Bloomington Drosophila Stock Center	BDSC:52273; RRID:BDSC_52273	
Genetic reagent (*D. melanogaster*)	R48A07-GAL4	Bloomington Drosophila Stock Center	BDSC:50340; RRID:BDSC_50340	
Genetic reagent (*D. melanogaster*)	UAS-TNT	Bloomington Drosophila Stock Center	BDSC:28838; RRID:BDSC_28838	
Genetic reagent (*D. melanogaster*)	UAS-Kir2.1::EGFP	Bloomington Drosophila Stock Center	BDSC:6596; RRID:BDSC_6596	
Genetic reagent (*D. melanogaster*)	UAS-FsF-Kir2.1 (UAS FRT-stop-FRT-Kir2.1)	Pther		Gift from Dr. Barry Dickson (The University of Queensland)
Genetic reagent (*D. melanogaster*)	20xUAS-CsChrimson::mVenus	Bloomington Drosophila Stock Center	BDSC:55135; RRID:BDSC_55135	
Genetic reagent (*D. melanogaster*)	UAS-FsF-CsChrimson::mVenus (UAS-FRT-stop-FRT-CsChrimson::mVenus)	Other		Gift from Dr. Barry Dickson (The University of Queensland)
Genetic reagent (*D. melanogaster*)	20xUAS-CsChrimson::mCherry	Bloomington Drosophila Stock Center	BDSC:82181; RRID:BDSC_82181	
Genetic reagent (*D. melanogaster*)	tub-FRT-GAL80-FRT (tub >GAL80>)	Bloomington Drosophila Stock Center	BDSC:38881; RRID:BDSC_38881	
Genetic reagent (*D. melanogaster*)	UAS-GtACR1::tdTomato (III)	Other		Gift from Dr. Tetsuya Tabata (The University of Tokyo)
Genetic reagent (*D. melanogaster*)	UAS-dTrpA1	Bloomington Drosophila Stock Center	BDSC:26263; RRID:BDSC_26263	
Genetic reagent (*D. melanogaster*)	UAS-mCD8::GFP	Bloomington Drosophila Stock Center	BDSC:32186; RRID:BDSC_32186	
Genetic reagent (*D. melanogaster*)	UAS-mCherry	Bloomington Drosophila Stock Center	BDSC:52267; RRID:BDSC_52267	
Genetic reagent (*D. melanogaster*)	UAS-RedStinger	Bloomington Drosophila Stock Center	BDSC:8547; RRID:BDSC_8547	
Genetic reagent (*D. melanogaster*)	UAS-IVS-jGCaMP7s	Bloomington Drosophila Stock Center	BDSC:79032; RRID:BDSC_79032	
Genetic reagent (*D. melanogaster*)	UAS-GFP RNAi (UAS-EGFP RNAi)	Bloomington Drosophila Stock Center	BDSC:41560; RRID:BDSC_41560	
Genetic reagent (*D. melanogaster*)	UAS-Ple RNAi	Bloomington Drosophila Stock Center	BDSC:65875; RRID:BDSC_65875	
Genetic reagent (*D. melanogaster*)	Dop1R1-attP; Dop1R2-attP; Dop2R-attP; DopEcR-attP	Other		Gift from Dr. Yi Rao (Peking University)
Genetic reagent (*D. melanogaster*)	TrH-attP	Other		Gift from Dr. Yi Rao (Peking University)
Antibody	Anti-Bruchpilot nc82 (mouse monoclonal)	Developmental Studies Hybridoma Bank	DSHB:nc82; RRID:AB_2314866	IHC (1:10)
Antibody	Goat anti-mouse IgG Alexa Fluor 633 (goat polyclonal)	Molecular Probes / Thermo Fisher Scientific	Cat#A-21050; RRID:AB_2535718	IHC (1:100)
Chemical compound, drug	All-trans retinal	Sigma-Aldrich	Cat#R2500-100MG	
chemical compound, drug	Brilliant blue FCF	Wako Pure Chemical Industries	CAS:3844-45-9	
Chemical compound, drug	Sulforhodamine B	Sigma-Aldrich	CAS:3520-42-1	
Other	Vectashield	Vector Laboratories	Cat#H-1000	
Other	Bondic UV-curable adhesive	Bondic	Cat#BD-SKCJ	
Software, algorithm	SLEAP	Pereira et al., 2022	RRID:SCR_021382	
Software, algorithm	ImageJ	NIH	RRID:SCR_003070	
Software, algorithm	Python	Python Software Foundation	RRID:SCR_008394	
Software, algorithm	R	R Core Team	RRID:SCR_001905	
Software, algorithm	Custom analysis code (heartbeat detection, behavior classification, statistics)	This paper; https://github.com/mtsuji172/heart_behavior (copy archived at Tsuji, 2026)		v1.0.0

### Fly stocks

Canton-S (CS) was used as the wild-type strain and was a gift from Dr. David Anderson (California Institute of Technology).

The following strains were obtained from Bloomington Stock Center (Indiana University): TrH-GAL4 (#38388), Tdc2-GAL4 (#9313), TH-GAL4 (#8848), UAS-TNT (#28838), UAS-Kir2.1::EGFP (#6596), UAS-CsChrimson::mVenus (#55135), UAS-dTrpA1 (#26263), UAS-mCD8::GFP (#32186), UAS-RedStinger (#8547), UAS-IVS-jGCaMP7s (#79032), UAS-GFP RNAi (#41560), UAS-Ple RNAi (#65875), VT046828-AD (#73115), R12D12-DBD (#69213), R48A08-AD (#70707), VT008692-DBD (#86909), empty-AD; empty-DBD (#79603), R70G12-GAL4 (#39552), R38B08-AD (#71032), R81E10-DBD (#68529), VT043775-DBD (#73728), R74C07-GAL4 (#39847), Aes1N-GAL4 (#39847), iav-GAL4 (#52273), R48A07-GAL4 (#50340), UAS-mCherry (#52267), Hand-GAL4 (R88D05-GAL4; #48396), 20xUAS-CsChrimson::mCherry (#82181), tub-FRT-GAL80-FRT (#38881).

The following strains were obtained from Kyoto *Drosophila* Stock Center (Kyoto Institute of Technology): Tes1N-GAL4 (#103660).

Hand-GFP was a gift from Dr. Zhe Han (The University of Maryland).

UAS-FRT-stop-FRT-Kir2.1, and UAS-FRT-stop-FRT-CsChrimson::mVenus were gifts from Dr. Barry Dickson (The University of Queensland).

TH-C-GAL4, TH-D-GAL4, TH-F1-GAL4, Vmat-R76F01-GAL4, Vmat-R76F02-GAL4, Vmat-R76F05-GAL4, Ddc-R61H03-GAL4, Ddc-R60F07-GAL4, DAT-R55C10-GAL4, DAT-B, Vmat-R76F01-AD, Vmat-R76F02-AD, Vmat-R76F05-AD, Ddc-R60F07-AD, Vmat-R76F01-DBD, Vmat-R76F02-DBD, Vmat-R76F05-DBD, Ddc-R60F07-DBD, TH-FLP, UAS-FsF-mCD8GFP were gifts from Dr. Mark N. Wu (Johns Hopkins University).

*Dop1R1-attP*, *Dop1R2-attP*, *Dop2R-attP*, *DopEcR-attP, TrH-attP* were gifts from Dr. Yi Rao (Peking University).

UAS-GtACR1::tdTomato (III) was a gift from Dr. Tetsuya Tabata (The University of Tokyo).

Genotypes and sample sizes used in this study are summarized in Supplementary file 1.

### Fly husbandry

Flies were maintained on conventional cornmeal-agar-molasses medium under a 9 AM:9 PM light/dark cycle at 25 °C and 60+/-5% humidity. Flies were collected 0–1 day post eclosion and housed in a group of 9–10 for 2–5 days before testing unless otherwise stated. Male flies were used throughout except for Figure 2—figure supplement 1h, i, in which both male and female were used. All experiments were carried out between 10 AM and 8 PM at 25±0.8 °C and 60±5% humidity.

### Heartbeat and behavior recording

Flies were tethered above an air-supported foam ball (8 mm diameter; Yuzawaya, Japan) mounted in a custom sphere holder. Airflow was continuously monitored using a digital flowmeter (MF-FP10N-H06-010, Horiba Estec Inc) and maintained at 500±10 mL/min. Behavioral imaging was performed from the right side under 850 nm infrared illumination using a CMOS camera (GE60, Himawari; 640×480 pixels). Simultaneously, cardiac activity was imaged from the dorsal side through the intact cuticle under 850 nm illumination using a second CMOS camera (CS135MU, Thorlabs; 800×500 pixels). Behavioral and cardiac imaging streams were externally triggered and synchronized at 70 Hz using an Arduino Uno board.

Immediately prior to the experiment, flies were briefly (<1 min) cold-anesthetized, and the dorsal thorax and abdomen were glued to a coverslip using UV-curable adhesive (Bondic BD-SKCJ) polymerized with 405 nm UV light (OSV5XME1C1E, Akizuki Electronics Inc). The coverslip was positioned above the air-supported ball, allowing the fly to freely walk on the ball. Each recording session, except for DA-WED optogenetic activation experiment, consisted of a 20 min acclimation period followed by puff-response testing (950 s). During puff-response testing, a 500 ms air puff was delivered once every 90 s for a total of 10 puffs. Recording continued for 30 s after the final puff. DA-WED optogenetic activation experiment consisted of a 20 min acclimation period followed by photoactivation-response testing (950 s), in which a 1 s 617 nm light was delivered once every 90 s for a total of two (see “Optogenetic activation of DA-WED neurons’ below), to avoid potential desensitization of CsChrimson.

### Heartbeat (without behavior) recording

Cardiomyocytes expressing GFP were imaged dorsally through the intact cuticle. Images (104×50 pixels) were acquired at 24 Hz using a Leica SP8 confocal microscope equipped with a 10×objective lens (NA 0.4).

Immediately before recording, flies were briefly (<1 min) cold-anesthetized, and the ventral thorax and abdomen were glued to a glass slide using UV-curable adhesive (Bondic BD-SKCJ) polymerized with 405 nm UV light (OSV5XME1C1E, Akizuki Electronics Inc). The slide was positioned beneath the objective lens for imaging. Each recording session consisted of a 10 min acclimation period followed by puff-response testing (350 s total). A single puff-response test consisted of 1 min baseline recording, followed by a train of 10 air puffs delivered at 1 Hz (10 s total), and a subsequent 1 min post-stimulation recording. For each fly, the puff-response test was repeated twice with a 90 s inter-trial interval.

### Automatic heartbeat detection

To automatically detect individual heartbeats, we developed custom software tools. For infrared (IR)–based heart imaging obtained during simultaneous recordings of cardiac activity and behavior, the detection pipeline (Figure 1—figure supplement 1a) computed optical flow for each pixel between consecutive video frames. These optical flow measurements were used to quantify global cardiac relaxation (diastole) and contraction (systole). Time points corresponding to maximal contraction were identified as systolic peaks, and peak counts were used to compute heart rate (HR). The precision of the automated detection was evaluated by comparing both absolute HR values and percent changes in HR during air-puff stimulation relative to baseline with manual annotations. These analyses demonstrated that the automated method detected both absolute HR and stimulus-evoked HR changes with accuracy indistinguishable from manual scoring (Figure 1—figure supplement 1b, c).

To detect heartbeats in confocal microscopy images of GFP-expressing cardiomyocytes (recorded in the absence of behavioral measurements; Figure 2—figure supplement 1a), each frame was subtracted from the subsequent frame to compute pixel-wise differences between consecutive image pairs. This procedure generated positive and negative pixel values in regions surrounding the cardiomyocytes during cardiac contraction (systole) and relaxation (diastole). During systole, pixels within the heart tube exhibited positive values corresponding to the current positions of cardiomyocytes, whereas pixels outside the heart tube exhibited negative values corresponding to their previous positions; the opposite pattern was observed during diastole. Systolic and diastolic phases were distinguished by: (i) calculating the SD of the positions of positive-value pixels along the axis perpendicular to the heart tube (the y-axis in Figure 2—figure supplement 1a), denoted SD(t); (ii) calculating the standard deviation of the positions of negative-value pixels along the same axis, denoted SD(t–1); and (iii) computing the difference SD(t) − SD(t–1). Negative values of SD(t) − SD(t–1) indicated systole, whereas positive values indicated diastole (Figure 2—figure supplement 1a). Time points corresponding to maximal contraction were identified as systolic peaks, and peak counts were used to compute HR. The precision of this algorithm was evaluated as described above. Although absolute HR values were slightly overestimated (Figure 2—figure supplement 1b), stimulus-evoked changes in HR were captured with accuracy indistinguishable from manual annotation (Figure 2—figure supplement 1c).

To obtain a continuous HR time course, peak counts were binned into 0.2 s intervals, and the number of beats per bin was calculated for each fly and trial. For visualization of HR dynamics relative to stimulus onset, HR traces were aligned to puff onset (time 0) and averaged across repeated trials within each fly. In experiments with a single air puff, puff-induced HR modulation was quantified by comparing the mean HR during the 1 s following puff onset with the mean HR during the 10 s baseline period preceding puff onset. Percent change was calculated as [(HR_post − HR_baseline) / HR_baseline]×100. In experiments involving 10 puffs delivered over a 10 s window, HR modulation was quantified as the mean HR during the entire 10 s stimulation period relative to the 10 s pre-stimulus baseline.

### Automatic behavioral identification

To automatically identify behavior on a frame-by-frame basis, videos were analyzed offline using a custom Python-based pipeline. Body-part coordinates—including the joints of each leg, the eye, neck, haltere, and the rostral tip of the abdomen on the right side of the fly—were tracked using SLEAP software (Pereira et al., 2022). The tracked coordinates were subsequently transformed to normalize body length and orientation across individuals. Using these standardized coordinates, the speed of each body part was calculated for each pair of consecutive frames. Both the standardized coordinates and corresponding speed measurements were then provided as inputs to a custom support vector machine (SVM)–based classifier, which labeled each frame as ‘groom’, ‘walk’, or ‘halt’. The SVM was trained using the standardized body-part coordinates and speed features as predictors, with manually annotated behavioral labels serving as the dependent variables. For each fly and each time point relative to puff onset, we calculated the fraction of frames classified as walking or grooming across 10 repeated trials. For visualization of behavioral dynamics, these fraction time courses were aligned to puff onset (time 0) and averaged across trials within each fly. Puff-induced changes in walking and grooming were quantified by comparing the mean fraction during the 10 s and 3 s, respectively, following puff onset with the mean fraction during the 10 s baseline period preceding puff onset. Percent change was calculated as [(fraction_post − fraction_baseline) / fraction_baseline]×100.

### Mechanical stimulation

Mechanical stimulation was delivered through a glass pipette (tip diameter of ~2 mm), placed ~2 mm in front of the fly, connected with air hose to a compressor (EARTH MAN, Takagi, Japan) via a solenoid valve (EXA-C6-02C-4, CKD). The solenoid valve was controlled by an Arduino Uno board, which in turn received connection from a desktop computer running the Python program which orchestrated the video recordings and puff application. The mechanical stimulation was generated by alternating the solenoid valve between 500 ms-open and 500 ms-closed states (1 Hz). The mechanical stimulation lasted for either 1, 10, or 20 s, as specified in each figure. The air flow was monitored with a digital flowmeter (MF-FP10N-H06-010, Horiba estec. Inc) and was adjusted to either 20, 50, or 200 mL/min, as specified in each figure.

### Optogenetic activation of DA-WED neurons

Flies expressing CsChrimson, a red-shifted channelrhodopsin variant, in DA-WED neurons were raised on food containing 500 µM all-trans retinal for 2–3 days, in the dark, prior to testing. Photoactivation was performed by delivering a 1 s LED light pulse (617 nm, Thorlabs) to the fly’s head. The output for LED was measured with a power meter (S120C and PM120D, Thorlabs) at a position corresponding to the fly’s head, and was adjusted to 1 μW/mm^2^. Light stimuli were triggered by Python through an Arduino board connected to the LED driver.

### Optogenetic cardiac deceleration

Flies expressing GtACR1, a light-gated anion channel, in cardiomyocytes were raised on food containing 500 µM all-trans retinal for 2–3 days, in the dark, prior to testing. Photoactivation was performed by delivering a 28 ms-long LED light pulse (530 nm, Thorlabs) to the fly’s heart through the objective lens. Because mechanical threat transiently elongated beat-to-beat intervals typically by 20–23 ms, we chose 28 ms (2-frame, at our 70 Hz acquisition rate) stimulation to best mimic this effect, over 14 (1-frame) or 42 (3-frame) ms (Figure 1; Figure 1—figure supplement 2b). The light intensity was adjusted, for each fly prior to the recording, to the minimum intensity required for a light pulse of roughly 2 s to briefly stop the heartbeat. After the recording, the output for LED was measured with a power meter (S120C and PM120D, Thorlabs) at a position corresponding to the fly’s heart, and ranged from 9.88 to 21.8 μW/mm^2^. Light stimuli were triggered by Python through an Arduino board connected to the LED driver.

### Immunohistochemistry

The following antibodies were used: mouse nc82 (1:10 Developmental Studies Hybridoma Bank) and goat anti-mouse Alexa 633 (1:100, Molecular Probe #A21050). Whole brain immunohistochemistry was performed as described previously (Van Vactor et al., 1991; Hummel and Zipursky, 2004). Briefly, brains were dissected out in 0.3% PBST, and were fixed in 2% paraformaldehyde/PBS for 90 min at room temperature. Brains were then washed in 0.3% PBST for >3 hr, and were blocked in 10% normal goat serum/0.3% PBST for 30 min. Brains were incubated in the primary antibodies in 10% normal goat serum/0.3% PBST at 4 °C for 2 days. Brains were then washed in 0.3% PBST for >5 hr, and were blocked in 10% normal goat serum/0.3% PBST for 30 min. Brains were then incubated in the secondary antibodies in 10% normal goat serum/0.3% PBST at 4 °C for 2 days. Afterward, brains were washed in 0.3% PBST for >5 hr, and incubated in 50% glycerol/0.3% PBST for 2 hr at 4 °C. Lastly, brains were mounted on slide glass in Vectashield (Vector Laboratories). Images were taken with a Leica TCS SP8 confocal microscope, and processed in ImageJ (NIH) software.

### Calcium imaging

Two-photon calcium imaging was conducted as described previously (Tsuji et al., 2023) with a few modifications. Two-photon microscopy (Bergamo II, Thorlabs) with a 16× objective lens (16 x CFI LWP Plan Flour Objective, Nikon) and with near-infrared excitation (930 nm, Mai Tai 2, SpectraPhysics, Inc, Mountain View, CA) was used. Cuticle of the top of the flies’ head and the fat and trachea beneath it were surgically removed. The exposed brain was submerged in saline solution. The extracellular saline had the following composition (in mM): 103 NaCl, 3 KCl, 5 HEPES, 10 trehalose, 10 glucose, 7 sucrose, 26 NaHCO_3_, 1 NaH_2_PO_4_, 1.5 CaCl_2_, 4 MgCl_2_. We identified DA-WED neurons using the baseline fluorescence of GCaMP. Images were acquired from 1 plane at 152 frames per s (160x96 pixels, pixel size = 0.388 µm, dwell time = 0.246 µs). During calcium imaging, a large fraction of the fly’s body was glued to the holding plate, and part of the fly’s eye compounds was covered with the holder, and the fly was hung in the air. Each fly underwent the sequence of stimuli per fly once or twice, to scan DA-WED neurons on both hemispheres whenever possible. The time courses of ΔF/F_0_ were calculated by, for each bin, taking the percent difference from F 10 s prior to the onset of the initial puff application (t = –10 through 0 s in Figure 4f).

### Surgery

In search of sensory organs indispensable for air puffs to induce cardiac deceleration, halteres, wings, legs, and a3 segments of the antennae were surgically removed using a pair of forceps on ice just prior to testing. Flies that underwent cold anesthesia just prior to testing served as controls.

### Feeding preference test

Two days post eclosion, male flies were protein deprived by maintaining on 200 mM sucrose (vials with a kimwipe soaked with 2 mL of sucrose solution) for 8 days (kimwipe is exchanged every other day). We prepared 7.5% yeast and 200 mM sucrose in 0.9% of agar, color-labeled with either blue (brilliant blue FCF) or red (sulforhodamine B) tasteless food dyes. The two types of food mix, 1 mL each, were then aliquoted into 35 mm petri dish separated by a 5 × 5 × 35 mm acrylic bar into two chambers. A group of 70 flies starved for 3 hr in an empty vial with a kimwipe soaked with 2 mL of water were cold-anesthetized and transferred into the petri dish at ZT2 and allowed to feed for 2 hr in the dark. The flies were then killed by transferring the dish to –30 C, upside-down, and scored by examining the color of their abdomen. Preference Index (PI) for yeast was calculated as (# flies consuming yeast - # flies consuming sucrose)/(total # flies that fed). Flies with insufficiently colored abdomen were discarded as poorly fed flies.

### Data analyses

Data analyses were performed using our custom-written R and Python programs.

### Statistics

We did not predetermine the sample sizes. The distribution of normality of each group was assessed using the Shapiro-Wilk normality test. Parametric tests (unequal variances *t*-test known as Welch’s *t*-test or Dunnett’s test) were used only when distributions were deemed normal; otherwise, non-parametric tests were used (Wilcoxon signed rank test, Wilcoxon rank sum test, or Dunn’s test). All statistical tests were two-sided. We employed Bonferroni correction to correct for two tests, and correction implemented in Dunnett’s or Dunn’s test for more than two tests. p<0.05 after corrections for multiple comparisons was deemed statistically significant. Neither randomization nor blinding was performed for the group allocation during experiments or data analysis. All box plots are generated so that the center line indicates the median, box limits indicate upper and lower quartiles, and whiskers indicate 1.5 x interquartile range. All the statistical results are summarized in Supplementary file 2.

### Code availability

Source codes used in the current study are available at https://github.com/mtsuji172/heart_behavior, copy archived at Tsuji, 2026.

## Data Availability

All data generated or analysedanalyzed during this study are included in the manuscript and supporting files. Numerical source data for every figure and figure-supplement panel are provided in Source data 1; genotypes and sample sizes are listed in Supplementary file 1 and full statistical results in Supplementary file 2. Custom analysis code is available at https://github.com/mtsuji172/heart_behavior (copy archived at Tsuji, 2026).

## Additional files

MDAR checklist

Supplementary file 1. List of genotypes and sample sizes used in this study.

Supplementary file 2. Statistics of data presented in this study.

Source data 1. Numerical source data for all figure and figure-supplement panels (one worksheet per panel).

## References

[bib1] Alexander L, Wood CM, Gaskin PLR, Sawiak SJ, Fryer TD, Hong YT, McIver L, Clarke HF, Roberts AC (2020). Over-activation of primate subgenual cingulate cortex enhances the cardiovascular, behavioral and neural responses to threat. Nature Communications.

[bib2] Azzalini D, Buot A, Palminteri S, Tallon-Baudry C (2021). Responses to heartbeats in ventromedial prefrontal cortex contribute to subjective preference-based decisions. The Journal of Neuroscience.

[bib3] Babo-Rebelo M, Richter CG, Tallon-Baudry C (2016). Neural responses to heartbeats in the default network encode the self in spontaneous thoughts. The Journal of Neuroscience.

[bib4] Barrios N, Farias M, Moita MA (2021). Threat induces cardiac and metabolic changes that negatively impact survival in flies. Current Biology.

[bib5] Battaglia S, Thayer JF (2022). Functional interplay between central and autonomic nervous systems in human fear conditioning. Trends in Neurosciences.

[bib6] Burnovicz A, Oliva D, Hermitte G (2009). The cardiac response of the crab Chasmagnathus granulatus as an index of sensory perception. The Journal of Experimental Biology.

[bib7] Casto R, Nguyen T, Printz MP (1989). Characterization of cardiovascular and behavioral responses to alerting stimuli in rats. American Journal of Physiology-Regulatory, Integrative and Comparative Physiology.

[bib8] Clements J, Goina C, Hubbard PM, Kawase T, Olbris DJ, Otsuna H, Svirskas R, Rokicki K (2024). NeuronBridge: an intuitive web application for neuronal morphology search across large data sets. BMC Bioinformatics.

[bib9] Dana H, Sun Y, Mohar B, Hulse BK, Kerlin AM, Hasseman JP, Tsegaye G, Tsang A, Wong A, Patel R, Macklin JJ, Chen Y, Konnerth A, Jayaraman V, Looger LL, Schreiter ER, Svoboda K, Kim DS (2019). High-performance calcium sensors for imaging activity in neuronal populations and microcompartments. Nature Methods.

[bib10] Dielenberg RA, Carrive P, McGregor IS (2001). The cardiovascular and behavioral response to cat odor in rats: unconditioned and conditioned effects. Brain Research.

[bib11] Dulcis D, Levine RB (2003). Innervation of the heart of the adult fruit fly, *Drosophila melanogaster*. The Journal of Comparative Neurology.

[bib12] Dulcis D, Levine RB (2005). Glutamatergic innervation of the heart initiates retrograde contractions in adult *Drosophila melanogaster*. The Journal of Neuroscience.

[bib13] Farkas E, Jansen ASP, Loewy AD (1998). Periaqueductal gray matter input to cardiac-related sympathetic premotor neurons. Brain Research.

[bib14] Gordan R, Gwathmey JK, Xie LH (2015). Autonomic and endocrine control of cardiovascular function. World Journal of Cardiology.

[bib15] Green AL, Wang S, Purvis S, Owen SLF, Bain PG, Stein JF, Guz A, Aziz TZ, Paterson DJ (2007). Identifying cardiorespiratory neurocircuitry involved in central command during exercise in humans. The Journal of Physiology.

[bib16] Gross CT, Canteras NS (2012). The many paths to fear. Nature Reviews. Neuroscience.

[bib17] Hagenaars MA, Oitzl M, Roelofs K (2014). Updating freeze: aligning animal and human research. Neuroscience and Biobehavioral Reviews.

[bib18] Han Z, Olson EN (2005). Hand is a direct target of Tinman and GATA factors during Drosophila cardiogenesis and hematopoiesis. Development.

[bib19] Han Z, Yi P, Li X, Olson EN (2006). Hand, an evolutionarily conserved bHLH transcription factor required for Drosophila cardiogenesis and hematopoiesis. Development.

[bib20] Hashemi MM, Gladwin TE, de Valk NM, Zhang W, Kaldewaij R, van Ast V, Koch SBJ, Klumpers F, Roelofs K (2019). Neural dynamics of shooting decisions and the switch from freeze to fight. Scientific Reports.

[bib21] Headley DB, Kanta V, Kyriazi P, Paré D (2019). Embracing complexity in defensive networks. Neuron.

[bib22] Hermans EJ, Henckens M, Roelofs K, Fernández G (2013). Fear bradycardia and activation of the human periaqueductal grey. NeuroImage.

[bib23] Hernandez-Nunez L, Avrami J, Shi S, Markarian A, Boulanger-Weill J, Zarghani-Shiraz A, Ahrens M, Engert F, Fishman MC (2025). Emergence of functional heart-brain circuits in a vertebrate. bioRxiv.

[bib24] Herrera KJ, Zarghani-Shiraz A, Ahrens MB, Engert F, Fishman MC (2026). Synchronization of behavioral and cardiac dynamics in larval zebrafish. Cell Reports.

[bib25] Hillyer JF (2018). Insect heart rhythmicity is modulated by evolutionarily conserved neuropeptides and neurotransmitters. Current Opinion in Insect Science.

[bib26] Hillyer JF, Pass G (2020). The insect circulatory system: Structure, function, and evolution. Annual Review of Entomology.

[bib27] Horiuchi J, McDowall LM, Dampney RAL (2006). Differential control of cardiac and sympathetic vasomotor activity from the dorsomedial hypothalamus. Clinical and Experimental Pharmacology & Physiology.

[bib28] Hsueh B, Chen R, Jo Y, Tang D, Raffiee M, Kim YS, Inoue M, Randles S, Ramakrishnan C, Patel S, Kim DK, Liu TX, Kim SH, Tan L, Mortazavi L, Cordero A, Shi J, Zhao M, Ho TT, Crow A, Yoo ACW, Raja C, Evans K, Bernstein D, Zeineh M, Goubran M, Deisseroth K (2023). Cardiogenic control of affective behavioural state. Nature.

[bib29] Hummel T, Zipursky SL (2004). Afferent induction of olfactory glomeruli requires N-cadherin. Neuron.

[bib30] Jammal Salameh L, Bitzenhofer SH, Hanganu-Opatz IL, Dutschmann M, Egger V (2024). Blood pressure pulsations modulate central neuronal activity via mechanosensitive ion channels. Science.

[bib31] Johnson E, Ringo J, Dowse H (1997). Modulation of *Drosophila* heartbeat by neurotransmitters. Journal of Comparative Physiology: B, Biochemical, Systemic, and Environmental Physiology.

[bib32] Johnson E, Sherry T, Ringo J, Dowse H (2002). Modulation of the cardiac pacemaker of *Drosophila*: cellular mechanisms. Journal of Comparative Physiology: B, Biochemical, Systemic, and Environmental Physiology.

[bib33] Keller NR, Diedrich A, Appalsamy M, Tuntrakool S, Lonce S, Finney C, Caron MG, Robertson D (2004). Norepinephrine transporter-deficient mice exhibit excessive tachycardia and elevated blood pressure with wakefulness and activity. Circulation.

[bib34] Kirouac GJ, Pittman QJ (2000). A projection from the ventral tegmental area to the periaqueductal gray involved in cardiovascular regulation. American Journal of Physiology: Regulatory, Integrative and Comparative Physiology.

[bib35] Klein AS, Dolensek N, Weiand C, Gogolla N (2021). Fear balance is maintained by bodily feedback to the insular cortex in mice. Science.

[bib36] Koba S, Inoue R, Watanabe T (2016). Role played by periaqueductal gray neurons in parasympathetically mediated fear bradycardia in conscious rats. Physiological Reports.

[bib37] Koba S, Kumada N, Narai E, Kataoka N, Nakamura K, Watanabe T (2022). A brainstem monosynaptic excitatory pathway that drives locomotor activities and sympathetic cardiovascular responses. Nature Communications.

[bib38] Kreibig SD (2010). Autonomic nervous system activity in emotion: a review. Biological Psychology.

[bib39] Lang PJ, Davis M, Ohman A (2000). Fear and anxiety: animal models and human cognitive psychophysiology. Journal of Affective Disorders.

[bib40] Lebestky T, Chang JSC, Dankert H, Zelnik L, Kim YC, Han KA, Wolf FW, Perona P, Anderson DJ (2009). Two different forms of arousal in Drosophila are oppositely regulated by the dopamine D1 receptor ortholog DopR via distinct neural circuits. Neuron.

[bib41] Li SJ, Varga K, Archer P, Hruby VJ, Sharma SD, Kesterson RA, Cone RD, Kunos G (1996). Melanocortin antagonists define two distinct pathways of cardiovascular control by alpha- and gamma-melanocyte-stimulating hormones. The Journal of Neuroscience.

[bib42] Liu Q, Tabuchi M, Liu S, Kodama L, Horiuchi W, Daniels J, Chiu L, Baldoni D, Wu MN (2017). Branch-specific plasticity of a bifunctional dopamine circuit encodes protein hunger. Science.

[bib43] Lovelace JW, Ma J, Augustine V (2024). Defining cardioception: Heart-brain crosstalk. Neuron.

[bib44] Luan H, Peabody NC, Vinson CRR, White BH (2006). Refined spatial manipulation of neuronal function by combinatorial restriction of transgene expression. Neuron.

[bib45] Mais L, Hirsch P, Managan C, Wang K, Rokicki K, Svirskas RR, Dickson BJ, Korff W, Rubin GM, Ihrke G, Meissner GW, Kainmueller D (2021). PatchPerPixMatch for Automated 3d search of neuronal morphologies in light microscopy. bioRxiv.

[bib46] Majeed ZR, Stacy A, Cooper RL (2014). Pharmacological and genetic identification of serotonin receptor subtypes on *Drosophila* larval heart and aorta. Journal of Comparative Physiology: B, Biochemical, Systemic, and Environmental Physiology.

[bib47] Mao Z, Davis RL (2009). Eight different types of dopaminergic neurons innervate the *Drosophila* mushroom body neuropil: anatomical and physiological heterogeneity. Frontiers in Neural Circuits.

[bib48] Mastrianni JA, Palkovits M, Kunos G (1989). Activation of brainstem endorphinergic neurons causes cardiovascular depression and facilitates baroreflex bradycardia. Neuroscience.

[bib49] Matsukawa K (2012). Central command: control of cardiac sympathetic and vagal efferent nerve activity and the arterial baroreflex during spontaneous motor behaviour in animals. Experimental Physiology.

[bib50] Men J, Li A, Jerwick J, Li Z, Tanzi RE, Zhou C (2020). Non-invasive red-light optogenetic control of *Drosophila* cardiac function. Communications Biology.

[bib51] Nakamura K, Nakamura Y, Kataoka N (2022). A hypothalamomedullary network for physiological responses to environmental stresses. Nature Reviews. Neuroscience.

[bib52] Neumann J, Hofmann B, Dhein S, Gergs U (2023). Role of *Dopamine* in the Heart in Health and Disease. International Journal of Molecular Sciences.

[bib53] Pereira TD, Tabris N, Matsliah A, Turner DM, Li J, Ravindranath S, Papadoyannis ES, Normand E, Deutsch DS, Wang ZY, McKenzie-Smith GC, Mitelut CC, Castro MD, D’Uva J, Kislin M, Sanes DH, Kocher SD, Wang SS-H, Falkner AL, Shaevitz JW, Murthy M (2022). SLEAP: A deep learning system for multi-animal pose tracking. Nature Methods.

[bib54] Pérez-Schuster V, Salomón L, Bengochea M, Basnak MA, Velázquez Duarte F, Hermitte G, Berón de Astrada M (2024). Threatening stimuli elicit a sequential cardiac pattern in arthropods. iScience.

[bib55] Prescott SL, Liberles SD (2022). Internal senses of the vagus nerve. Neuron.

[bib56] Rahmouni K (2016). Cardiovascular regulation by the arcuate nucleus of the hypothalamus. Hypertension.

[bib57] Rajendran PS, Challis RC, Fowlkes CC, Hanna P, Tompkins JD, Jordan MC, Hiyari S, Gabris-Weber BA, Greenbaum A, Chan KY, Deverman BE, Münzberg H, Ardell JL, Salama G, Gradinaru V, Shivkumar K (2019). Identification of peripheral neural circuits that regulate heart rate using optogenetic and viral vector strategies. Nature Communications.

[bib58] Ran C, Boettcher JC, Kaye JA, Gallori CE, Liberles SD (2022). A brainstem map for visceral sensations. Nature.

[bib59] Rotstein B, Paululat A (2016). On the morphology of the *Drosophila* Heart. Journal of Cardiovascular Development and Disease.

[bib60] Samuels BC, Zaretsky DV, DiMicco JA (2002). Tachycardia evoked by disinhibition of the dorsomedial hypothalamus in rats is mediated through medullary raphe. The Journal of Physiology.

[bib61] Sato Y, Matsumoto M, Koganezawa T (2024). The dopaminergic system mediates the lateral habenula-induced autonomic cardiovascular responses. Frontiers in Physiology.

[bib62] Schiemann R, Lammers K, Janz M, Lohmann J, Paululat A, Meyer H (2018). Identification and in vivo characterisation of cardioactive peptides in *Drosophila melanogaster*. International Journal of Molecular Sciences.

[bib63] Schroeder C, Jordan J (2012). Norepinephrine transporter function and human cardiovascular disease. American Journal of Physiology. Heart and Circulatory Physiology.

[bib64] Sellin J, Albrecht S, Kölsch V, Paululat A (2006). Dynamics of heart differentiation, visualized utilizing heart enhancer elements of the *Drosophila melanogaster* bHLH transcription factor Hand. Gene Expression Patterns.

[bib65] Shivkumar K, Ajijola OA, Anand I, Armour JA, Chen P, Esler M, De Ferrari GM, Fishbein MC, Goldberger JJ, Harper RM, Joyner MJ, Khalsa SS, Kumar R, Lane R, Mahajan A, Po S, Schwartz PJ, Somers VK, Valderrabano M, Vaseghi M, Zipes DP (2016). Clinical neurocardiology defining the value of neuroscience‐based cardiovascular therapeutics. The Journal of Physiology.

[bib66] Signoret-Genest J, Schukraft N, L Reis S, Segebarth D, Deisseroth K, Tovote P (2023). Integrated cardio-behavioral responses to threat define defensive states. Nature Neuroscience.

[bib67] Silva BA, Gross CT, Gräff J, Gra J (2016). The neural circuits of innate fear: detection, integration, action, and memorization. Learning & Memory.

[bib68] Silvani A, Calandra-Buonaura G, Dampney RAL, Cortelli P (2016). Brain–heart interactions: physiology and clinical implications. Philosophical Transactions of the Royal Society A.

[bib69] Skora LI, Livermore JJA, Roelofs K (2022). The functional role of cardiac activity in perception and action. Neuroscience and Biobehavioral Reviews.

[bib70] Sujkowski A, Gretzinger A, Soave N, Todi SV, Wessells R (2020). Alpha- and beta-adrenergic octopamine receptors in muscle and heart are required for *Drosophila* exercise adaptations. PLOS Genetics.

[bib71] Tsubouchi A, Yano T, Yokoyama TK, Murtin C, Otsuna H, Ito K (2017). Topological and modality-specific representation of somatosensory information in the fly brain. Science.

[bib72] Tsuji M, Nishizuka Y, Emoto K (2023). Threat gates visual aversion via theta activity in Tachykinergic neurons. Nature Communications.

[bib73] Tsuji M (2026). Software Heritage.

[bib74] Van Vactor DL, Cagan RL, Krämer H, Zipursky SL (1991). Induction in the developing compound eye of *Drosophila*: multiple mechanisms restrict R7 induction to a single retinal precursor cell. Cell.

[bib75] Veerakumar A, Yung AR, Liu Y, Krasnow MA (2022). Molecularly defined circuits for cardiovascular and cardiopulmonary control. Nature.

[bib76] Wasserthal LT (2012). Influence of periodic heartbeat reversal and abdominal movements on hemocoelic and tracheal pressure in resting blowflies Calliphora vicina. The Journal of Experimental Biology.

[bib77] Wasserthal LT (2014). Periodic heartbeat reversals cause cardiogenic inspiration and expiration with coupled spiracle leakage in resting blowflies, Calliphora vicina. The Journal of Experimental Biology.

[bib78] Wessells RJ, Bodmer R (2004). Screening assays for heart function mutants in *Drosophila*. BioTechniques.

[bib79] White DW, Raven PB (2014). Autonomic neural control of heart rate during dynamic exercise: revisited. The Journal of Physiology.

[bib80] Wink J, van Delft R, Notenboom RGE, Wouters PF, DeRuiter MC, Plevier JWM, Jongbloed MRM (2020). Human adult cardiac autonomic innervation: Controversies in anatomical knowledge and relevance for cardiac neuromodulation. Autonomic Neuroscience.

[bib81] Wu G, Ma T, Hancock CE, Gonzalez S, Aryal B, Vaz S, Chan G, Palarca-Wong M, Allen N, Chung CI, Shu X, Liu Q (2024). Opposing GPCR signaling programs protein intake setpoint in *Drosophila*. Cell.

[bib82] Xie T, Ho MCW, Liu Q, Horiuchi W, Lin CC, Task D, Luan H, White BH, Potter CJ, Wu MN (2018). A genetic toolkit for dissecting dopamine circuit function in *Drosophila*. Cell Reports.

[bib83] Yao Y, Barger Z, Saffari Doost M, Tso CF, Darmohray D, Silverman D, Liu D, Ma C, Cetin A, Yao S, Zeng H, Dan Y (2022). Cardiovascular baroreflex circuit moonlights in sleep control. Neuron.

[bib84] Yoshimoto A, Morikawa S, Kato E, Takeuchi H, Ikegaya Y (2024). Top-down brain circuits for operant bradycardia. Science.

[bib85] Zhang F, Zhao Y, Han Z (2013). An in vivo functional analysis system for renal gene discovery in *Drosophila* pericardial nephrocytes. Journal of the American Society of Nephrology.

[bib86] Zhang N, Guo L, Simpson JH (2020). Spatial comparisons of mechanosensory information govern the grooming sequence in *Drosophila*. Current Biology.

[bib87] Zhu YC, Yocom E, Sifers J, Uradu H, Cooper RL (2016). Modulatory effects on *Drosophila* larva hearts: room temperature, acute and chronic cold stress. Journal of Comparative Physiology. B, Biochemical, Systemic, and Environmental Physiology.

[bib88] Ziegler MG, Kennedy B, Holland OB, Murphy D, Lake CR (1985). The effects of dopamine agonists on human cardiovascular and sympathetic nervous systems. International Journal of Clinical Pharmacology, Therapy and Toxicology.

[bib89] Zornik E, Paisley K, Nichols R (1999). Neural transmitters and a peptide modulate *Drosophila* heart rate. Peptides.

