# Supplementary material for: Dopaminergic neurons linking threat processing to cardiac modulation and locomotor responses
Source: eLife. 2026 Nov 5;15:RP111256. doi: 10.7554/eLife.111256 (PMC13739268; doi:10.7554/eLife.111256)
Supplement: Supplementary file 1 [file elife-111256-mdarchecklist1.docx]

**Materials Design Analysis Reporting (MDAR)**

**Checklist for Authors**

The [MDAR framework](https://osf.io/xfpn4/) establishes a minimum set of requirements in transparent reporting mainly applicable to studies in the life sciences.

*eLife* asks authors to **provide detailed information within their article** to facilitate the interpretation and replication of their work. Authors can also upload supporting materials to comply with relevant reporting guidelines for health-related research (see [EQUATOR Network](http://www.equator-network.org/%20)), life science research (see the BioSharing Information Resource), or animal research (see the [ARRIVE Guidelines](http://www.plosbiology.org/article/info:doi/10.1371/journal.pbio.1000412) and the [STRANGE Framework](https://doi.org/10.1038/d41586-020-01751-5); for details, see *eLife*’s [Journal Policies](https://reviewer.elifesciences.org/author-guide/journal-policies)). Where applicable, authors should refer to any relevant reporting standards materials in this form.

For all that apply, please note **where in the article** the information is provided. Please note that we also collect information about data availability and ethics in the submission form.

**Materials:**

| **Newly created materials** | **Indicate where provided: section/figure legend** | **N/A** |
| --- | --- | --- |
| The manuscript includes a dedicated "materials availability statement" providing transparent disclosure about availability of newly created materials including details on how materials can be accessed and describing any restrictions on access. | No newly created materials: all fly strains were obtained from public stock centres or were gifts from the cited laboratories (Methods, "Fly stocks"; Key Resources Table). | N/A |
|  |  |  |
| **Antibodies** | **Indicate where provided: section/figure legend** | **N/A** |
| For commercial reagents, provide supplier name, catalogue number and [RRID](https://scicrunch.org/resources), if available. | Methods, "Immunohistochemistry": mouse nc82 (DSHB, 1:10); goat anti-mouse Alexa Fluor 633 (Molecular Probes #A21050, 1:100). Also listed in the Key Resources Table. |  |
|  |  |  |
| **DNA and RNA sequences** | **Indicate where provided: section/figure legend** | **N/A** |
| Short novel DNA or RNA including primers, probes: Sequences should be included or deposited in a public repository. |  | N/A |
|  |  |  |
| **Cell materials** | **Indicate where provided: section/figure legend** | **N/A** |
| Cell lines: Provide species information, strain. Provide accession number in repository OR supplier name, catalog number, clone number, OR RRID. |  | N/A |
| Primary cultures: Provide species, strain, sex of origin, genetic modification status. |  | N/A |
|  |  |  |
| **Experimental animals** | **Indicate where provided: section/figure legend** | **N/A** |
| Laboratory animals or Model organisms: Provide species, strain, sex, age, genetic modification status. Provide accession number in repository OR supplier name, catalog number, clone number, OR RRID. | Methods, "Fly stocks" (Drosophila melanogaster; Bloomington/Kyoto stock numbers and donor laboratories) and "Fly husbandry" (male flies, 2–5 days post-eclosion, group-housed, 25 °C; both sexes in Supplementary Fig. 4h, i). Genotype and sample size for every panel: Supplementary Data 1. Key Resources Table. |  |
| Animal observed in or captured from the field: Provide species, sex, and age where possible. |  | N/A |
|  |  |  |
| **Plants and microbes** | **Indicate where provided: section/figure legend** | **N/A** |
| Plants: provide species and strain, ecotype and cultivar where relevant, unique accession number if available, and source (including location for collected wild specimens). |  | N/A |
| Microbes: provide species and strain, unique accession number if available, and source. |  | N/A |
|  |  |  |
| **Human research participants** | **Indicate where provided: section/figure legend) or state if these demographics were not collected** | **N/A** |
| If collected and within the bounds of privacy constraints report on age, sex, gender and ethnicity for all study participants. |  | N/A |

**Design:**

| **Study protocol** | **Indicate where provided: section/figure legend** | **N/A** |
| --- | --- | --- |
| If the study protocol has been pre-registered, provide DOI. For clinical trials, provide the trial registration number OR cite DOI. | Study not pre-registered. | N/A |
|  |  |  |
| **Laboratory protocol** | **Indicate where provided: section/figure legend** | **N/A** |
| Provide DOI OR other citation details if detailed step-by-step protocols are available. | No separate step-by-step protocol deposited; full procedures are given in Methods ("Heartbeat and behavior recording", "Heartbeat (without behavior) recording", "Mechanical stimulation", "Optogenetic activation of DA-WED neurons", "Optogenetic cardiac deceleration", "Immunohistochemistry", "Calcium imaging", "Surgery", "Feeding preference test"). | N/A |
|  |  |  |
| **Experimental study design (statistics details) *** | | |
| **For in vivo studies: State whether and how the following have been done** | **Indicate where provided: section/figure legend. If it could have been done, but was not, write “not done”** | **N/A** |
| Sample size determination | Methods, "Statistics": sample sizes were not predetermined; n per group in Supplementary Data 1. |  |
| Randomisation | Not done (Methods, "Statistics"). |  |
| Blinding | Not done (Methods, "Statistics"). |  |
| Inclusion/exclusion criteria | Methods, "Feeding preference test" (flies with insufficiently coloured abdomen excluded as poorly fed). No other inclusion/exclusion criteria were applied. |  |
|  |  |  |
| **Sample definition and in-laboratory replication** | **Indicate where provided: section/figure legend** | **N/A** |
| State number of times the experiment was replicated in the laboratory. | Supplementary Data 1 and figure legends: n = number of flies (calcium imaging: number of cells and flies). Each fly received 10 puff trials (Methods, "Heartbeat and behavior recording") or 2 trials (Methods, "Heartbeat (without behavior) recording"), averaged within fly. |  |
| Define whether data describe technical or biological replicates. | Biological replicates: n denotes individual flies (or cells, for calcium imaging); repeated trials within a fly are technical replicates and were averaged per fly before statistics (Methods, "Automatic heartbeat detection", "Automatic behavioral identification"). |  |
|  |  |  |
| **Ethics** | **Indicate where provided: section/submission form** | **N/A** |
| Studies involving human participants: State details of authority granting ethics approval (IRB or equivalent committee(s), provide reference number for approval. |  | N/A |
| Studies involving experimental animals: State details of authority granting ethics approval (IRB or equivalent committee(s), provide reference number for approval. | Submission form. Experiments used Drosophila melanogaster (invertebrate); no institutional ethical approval is required. |  |
| Studies involving specimen and field samples: State if relevant permits obtained, provide details of authority approving study; if none were required, explain why. |  | N/A |
|  |  |  |
| **Dual Use Research of Concern (DURC)** | **Indicate where provided: section/submission form** | **N/A** |
| If study is subject to dual use research of concern regulations, state the authority granting approval and reference number for the regulatory approval. |  | N/A |

**Analysis:**

| **Attrition** | **Indicate where provided: section/figure legend** | **N/A** |
| --- | --- | --- |
| Describe whether exclusion criteria were pre-established. Report if sample or data points were omitted from analysis. If yes, report if this was due to attrition or intentional exclusion and provide justification. | Methods, "Statistics" and "Feeding preference test"; n per group in Supplementary Data 1. Only pre-defined exclusion: poorly fed flies in the feeding preference test. No other samples or data points were omitted. |  |
|  |  |  |
| **Statistics** | **Indicate where provided: section/figure legend** | **N/A** |
| Describe statistical tests used and justify choice of tests. | Methods, "Statistics": Shapiro–Wilk test for normality; Welch's t-test or Dunnett's test when normal, otherwise Wilcoxon signed-rank / rank-sum or Dunn's test; all two-sided; Bonferroni correction for two tests, built-in correction for Dunnett/Dunn. Tests, n and p values in figure legends; full results in Supplementary Data 2. |  |
|  |  |  |
| **Data availability** | **Indicate where provided: section/submission form** | **N/A** |
| For newly created and reused datasets, the manuscript includes a data availability statement that provides details for access (or notes restrictions on access). | "Data availability" section; Source Data file (numerical data for every main and supplementary figure panel) and Supplementary Data 1–2 are uploaded with the article. |  |
| When newly created datasets are publicly available, provide accession number in repository OR DOI and licensing details where available. | Source Data uploaded with the article (eLife); no external repository deposition. |  |
| If reused data is publicly available provide accession number in repository OR DOI, OR URL, OR citation. |  | N/A |
|  |  |  |
| **Code availability** | **Indicate where provided: section/figure legend** | **N/A** |
| For any computer code/software/mathematical algorithms essential for replicating the main findings of the study, whether newly generated or re-used, the manuscript includes a data availability statement that provides details for access or notes restrictions. | "Code availability" section: custom Python and R code (heartbeat detection, SVM behaviour classifier, analysis) at https://github.com/mtsuji172/heart_behavior. |  |
| Where newly generated code is publicly available, provide accession number in repository, OR DOI OR URL and licensing details where available. State any restrictions on code availability or accessibility. | https://github.com/mtsuji172/heart_behavior. |  |
| If reused code is publicly available provide accession number in repository OR DOI OR URL, OR citation. | SLEAP (Pereira et al., 2022; RRID:SCR_021382), ImageJ (NIH), R and Python — Methods, "Automatic behavioral identification", "Immunohistochemistry", "Data analyses". |  |

**Reporting:**

The MDAR framework recommends adoption of discipline-specific guidelines, established and endorsed through community initiatives.

| **Adherence to community standards** | **Indicate where provided: section/figure legend** | **N/A** |
| --- | --- | --- |
| State if relevant guidelines (e.g., ICMJE, MIBBI, ARRIVE, STRANGE) have been followed, and whether a checklist (e.g., CONSORT, PRISMA, ARRIVE) is provided with the manuscript. | No discipline-specific reporting guideline applies (invertebrate study; ARRIVE/STRANGE not applicable). This MDAR checklist is provided. |  |

* We provide the following guidance regarding transparent reporting and statistics; we also refer authors to [Ten common statistical mistakes to watch out for when writing or reviewing a manuscript](https://doi.org/10.7554/eLife.48175).

**Sample-size estimation**

- You should state whether an appropriate sample size was computed when the study was being designed
- You should state the statistical method of sample size computation and any required assumptions
- If no explicit power analysis was used, you should describe how you decided what sample (replicate) size (number) to use

**Replicates**

- You should report how often each experiment was performed
- You should include a definition of biological versus technical replication
- The data obtained should be provided and sufficient information should be provided to indicate the number of independent biological and/or technical replicates
- If you encountered any outliers, you should describe how these were handled
- Criteria for exclusion/inclusion of data should be clearly stated
- High-throughput sequence data should be uploaded before submission, with a private link for reviewers provided (these are available from both GEO and ArrayExpress)

**Statistical reporting**

- Statistical analysis methods should be described and justified
- Raw data should be presented in figures whenever informative to do so (typically when N per group is less than 10)
- For each experiment, you should identify the statistical tests used, exact values of N, definitions of center, methods of multiple test correction, and dispersion and precision measures (e.g., mean, median, SD, SEM, confidence intervals; and, for the major substantive results, a measure of effect size (e.g., Pearson's r, Cohen's d)
- Report exact p-values wherever possible alongside the summary statistics and 95% confidence intervals. These should be reported for all key questions and not only when the p-value is less than 0.05.

**Group allocation**

- Indicate how samples were allocated into experimental groups (in the case of clinical studies, please specify allocation to treatment method); if randomization was used, please also state if restricted randomization was applied
- Indicate if masking was used during group allocation, data collection and/or data analysis
