# Supplementary material for: Dopaminergic neurons linking threat processing to cardiac modulation and locomotor responses
Source: eLife. 2026 Nov 5;15:RP111256. doi: 10.7554/eLife.111256 (PMC13739268; doi:10.7554/eLife.111256)
Supplement: Peer review history

# Editor report: eLife Assessment

**DOI:** 10.7554/eLife.111256.3.sa0

**Authors:** Hiromu Tanimoto

**Affiliations:** Tohoku University Japan

**Keywords:** Convincing, Important

This **important** study identifies two pairs of dopaminergic neurons (DA-WED) in *Drosophila* that coordinate cardiac deceleration and locomotor responses to a mechanical threat. The conclusions are supported by **convincing** evidence from a combination of optogenetic, physiological, and behavioral experiments. The work provides invaluable insights into the neural coordination of behavioral and cardiac responses to threat and will be broad interest to neuroscientists, physiologists, and researchers studying brain-body interactions.

# Referee report: Reviewer #1 (Public review)

**DOI:** 10.7554/eLife.111256.3.sa1

Summary:

This study by Tsuji et al. explores a mechanical threat model in *Drosophila* using air puffs as a stimulus. The authors first establish the paradigm and show that air puffs induce cardiac deceleration along with increased locomotion. They then identify dopamine as a key regulator of this response and go on to map the underlying circuit. In doing so, they pinpoint two pairs of DA-WED neurons as critical players. They carefully used intersectional strategies to achieve relatively clean labeling of these neurons, which helps ensure that the observed effects can be attributed specifically to DA-WED neurons. They further show that DA-WED neurons are both required and sufficient to drive cardiac deceleration, and that their activity increases in response to air puff stimulation. These neurons also contribute to the locomotor response. Directly inducing cardiac deceleration via optogenetic manipulation of cardiomyocytes also increases locomotion, suggesting a link between cardiac state and behavioral output.

Strengths:

Overall, the experiments are thoughtfully designed, well controlled, and clearly presented. The figures are easy to follow, and the conclusions are generally well supported by the data. The manuscript is also clearly written, with a discussion that acknowledges potential caveats and outlines future directions. The genetic tools, behavioral paradigm, heart rate measurement approaches, and stimulation methods introduced here will be valuable resources for the community. few minor comments:

Comments on revised version:

I thank the authors for responding to my questions, they have thoroughly addressed my comments. I am therefore supportive of publication of the manuscript.

# Referee report: Reviewer #2 (Public review)

**DOI:** 10.7554/eLife.111256.3.sa2

Summary:

The authors study cardiac deceleration during threat responses in *Drosophila*. Particularly, it focuses on identifying the neuronal control of this deceleration. Using behavioral and cardiac tracking and analysis, genetics and calcium imaging, they identify two pairs of dopaminergic neurons involved in cardiac deceleration during air puff responses.

Strengths:

The study is overall well done and the paper clearly written. Particularly the work on identifying the two pairs of dopaminergic neurons involved in cardiac deceleration using a series of drivers and generating new ones is rigorous and extensive. Finally, the authors manipulate heartbeat to investigate how it influences threat responses. Although the evidence that heartbeat deceleration-induced increase in locomotion remains partial, the study reveals important insight in the relationship between locomotion, cardiac activity and neuromodulatory system during threat responses.

# Author comment: Author response

**DOI:** 10.7554/eLife.111256.3.sa3

**Authors:** Masato Tsuji, Daisuke Jinkoma, Yuki Uemura, Ayako Ogasawara, Kazuo Emoto

**Affiliations:** The University of Tokyo Tokyo Japan; The University of Tokyo Tokyo Japan; The University of Tokyo Tokyo Japan; The University of Tokyo Tokyo Japan; The University of Tokyo Tokyo Japan

The following is the authors’ response to the original reviews.

**Public Reviews:**

**Reviewer #1 (Public review):**

Summary:

This study by Tsuji et al. explores a mechanical threat model in *Drosophila* using air puffs as a stimulus. The authors first establish the paradigm and show that air puffs induce cardiac deceleration along with increased locomotion. They then identify dopamine as a key regulator of this response and go on to map the underlying circuit. In doing so, they pinpoint two pairs of DA-WED neurons as critical players. They carefully used intersectional strategies to achieve relatively clean labeling of these neurons, which helps ensure that the observed effects can be attributed specifically to DA-WED neurons. They further show that DA-WED neurons are both required and sufficient to drive cardiac deceleration, and that their activity increases in response to air puff stimulation. These neurons also contribute to the locomotor response. Directly inducing cardiac deceleration via optogenetic manipulation of cardiomyocytes also increases locomotion, suggesting a link between cardiac state and behavioral output.

Strengths:

Overall, the experiments are thoughtfully designed, well-controlled, and clearly presented. The figures are easy to follow, and the conclusions are generally well supported by the data. The manuscript is also clearly written, with a discussion that acknowledges potential caveats and outlines future directions. The genetic tools, behavioral paradigm, heart rate measurement approaches, and stimulation methods introduced here will be valuable resources for the community.

Weaknesses:

A few minor points to add to the clarity of the manuscript:

(1) The DA-WED driver (R48A08-AD ∩ VT008692-DBD ∩ TH-FLP) appears quite clean in the brain. However, since the study focuses on cardiac function and locomotion, it would be helpful to check expression in cardiomyocytes and the ventral nerve cord. This would help rule out any off-target expression that might contribute to the phenotypes and further support the idea of a descending pathway from brain dopaminergic neurons.

We thank reviewer#1 for this thoughtful comment. We have indeed checked the expression pattern of the DA-WED driver (R48A08-AD ∩ VT008692-DBD ∩ TH-FLP) throughout the body, and failed to detect noticeable expression outside the brain (Supplementary Fig. 6d in the original manuscript). As for the heart, the whole tissue was cleared and confocal-imaged from the dorsal side. As cardiomyocytes are positioned directly beneath the dorsal cuticle of the abdomen, the expression of a driver can be best assessed this way. In contrast to the lack of apparent expression in cardiomyocytes, we could see clear expression in DA-WED neurons in the brain in the same sample (Supplementary Fig. 6d). In this experiment, however, potential expression in VNC could have been missed, as VNC is positioned on the ventral side and thus further away from the objective lens. We thus conducted an additional experiment in which we dissected out the brain + VNC and checked the driver’s expression. Our confocal imaging results revealed no expression in VNC. We have included this data in our updated manuscript as Supplementary Fig. 6e.

(2) Since DA-WED>Kir2.1 abolishes the puff-induced locomotor response (Figure 5b), suggesting that DA-WED neurons are directly involved in mediating locomotion. In the model (Figure 5L), it might make more sense for the pathway from mechanical threat to locomotion to pass through DA-WED neurons. The authors could consider adjusting the schematic if they agree.

As the reviewer pointed out, Kir2.1-mediated suppression of DA-WED neurons almost completely abolished puff-induced locomotion. In our original schematic, we included a pathway from mechanical threat to locomotion that bypassed DA-WED, reflecting the possibility that locomotion could be driven by multiple pathways. We agree, however, that this possibility was not directly supported by our data and that the near-complete suppression of puff-induced locomotion upon DA-WED silencing more strongly supports a model in which the predominant flow of information from puff detection to locomotion passes through DA-WED neurons. We have therefore removed the direct arrow from mechanical threat to locomotion and added a dashed arrow from DA-WED neurons to locomotion, representing a pathway that may promote locomotion independently of DA-WED-mediated cardiac deceleration (Fig. 5m; Supplementary Fig. 9i).

We have updated the legend of Fig. 5m as follows:

“Model. DA-WED neurons promote locomotion, through and/or bypassing interoception of the heartbeat (dashed arrows).”

We also updated the legend of Supplementary Fig. 9i as follows:

“Model. Air puff promotes locomotion while suppressing grooming. Mechanistically, air puff activates DA-WED neurons. DA-WED neurons drive not only cardiac deceleration but locomotion, potentially through a feedback from cardiac deceleration (dashed arrows). Such a DA-WED-based mechanism fails to influence grooming.”

(3) In line 408, Figure 5K should be 5L as it's a discussion of the model.

We thank reviewer#1 for pointing this out. Accordingly, we have made the following corrections:

L436-439: “cardiac deceleration is accompanied by increased locomotion (Fig. 5e-l). Our data points to a scenario in which DA-WED neurons induce cardiac deceleration upon mechanical threat, which may in turn influence threat-induced locomotion (Fig. 5m).”

(4) In Figure 5j, the x-axis is missing time labels. Even if it matches Figure 5h, adding labels would make it easier to interpret at a glance.

We agree with reviewer#1 and added the x labels to Fig. 5j, and also to the second panels of h, j, and l.

(5) In line 312, it would be helpful to briefly explain why a 28 ms light pulse was used, compared to other pulse durations elsewhere in the paper.

Our system synchronized behavior, heartbeat, and optogenetics at 70 Hz (14-ms intervals). Because mechanical threat transiently elongated beat-to-beat intervals typically by 20 - 23ms, we chose 28-ms (2-frame) stimulation to best mimic this effect, over 14 (1-frame) or 42 (3-frame) ms (Fig. 1; Supplementary Fig. 2b, e, f). We have added this explanation to our manuscript, as follows:

L326 - 328: “To approximate the 20–23 ms beat-to-beat prolongation evoked by mechanical threat (Fig. 1; Supplementary Fig. 2e, f), we used 28-ms (2-frame) green light pulses.”

(6) The cardiac deceleration seems to recover quickly after the air puff ends, whereas the locomotor response persists longer (around 10-15 seconds; see Figure 1 and Figure 5). This difference might suggest that DA-WED neurons influence locomotion through an additional or partially independent pathway, beyond their role in cardiac regulation. It could be worth briefly discussing this possibility.

We agree that the time course of puff-induced locomotion (Fig. 1f, Fig. 5a) is more persistent than DA-WED activation (Fig. 4f) or cardiac deceleration (Fig. 1d, Fig. 4b). Also, silencing DA-WED neurons partially reduced puff-induced cardiac deceleration (Fig. 4c) but appears to have more strongly abolished puff-induced locomotion (Fig. 5a, b). Given that our interoception model is only partially tested, we agree that it is better to mention the possibility that DA-WED neurons might influence locomotion through an additional or partially independent pathway beyond their role in cardiac regulation.

Our “Limitation of this study” section in our original manuscript touched on this issue, as follows (L430-432):

“Although cardiac deceleration can promote locomotion, we have not directly tested whether it is required for DA-WED–evoked locomotion, leaving open the possibility of parallel downstream pathways.”

But to be more explicit, in our updated manuscript, we also included the following discussion:

L439-441: “We note, however, that our data does not exclude the possibility that DA-WED neurons might influence locomotion at least partially independent of its role in cardiac deceleration”

**Reviewer #2 (Public review):**

Summary:

The authors study cardiac deceleration during threat responses in *Drosophila*. Particularly, it focuses on identifying the neuronal control of this deceleration. Using behavioral and cardiac tracking and analysis, genetics, and calcium imaging, they identify two pairs of dopaminergic neurons involved in cardiac deceleration during air puff responses

Strengths:

The study is overall well done, and the paper is clearly written. Particularly, the work on identifying the two pairs of dopaminergic neurons involved in cardiac deceleration using a series of drivers and generating new ones is rigorous and extensive. Finally, the authors manipulate the heartbeat to investigate how it influences threat responses

Weaknesses:

There are, however, several points that need to be clarified, as some claims are not entirely supported by evidence.

The authors, for example, claim that dopaminergic neurons are responsible for cardiac deceleration (during the air puff, lines 182-3, page 9). However, based on the work in this study, it seems that other neurons could be involved in this control as well. In addition to dopaminergic neurons, the authors test serotonergic and octopaminergic neurons, which, based on silencing experiments, also show an implication in heart-beat deceleration. Furthermore, because they find that dopaminergic neurons are the only ones that, upon thermogenetic activation, lead to lower heart beat frequency, they conclude that the dopaminergic neurons are responsible for air -puff induced cardiac deceleration.

However, these activation experiments are done in a different context than the air puff experiments (at a higher temperature, which could have an effect on the heartbeat changes upon activation of different neuron groups), and because silencing of other monoaminergic neuron types during the air puff also resulted in less cardiac deceleration, one cannot exclude the implication of octopaminergic or serotonergic neurons in air-puff-induced deceleration.

Activation experiments without high temperatures (using, for example, optogenetics) and/or in the presence of the air puff would be important to determine that the dopaminergic neurons are the main type of monoaminergic neurons involved in air-puff-induced cardiac deceleration. Otherwise, the related claims should be rephrased in a way that clearly doesn't exclude a possible implication of other monoaminergic neurons.

We thank reviewer#2 for raising an important point. Our intention was not to claim that dopaminergic system is solely responsible for puff-induced cardiac deceleration. What we meant to convey in our original manuscript was that, after conducting experiments shown in Fig. 2, dopaminergic system was the one that made most sense to focus on, as the other system failed to show consistent results across loss- and gain-of-function experiments. We acknowledge, as reviewer #2 rightly pointed out, that consistency across loss- and gain-of-function experiments is not a logical necessity for given neurons to be causally contributing to a brain function, especially in this case where loss- and gain- of-function experiments were conducted at different temperatures. We have revised our manuscript to more precisely express our view outlined above, as follows:

L194-196: “Together, our data demonstrates that, while not excluding the contributions of serotonergic or octopaminergic systems, dopamine system is responsible for puff-induced cardiac deceleration.”

Regarding the interactions between the cardiac deceleration and locomotion, the authors propose, based on the results, that the optogenetic cardiac deceleration is sufficient to induce an increase in locomotion, and that it is the decrease in heartbeat that would be responsible via interoceptive pathways to trigger an increase in locomotion. In the model they propose, the DA-WED neurons would induce a decrease in heartbeat that, in turn, would trigger an increase in locomotion. There is not enough proof that cardiac deceleration is the one that triggers an increase in locomotion during air puff responses. As the authors themselves state, the experiments that would demonstrate this would involve preventing cardiac deceleration while optogenetically activating DA-WED. It can therefore not be excluded that the DA-WED neurons trigger an increase in locomotion that is possibly modulated by the cardiac activity. Both alternatives should be considered (models in Figures 4 and 5).

We thank the reviewer for this insightful comment, which is also consistent with reviewer#1’s view. We agree that it is better to mention the possibility that DA-WED neurons might influence locomotion through an additional or partially independent pathway beyond their role in cardiac regulation, which our data does not exclude. In our updated manuscript, we included the discussion as below:

L433-441: “Our results provide partial support for such a model. We show that (1) mechanical threat induces cardiac deceleration and locomotion (Fig. 1), (2) DA-WED neurons in the brain are responsible for threat-induced cardiac deceleration and locomotion (Fig. 4; Fig. 5a-d), (3) optogenetic cardiac deceleration is accompanied by increased locomotion (Fig. 5e-l). Our data points to a scenario in which DA-WED neurons induce cardiac deceleration upon mechanical threat, which may in turn influence threat-induced locomotion (Fig. 5m). We note, however, that our data does not exclude the possibility that DA-WED neurons might influence locomotion at least partially independent of its role in cardiac deceleration.”

**Reviewer #3 (Public review):**

Summary:

In this elegant study, Tsuji et al. identify a relationship in *Drosophila* between cardiodynamics and threatening stimuli where mild air puffs elicit a brief bradycardia that coincides with locomotion increases. They then take advantage of the arsenal of genetic tools available in the fruit fly to reveal the indispensability of dopamine, through the action of Dop1R2, in this phenomenon. Further, they pinpoint the source of this dopamine to two specific pairs of neurons - DA-WED that are threat-activated. They then test and find a potential role for cardiac interoception from the heart in linking behavior and cardiodynamics.

Strengths:

This is an interesting and timely story that brings together the tools of fruit fly systems neuroscience and links it with physiology. The experiments are well done and tell a very nice story. In particular, the primary message of the paper - that the authors have identified specific dopaminergic neurons that regulate cardiac activity - is sound.

Weaknesses:

There are no important problems with the scientific approach. Rather, there are some interpretive changes I would consider.

(1) The changes in heart rate are small (10% or so), and, as far as I can tell, are evident for a beat or two. So the data may be better interpreted not as a change in rate but as a lengthening of diastole for a beat or two. That may seem a petty difference, but it might point to particular stretch-activated systems or changes in blood flow as the determinant.

Heart rate must be averaged over time, and so might be blurring the effects. It may be useful to produce figures centered on beat count and duration rather than time. Because the effect may even be just on a single beat, we suggest the authors try plotting the average beat duration for each beat that follows the air puff. If it's really just the first beat, using a quantification of the change of this duration relative to the average that precedes the puff may produce more striking figures.

We thank reviewer#3 for this insightful suggestion, which also relates to the reviewer’s next comment. We tried plotting the average beat duration for each beat that follows the air puff, relative to beats preceding the puff. The reviewer’s prediction turned out to be partially true – our plot of beat-to-beat interval vs beat count around puff revealed that the interval indeed increased for the three beats following puff across different puff strengths (Supplementary Fig. 2e). Consistently, the average interval over three beats before and after puff per fly revealed larger differences for 50mL/min and especially 200mL/min but no noticeable changes for 20mL/min, so that now we detect similar cardiac deceleration across puff strength (Supplementary Fig. 2f). However, this novel analysis unfortunately came with slightly larger variance overall (Supplementary Fig. 2f). We thus prefer to stick to HR in the present manuscript, but still agree that this is valuable for a more comprehensive understanding of cardiac puff responses. We included our new plot as Supplementary Fig. 2e, f, with the following description in the Result section:

L121-122: “Indeed, the beat-to-beat interval increased over the three beats following puff onset, and this increase was comparable across puff strengths (Supplementary Fig. 2e, f).”

(2) The author's model that cardiac deceleration leads to walking data is only partially supported by their data. In the first figure, the relationship between cardiac deceleration and walking probability seems to be inverted relative to their model (weak stimulus -> strong cardiac effect and weak locomotor effect; strong stimulus-> weak cardiac effect and strong locomotor effect). It is possible that this discrepancy may disappear when the authors look at beat duration rather than heart rate (for instance, if following the strong stimulus, there is a very long beat that is followed by tachycardia, thus weakening their observed HR change). It would also be easier to relate this data in Figure 1 to their interoceptive model if some data were shown that illustrated the relative timing of the cardiac change and the locomotor start.

Following the reviewer's suggestion, we re-examined the cardiac response using the beat-to-beat interval rather than heart rate. As the reviewer anticipated, this partially resolves the apparent discrepancy. Averaging the interval over the three beats before and after the puff (per fly) reveals a significant increase in beat-to-beat interval at every puff strength, with comparable magnitude across strengths (Supplementary Fig. 2e, f). However, since a comparable cardiac response, in terms of beat-to-beat interval, is accompanied by a graded locomotor response, locomotion cannot be driven by cardiac deceleration alone. Indeed, puff-induced cardiac deceleration and locomotion fail to correlate (added as Supplementary Fig. 10a in our updated manuscript). In contrast, optogenetic cardiac deceleration does increase locomotion (Fig. 5h-k), and with a strong correlation between optogenetically activated cardiac deceleration and locomotion (added as Fig. 5l in our updated manuscript). Taken together, locomotion is likely driven by both cardiac deceleration and a parallel, cardiac-independent pathway, with the latter accounting for the puff-strength dependence (Fig. 5m). We added the following description in the Result section:

L121-122: “Indeed, the beat-to-beat interval increased over the three beats following puff onset, and this increase was comparable across puff strengths (Supplementary Fig. 2e, f).”

Regarding the relative timing, we added a dedicated figure as Fig. 1h, which shows that the onsets of the cardiac and locomotor changes are approximately concurrent across puff strengths. They differ in their peak timing — the cardiac deceleration reaches its extremum in roughly 0.2 – 0.5 s, while the walking fraction peaks later (roughly 1 – 1.5 s). We have added the following to the Result section:

L127-139: “A close inspection of the peri-stimulus dynamics revealed that the onsets of the cardiac and locomotor changes were approximately concurrent at puff onset across strengths (Fig. 1h). Furthermore, the peak of cardiac deceleration (roughly 0.2 – 0.5 s post puff onset) preceded that of locomotor increase (roughly 1 – 1.5 s post puff onset). Especially at lower puff strengths, locomotor response showed an additional transient dip, embedded in dominant and persistent locomotion, almost concurrently, but in the case of 50 mL/min following, cardiac deceleration (Fig. 1h). Overall, cardiac and locomotor responses begin together, and the cardiac response reaches its peak before the locomotor response does. These data are consistent with the idea that cardiac and locomotor responses are coordinated, and that cardiac deceleration may causally contribute to the locomotor response. We therefore set out to identify the neurons responsible for cardiac deceleration and to test their causal role directly.”

We have also updated our corresponding Discussion section (chapter 1) accordingly:

L369-389: “Cardiac deceleration is coordinated with defensive locomotion

Our data show that air puff stimulation induces cardiac deceleration together with increased locomotion (Fig. 1). Across puff strengths, locomotion scaled with stimulus strength, whereas the cardiac deceleration did not: measured as time-binned HR, it was less pronounced at stronger puffs, while measured as the beat-to-beat interval it was comparable across strengths (Fig. 1d–h; Supplementary Fig. 2f). This indicates that locomotion is not simply a graded readout of cardiac deceleration.

Although cardiac deceleration is often associated with freezing in mammals [1], cardiac deceleration has also been observed during early threat detection and orienting responses [78], suggesting that it reflects engagement of a defensive processing rather than motor inhibition per se. Consistent with this view, dissociation between threat-evoked cardiac modulation and freezing has been reported across taxa. In mammals, for example, disruption of autonomic output can abolish cardiac deceleration without eliminating freezing [79]. Similarly, in decapod crustaceans and *Drosophila*, visual stimuli elicit reproducible and time-locked changes in heart rate that accompany both freezing and fleeing [8,9,80]. While these studies establish that cardiac modulation can be dissociated from freezing, our findings additionally demonstrate that cardiac deceleration can co-occur with a predominantly locomotor response to mechanical threat and can itself causally contribute to locomotion (Fig. 5e-m). Cardiac deceleration is therefore not restricted to passive defensive states, but can act upstream of active locomotor responses.”

We also examined HR-versus-locomotion relationships trial-by-trial and fly-by-fly, but trial-to-trial fluctuations in both signals were too large to pinpoint the timing of changes at that level, which is why we present the population peri-stimulus dynamics in Fig. 1h.

(3) Also, since the locomotor and cardiac changes are probabilistic, it would be very useful to see how their respective probabilities change when conditioned on the other. According to their interoceptive model, locomotion should preferentially increase on trials where cardiac deceleration occurs. The authors should discuss this incongruity and also potential alternative interpretations of their cardiac manipulation experiments. Perhaps the bradycardia makes them more sensitive to threats - as suggested in the introduction? Control flies show a mild increase in locomotion following green light (Figure 5j), so perhaps by slowing the heart, they are more sensitive and thus respond more strongly to this stimulus?

We believe that cardiac and locomotor changes are predicted to be congruent across flies only if locomotion is solely dependent on cardiac changes. Our optogenetic cardiac deceleration experiment likely corresponds to such a situation, and we indeed observed a strong correlation between cardiac and locomotor changes for retinal-fed test group but not for retinal-unfed control group (which we now present as Fig. 5l).

As for stimulus-induced locomotion, it may reflect contributions from cardiac-state-independent locomotor pathways (Fig. 5m). In such a situation, cardiac and locomotor changes are not necessarily predicted to be congruent. Indeed, puff-induced locomotion and cardiac changes do not appear to be congruent across flies (which we now present as Supplementary Fig. 10a).

We have added these plots as Fig. 5l and Supplementary Fig. 10a, and added the following explanation in the main text:

L335 - 341: “Furthermore, optogenetically induced changes in HR and locomotion were strongly anti-correlated fly-wise in the retinal-fed test group (Pearson’s correlation coefficient: -0.81, p-value: 0.00538) but not in the control group without retinal (Pearson’s correlation coefficient: 0.43, p-value: 0.436) (Fig. 5l). This contrasts with puff-induced responses, in which changes in HR and locomotion were not significantly correlated (Supplementary Fig. 10a). This lack of correlation may reflect contributions from cardiac-state-independent locomotor pathways.”

As for the possibility of bradycardia promoting locomotor response to threats (light), We thank the reviewer for raising this alternative interpretation. We agree that our experiment does not distinguish whether cardiac deceleration directly promotes locomotion or instead enhances the locomotor response to the green-light stimulus itself. Indeed, retinal-unfed control flies showed a modest increase in locomotion following green-light illumination, indicating that the light can itself influence behavior. Thus, the larger locomotor response in retinal-fed flies could in principle reflect an interaction between the optogenetically induced cardiac state and this sensory response.

We note, however, that this interpretation would still imply a causal influence of cardiac state on behavior: cardiac deceleration would increase the gain of an externally evoked locomotor response rather than directly initiating locomotion. Because our experiment does not discriminate between these possibilities, we have revised our manuscript to state that experimentally induced cardiac deceleration can promote locomotion or potentiate a light-evoked locomotion.

L346–L350: "We also note that retinal-unfed controls showed a modest locomotor increase following green-light illumination (Fig. 5j, k), leaving open whether cardiac deceleration directly promotes locomotion or potentiates a light-evoked locomotion. In either case, our data support a modulatory influence of cardiac state on locomotor output.”

We thank the reviewer for significantly strengthening our manuscript.

(4) Looking at the example shapes of the beats in Figure 5g versus Figure 1c, the optogenetically induced diastole has a very different shape from the naturally occurring long beat. Thus, the exact cardiac stimulus may be unnatural. If this is true across trials and animals, it may be worth considering that the funny beat (like an anxiogenic atrial fibrillation in mammals) is the source of the fear and, in turn, locomotor behavior (also interesting!) rather than being a true replication of the cardiac events seen following the puff stimulus.

We thank the reviewer for this insightful comment. We agree that the optogenetically induced cardiac deceleration does not fully reproduce every feature of the puff-induced heartbeat, and we have been careful not to claim that it does. Our aim was to test whether cardiac deceleration can influence locomotor state, not to claim that the optogenetically induced beat is an exact replica of the puff-induced response.

At the level of HR, the two manipulations were closely matched: both produced an ~10% reduction with a similar time course. They differed in the finer beat-to-beat structure — the optogenetic deceleration was concentrated in a transient prolongation at stimulus onset, whereas the puff-induced deceleration was distributed across several beats (Supplementary Fig. 10). In both cases, however, the M-mode recordings show that the heart maintained an organized rhythm, with the manipulation producing a transient diastolic prolongation rather than sustained beat-to-beat irregularity (Fig. 1c, Fig. 5g). This argues against the possibility that a fibrillation-like funny beat underlies the behavioral effect, but leaves open the possibility of light-induced one long interval, vs puff-induced long intervals across three consecutive beats, may have contributed to behavior. We also note that our current imaging system does not yet permit a comprehensive comparison of finer heartbeat features, such as full contraction dynamics, waveform shape, stroke volume/ejection fraction, or beat-pattern irregularity quantified across trials and animals. We therefore do not intend to exclude the possibility that specific features of the optogenetically induced beat pattern contribute to the behavioral effect — an interesting question that we agree is worth pursuing.

We have revised the manuscript accordingly. We now state, with an added figure (Supplementary Fig. 10), that the optogenetic experiment supports that experimentally induced cardiac deceleration can modulate locomotor behavior, but does not establish that the artificial cardiac pattern is identical to the mechanically evoked response. We also note the open possibility that distinct heartbeat patterns may themselves influence defensive behavior.

L343–L346: " One limitation is that our optogenetically induced cardiac pattern (Fig. 5g; Supplementary Fig. 10b, c) is not necessarily identical to its mechanically induced counterpart (Fig. 1c; Supplementary Fig. 10d, e), which could have influenced locomotion.”

L321-324: “To directly test whether cardiac deceleration can influence locomotion, we optogenetically induced cardiac deceleration of a magnitude comparable to the puff-induced response by expressing the light-gated anion channel GtACR1 in cardiomyocytes using Hand-GAL4 (Fig. 5e).”

Assessing how heartbeat pattern influences behavior will require improved whole-heart imaging and more detailed quantitative analysis of heartbeat dynamics, which we are currently developing but consider beyond the scope of the present study.

**Recommendations for the authors:**

**Reviewer #2 (Recommendations for the authors):**

Recommendation for the authors:

(1) In the introduction section (around line 43), it would be important to also include relevant work on the relationship between cardiac activity and threat responses in *Drosophila* (Barrios et al, 2021) as this study describes the internal physiological states of fruit flies during defensive responses (including the changes in heartbeat).

We thank the reviewer for pointing this out. The suggested paper was referenced in our original Introduction but slightly later (L78) than the location suggested by the reviewer (L44). We have now included this reference in L44 as well.

(2) Activation experiments could be performed in the presence of an air puff. The authors show that the stronger the air puff rate, the stronger the response. Low or medium air puff could be used during optogenetic activation of serotonergic and octopaminergic neurons, and see if the activation of different neuron types enhances cardiac deceleration and increase of locomotion (to levels similar to those observed, for example, at high puff rates). Otherwise, the claim that the dopaminergic system is solely responsible should be rephrased.

We agree that the suggested experiment can more reliably assess the potential contribution of serotonergic and octopaminergic systems. However, our intention was not to claim that dopaminergic system is solely responsible for puff-induced cardiac deceleration. Rather, consistency across loss- and gain-of-function experiments led us to focus on dopaminergic system, which allowed us to identify DA-WED neurons. We have revised our manuscript to more precisely express our view outlined above, as follows:

L194-196: “Together, our data demonstrates that, while not excluding the contributions of serotonergic or octopaminergic systems, dopamine system is responsible for puff-induced cardiac deceleration.”

(3) Since there is not enough proof that cardiac deceleration is the one that triggers an increase in locomotion during air puff responses and cannot be excluded that the DA-WED neurons trigger an increase in locomotion that is further modulated by the cardiac deceleration. Both options should be considered (models in Figures 4 and 5, results and discussion).

We thank the reviewer for this insightful comment, which is also consistent with reviewer#1’s view. We agree that it is better to mention the possibility that DA-WED neurons might influence locomotion through an additional or partially independent pathway beyond their role in cardiac regulation, which our data does not exclude. In our updated manuscript, we added to our schematic model an arrow emanating from DA-WED to locomotion directly (Fig. 5m), explaining our interpretation in the corresponding legend. We also included the discussion as below:

L439 - 441: “We note, however, that our data does not exclude the possibility that DA-WED neurons might influence locomotion at least partially independent of its role in cardiac deceleration.”

(4) Pages 14-15 (and in discussion). It appears that in some cases, puff and threat are used interchangeably, which may be confusing, as for example the authors ask if DA-WED could be general threat detectors in this context, meaning, if I understand correctly, that they are involved in all the aspects of the behavioral response to an air puff. However, the term general threat detector may be understood to mean not specific to air puff but also other types of threats, which is not addressed in this study. I suggest this be rephrased to avoid confusion. Also, they should be clear in the text when they refer specifically to air puff threats and when to threat responses.

We thank the reviewer for this important clarification. We swapped the phrase “general threat detectors” with “puff detectors” for precision. We have revised our manuscript accordingly, as follows:

L306 - 320: “An alternative interpretation is that DA-WED neurons function as mere puff detectors that broadly regulate puff-evoked behaviors. If this were the case, manipulating these neurons should affect all behavioral outputs induced by puff. In addition to promoting locomotion, air puff suppressed grooming predominantly within 3 s of puff onset, consistently across puff strengths; grooming during the later 10–20 s window was variable, either remaining suppressed, returning to baseline, or reversing to an increase depending on puff strength (Supplementary Fig. 9a–c). However, silencing DA-WED neurons failed to disrupt puff-induced grooming suppression (Supplementary Fig. 9d–f), despite markedly reducing cardiac deceleration (Fig. 4b, c) and locomotion (Fig. 5a, b). Likewise, photoactivation of DA-WED neurons failed to suppress grooming (Supplementary Fig. 9g–i), while robustly inducing cardiac deceleration (Fig. 4i, j) and locomotion (Fig. 5c, d). Thus, manipulation of DA-WED neurons does not reproduce the full behavioral repertoire triggered by puff. These findings argue against the idea that DA-WED neurons function as mere puff detectors and instead support a more specific role in coordinating cardiac and locomotor responses.”

To better distinguish “external threat” vs “threat responses”, we have also made the following changes in our main text:

L303-305: “These findings suggest that DA-WED neurons coordinate cardiac deceleration with locomotor responses upon mechanical threat.”

L341-343: “These observations are consistent with the possibility that cardiac deceleration may influence locomotor responses upon mechanical threat”

L437-439: “Our data points to a scenario in which DA-WED neurons induce cardiac deceleration upon mechanical threat, which may in turn influence threat-induced locomotion (Fig. 5m).”

L477-478: “While our data are consistent with a model in which DA-WED neurons couple cardiac deceleration to locomotor responses upon mechanical threat”

(5) In Supplementary Figure 3, the R74C07 labels anterior and posterior sensilla. Since the external sensilla on the posterior abdomen were excluded based on the Aes1N driver's result, the phenotype is likely due to the anterior sensilla. However, those are not included in the summary in the (i) panel. Why is that?

We thank the reviewer for pointing this out. Our policy was that “When multiple drivers labeled the given neurons, we considered such neurons as indispensable for cardiac deceleration only when those drivers produced consistent results (hence, e.g. external sensilla on the posterior abdomen were excluded based on Aes1N driver's result, despite R74C07 driver's result suggests otherwise)”. Accordingly, sensilla on the eye compound (“anterior” sensilla in reviewer’s comment) labeled by R74C07 driver were excluded by “R38B08AD-VT043775DBD” split combination’s result. Our original legend of Supplementary Fig. 3i explained our policy, but to avoid confusion, our updated legend now includes above explanation as well, as follows:

“(hence, e.g. external sensilla on the posterior abdomen were excluded based on Aes1N driver's result, despite R74C07 driver's result suggests otherwise; similarly, sensilla on the eye compound labeled by R74C07 driver were excluded by “R38B08-AD VT043775-DBD” split combination’s result).”

(6) There seems to be a strong off response to air puff in medial and not in lateral DA-WED neurons, suggesting that the single pair of neurons could have a different function in the context of air puff responses. That should be discussed.

We agree with the reviewer that there may be differences in puff responses in medial vs lateral DA-WED neurons (Supplementary Fig. 8c). We have included this in our Result section as follows:

L281 – 283: “We note, though, that the medial neurons tended to show offset response-like activities more pronounced than their lateral counterparts (Supplementary Fig. 8c), potentially hinting at functional heterogeneity within DA-WED neurons.”

(7) Grooming: In Supplementary Figure 9c, although not significant, the silencing of DA-WED does seem to have an effect on the grooming profile- averaging over the 10-second time window does not reveal a significant effect, but a finer analysis may reveal some differences. There seems to be an increase in grooming after 10s? (in e-h).

We added the 10–20 s window data as Supplementary Fig. 9c, f, i, l. We note, however, that this window falls outside the period of robust puff-evoked grooming suppression. In wild-type flies, air puff suppressed grooming predominantly within 3 s of puff onset, and this suppression was consistent in direction across all puff strengths (Supplementary Fig. 9a, b). Grooming during the 10–20 s window, by contrast, was variable, either remaining suppressed, returning to baseline, or reversing to an increase depending on puff strength (Supplementary Fig. 9a). We therefore regard the 0–3 s window as reflecting the most reliable puff-evoked grooming response. Within this window, we did not detect statistically significant effects of neuronal or cardiac manipulation on the grooming suppression. Independently of this, we also examined the average grooming fraction during the 10–20 s window: the apparent differences there are driven by a few unusually high-magnitude values and are not statistically significant even when those values are retained (Supplementary Fig. 9f, i, l).

We also made the following changes to the Results section (and added Supplementary Fig. 9c, f, i, l):

L308 – 317: "In addition to promoting locomotion, air puff suppressed grooming predominantly within 3 s of puff onset, consistently across puff strengths; grooming during the later 10–20 s window was variable, either remaining suppressed, returning to baseline, or reversing to an increase depending on puff strength (Supplementary Fig. 9a–c). Silencing DA-WED neurons failed to disrupt puff-induced grooming suppression (Supplementary Fig. 9d–f), despite markedly reducing cardiac deceleration (Fig. 4b, c) and locomotion (Fig. 5a, b). Likewise, photoactivation of DA-WED neurons failed to suppress grooming (Supplementary Fig. 9g–i), while robustly inducing cardiac deceleration (Fig. 4b, c) and locomotion (Fig. 5c, d)."

L333 – 334: “In contrast, this manipulation failed to alter grooming behavior (Supplementary Fig. 9j-l).”

We also spotted an inaccurate description in our original legend, which read “Change in grooming fraction, calculated for each fly as the % difference between the mean fraction during the 10 s following stimulation onset and the mean fraction during the 10 s baseline period preceding stimulation.” This was an error, and we have instead used “during the 3 s following stimulation onset” window as we accurately described in our original Method (L579 – 582: “Puff-induced changes in walking and grooming were quantified by comparing the mean fraction during the 10 s and 3 s, respectively, following puff onset with the mean fraction during the 10 s baseline period preceding puff onset.”). We have corrected this error in our updated legend. We apologize for any confusion this error might have caused.

**Reviewer #3 (Recommendations for the authors):**

(1) Showing the model in Figure 4k as puff in parallel leads to locomotor effects independently of DA-WED, which is incongruent with the next figure where they discuss their interoceptive model. It might be useful to present the interoceptive model as a competing model in 4K that will be tested in the following figure.

Our intention there was to graphically summarize the tentative model that was plausible consistent with data presented in Fig.1-4. While we much appreciate the reviewer’s suggestion, we prefer not to present interoception idea in Fig. 4k: since the interoception idea comes from observations in Fig. 5a-d, we fear that presenting interoception model in Fig. 4k could be confusing without looking at Fig. 5a-d. Still, the reviewer’s suggestion gave us an opportunity to revisit our manuscript’s structure and for that we are thankful.

(2) The effect the authors observe is similar to one that has been [1.1] recently published in zebrafish larvae (Herrera et al. 2026 and Hernandez-Nunez et al. 2026), where lengthened heartbeats accompany active avoidance behavior following threats. Interestingly, this effect seems to be particularly conserved amongst smaller animals like fish larvae and insects, where heartbeats may represent a stronger sensory stimulus. It may be worthwhile for the authors to discuss these parallels. Further, some of the difficulties about interpreting direct optogenetic manipulation of cardiac function are discussed and explored in the Herrera et al. paper.

We thank the reviewer for highlighting these studies and this intriguing parallel. We agree the correspondence across distant taxa is striking. In larval zebrafish, cardiac dynamics and behavioral state are coupled following threat (Herrera et al., 2026), and dedicated vagal sensory neurons encode HR, including subsets responsive to cardiac deceleration, through a largely stimulus (including optogenetic cardiac pacing)-invariant population code (Hernandez-Nunez et al., 2025). Together with the demonstration in mice that optogenetically induced tachycardia can drive an anxiety-like state (Hsueh et al., 2023), and our present findings in *Drosophila*, these results suggest that cardiac-interoceptive influence on behavior is a broadly shared feature of nervous systems across taxa.

We find intriguing the reviewer's suggestion that such coupling may be especially relevant in small animals. Indeed, in small animals, the heart may perturb the internal state more globally - in small animals, the per-beat change in physiological state relative to body size may be proportionally larger, rendering each beat a stronger signal rather than being buffered by a large, compartmentalized vasculature. Alternatively, the compactness of small nervous systems could allow an interoceptive cardiac signal to reach motor circuits through few intervening synapses. In *Drosophila*, almost any neuron can reach any other within roughly four synapses (Scheffer et al., 2020; Dorkenwald et al., 2024; Schlegel et al., 2024), although whether synaptic path lengths are shorter in *Drosophila* than in larger brains remains unknown. We note, however, that the demonstration of cardiogenic affective control in mammals (Hsueh et al., 2023) indicates the phenomenon is not restricted to small animals, and so we consider the size question open.

Finally, following the reviewer's pointer to the interpretive considerations raised by Herrera et al., we have added text acknowledging that our optogenetic manipulation may engage the cardiac state through multiple routes. The term interoception is used broadly to include neural as well as humoral, blood pressure, or gas-exchange-related sensing of the cardiac state, and we note that distinguishing among these routes would require targeted manipulations we have not performed.

We have added the following section to our Discussion:

L453 – 474: “ Cardiac dynamics can modulate behavior

Our findings echo a growing body of work linking cardiac dynamics to behavior across distant taxa. In larval zebrafish, cardiac dynamics and behavior are coupled during threat [85], and dedicated vagal sensory neurons encode HR, including its decreases, through a stimulus-invariant population code86. In mice, optogenetically induced tachycardia can drive anxiety-like behavior [87]. And here we find that cardiac deceleration accompanies, and can promote, locomotion in *Drosophila*. Cardiac influence on behavior thus spans insects, fish, and mammals.

How cardiac state might be sensed in *Drosophila* is unknown, and several routes are conceivable. *Drosophila* heart receives rich neuronal innervation [51,52], although whether these neurons relay sensory information from the heart is unknown. If so, neurons could directly detect the heartbeat, analogous to the cardiac afferents that mediate cardiac interoception in vertebrates [86,87]. Alternatively, the heartbeat could be sensed mechanically or through gas-exchange-related cues. In mammals, the cardiac pressure pulse can be detected directly within the brain by mechanosensitive central neurons88. In zebrafish, cardiac activity can causally influence behavior through its oxygen supply [85]. In insects, although the circulatory architecture differs, the heartbeat contributes to pressure changes in the hemocoel and tracheal system and resultant gas exchange [89,90], which could in principle be detected. While present study does not distinguish among these possibilities, establishing how cardiac state is sensed would provide a foundation for dissecting how central commands and peripheral physiological states interact to coordinate survival behaviors.

(3) Serotonergic inhibition or knockout seems to diminish cardiac deceleration (lines 159-162), yet activation leads to increased heart rate (lines 170-171). The authors should discuss some possible explanations for this paradoxical result.

While we can only speculate, one possibility is that serotonergic neurons modulate cardiac control in a context-dependent manner. During mechanical threat, serotonergic activity is indispensable for cardiac deceleration, perhaps by gating the threat-responsive cardiac deceleration pathway involving DA-WED. In contrast, thermogenetic activation of serotonergic neurons outside this context may recruit broader downstream targets, including circuits that induce cardiac acceleration. We have added the following sentences in our Result:

L183 – 187: “Serotonergic system, on the other hand, provided paradoxical results (Fig. 2g-k, Supplementary Fig. 5c-g): this could be because serotonergic system is required for threat-induced cardiac deceleration, while broad serotonergic activation outside this context possibly engages downstream circuits that promote cardiac acceleration.”

(4) The split-GAL4 technology is a well-established approach in *Drosophila* research. The authors don't need to go into so much detail about its mechanism in the results (lines 199-205).

We have compressed our detailed explanation about split-GAL4 technology as follows:

L212 – 214: “In this approach, GAL4 is divided into DBD and AD hemi-drivers, and functional GAL4 is reconstituted only in cells expressing both components (Fig. 3c).”
